# Microglia Transcriptome Changes in a Model of Depressive Behavior after Immune Challenge

**DOI:** 10.1371/journal.pone.0150858

**Published:** 2016-03-09

**Authors:** Dianelys Gonzalez-Pena, Scott E. Nixon, Jason C. O’Connor, Bruce R. Southey, Marcus A. Lawson, Robert H. McCusker, Tania Borras, Debbie Machuca, Alvaro G. Hernandez, Robert Dantzer, Keith W. Kelley, Sandra L. Rodriguez-Zas

**Affiliations:** 1 Department of Animal Sciences, University of Illinois Urbana-Champaign, Urbana, IL, United States of America; 2 Illinois Informatics Institute, University of Illinois Urbana-Champaign, Urbana, IL, United States of America; 3 Department of Pharmacology, University of Texas Health Science Center at San Antonio, San Antonio, TX, United States of America; 4 High-Throughput Sequencing and Genotyping Unit, Roy J. Carver Biotechnology Center, University of Illinois at Urbana-Champaign, Champaign, IL, United States of America; 5 Department of Symptom Research, University of Texas M. D. Anderson Cancer Center, Houston, TX, United States of America; 6 Integrative Immunology and Behavior Program and Department of Pathology, College of Medicine, University of Illinois at Urbana-Champaign, Champaign, IL, United States of America; 7 Department of Statistics and Carle Woese Institute for Genomic Biology, University of Illinois at Urbana-Champaign, Urbana, IL, United States of America; South Texas Veterans Health Care System and University Health Science Center San Antonio, UNITED STATES

## Abstract

Depression symptoms following immune response to a challenge have been reported after the recovery from sickness. A RNA-Seq study of the dysregulation of the microglia transcriptome in a model of inflammation-associated depressive behavior was undertaken. The transcriptome of microglia from mice at day 7 after Bacille Calmette Guérin (BCG) challenge was compared to that from unchallenged Control mice and to the transcriptome from peripheral macrophages from the same mice. Among the 562 and 3,851 genes differentially expressed between BCG-challenged and Control mice in microglia and macrophages respectively, 353 genes overlapped between these cells types. Among the most differentially expressed genes in the microglia, serum amyloid A3 (Saa3) and cell adhesion molecule 3 (Cadm3) were over-expressed and coiled-coil domain containing 162 (Ccdc162) and titin-cap (Tcap) were under-expressed in BCG-challenged relative to Control. Many of the differentially expressed genes between BCG-challenged and Control mice were associated with neurological disorders encompassing depression symptoms. Across cell types, S100 calcium binding protein A9 (S100A9), interleukin 1 beta (Il1b) and kynurenine 3-monooxygenase (Kmo) were differentially expressed between challenged and control mice. Immune response, chemotaxis, and chemokine activity were among the functional categories enriched by the differentially expressed genes. Functional categories enriched among the 9,117 genes differentially expressed between cell types included leukocyte regulation and activation, chemokine and cytokine activities, MAP kinase activity, and apoptosis. More than 200 genes exhibited alternative splicing events between cell types including WNK lysine deficient protein kinase 1 (Wnk1) and microtubule-actin crosslinking factor 1(Macf1). Network visualization revealed the capability of microglia to exhibit transcriptome dysregulation in response to immune challenge still after resolution of sickness symptoms, albeit lower than that observed in macrophages. The persistent transcriptome dysregulation in the microglia shared patterns with neurological disorders indicating that the associated persistent depressive symptoms share a common transcriptome basis.

## Introduction

Studies of behavioral and molecular changes in response to a challenge have exposed the relationship between brain inflammation and incidence of depression-like symptoms [1,2]. Peripheral infections can alter inflammatory cytokines elicited by microglia, the innate immune cells located in the brain. This alteration of cell signaling dysregulates pathways such as tryptophan metabolism that has been associated with depression-like behaviors. After peripheral challenge with Bacille Calmette-Guérin (BCG) mice display depressive-like behaviors 7 days to 1 month post challenge, well-past sickness recovery. Mice challenged with BCG exhibit sickness symptoms reflected by weight loss early in the first 2 days after challenge compared to control mice challenged with saline followed by recovery of weight by day 5. Recovery from sickness was confirmed by non-significant differences in horizontal locomotor activity and rearing at day 6 post challenge. Despite the recovery from sickness symptoms, depression-like behaviors including significant increase in the duration of immobility measured using the tail suspension test and the Porsolt forced swim test at day 6 and decrease in sucrose ingestion in the sucrose preference test at day 7 were recorded in mice challenged with BCG relative to control mice [3–5].

Brain microglia and peripheral macrophages are immune cells yet their response to immune challenge and impact on surrounding cells are different [6]. Transcriptome analysis have revealed common and unique profiles among these cell types [7]. This suggest that differences between the microglia and macrophage transcriptome could be directly associated with depression-like symptoms. Characterization of the differences between microglia and macrophage transcript isoform abundance, alternative splicing, gene differential expression, and networks after sickness recovery from BCG challenge is essential to understand the role of microglia on depression-like behaviors.

The objective of this study was to uncover the gene expression dysregulation in microglia from mice challenged with BCG that exhibit depression-like symptoms despite having recovered from the associated sickness. This work builds upon our prior study that confirmed in the same mouse populations comparable changes in body weights and other sickness indicators but significant differences in depression-like behaviors between BCG-challenged and Control groups at day 7 post-challenge [5]. Analyses supporting the objective of the present study include: 1) uncovering differential gene expression and functional categories between BCG-challenged and Control groups within cell types; 2) uncovering differential gene expression and functional categories between microglia and peripheral macrophages within BCG-challenged group; 3) detection of alternative splicing between microglia and peripheral macrophages in the BCG-challenged group; and 4) network visualization to uncover potential synergistic or antagonistic relationships between BCG-challenge and cell type groups.

## Materials and Methods

### Experiments

All animal care and experimental procedures adhered to NIH guidelines and were approved by the University of Illinois Institutional Animal Care and Use Committee. Measures were taken to minimize the number of animals used and the pain and suffering of the mice.

Microglia and peritoneal macrophages (hereby termed macrophages) were collected from approximately 22 weeks old male C57Bl/6J mice. Mice housing, management, and BCG challenge followed previously described protocols [5,8]. Briefly, mice were housed individually in standard polypropylene cages including corn cob litter. Housing was under a 12:12 h reversed light/dark cycle (lights on 10:00 PM-10:00 AM) with controlled environmental temperature (23°C) and humidity (45%). Mice were offered water and food (Teklad 8640 chow, Harlan Laboratories, Indianapolis, IN, USA) *ad libitum* and handled daily for one week prior to the trial to ensure adaptation. Mice were acclimated to the light cycle and facility for at least 3 weeks prior to the injection with BCG or saline. All mice were individually handled for a few minutes daily prior to the challenge.

The immune challenge involved live attenuated mycobacteria TICE BCG (50 mg wet weight of lyophilized culture containing 1x10^8^ colony forming units or CFU/vial; Organon Teknika Corp. LLC, USA Inc., USA). Each vial’s reconstitution prior to inoculation followed provider instructions using preservative-free saline. No peritoneal mycobacteria infection was reported in mice by day 20 after intraperitoneal infection with BCG; whereas dissemination to other organs, including the spleen, bone marrow, and lung was observed [9]. There are no reports of BCG infection of microglia or brain and the peritoneum is a source of non-infected macrophages rather than macrophages from other tissues that could be infected with BCG.

Individual mice were challenged once with either 10 mg/mouse (BCG-challenged group, n = 12) or sterile saline solution (Control group, n = 12) administered via intraperitoneal injection [5]. No mouse became severely ill or died at any time prior to the experimental endpoint. Mice were euthanized by CO_2_ asphyxiation by trained personnel 7 days after BCG challenge and all efforts were made to minimize suffering. The endpoint was selected based on prior work that demonstrated the recovery from sickness yet persistence of depressive-like symptoms 7 days after challenge [5].

Macrophages were collected from peritoneal tissue using the proven protocols [8,10]. Abdomens were disinfected, skin was retracted, and the peritoneal cavity was flushed with Hank's Balanced Salt Solution (cold harvest medium). The peritoneal fluid was centrifuged and the resulting cell pellet was resuspended and plated. The medium was aspired after 2 h incubation to remove non-adherent cells. The surviving adherent cells constituted the non-thioglycollate elicited peritoneal macrophages and cells from individual mice were stored in Trizol at -80°C until RNA extraction [11].

After peritoneum collection (~5 minutes), mice were perfused, and the brains were excised and minced. Microglia were collected following established protocols [12]. In brief, brains were trypsinized [13], dissociated using cell screens (40 μm), centrifuged, resuspended in 30% Percoll (GE Healthcare, Princeton, NJ), then centrifuged for myelin removal. Brain cells were labeled with anti-CD11b (integrin alpha M antibody) magnetized Miltenyi MicroBeads (Miltenyi Biotec, Germany). Cells were separated in a magnetic field with MS columns (Miltenyi Biotec, Germany). The resulting Cd11b^+^ fraction collected was centrifuged, resuspended, and stored at -80°C. Flow cytometry validation of cell isolation encompassed cell staining with primary fluorescent antibodies for two primary markers for macrophages and microglia: CD11b and CD45 (protein tyrosine phosphatase, receptor type, C antibody) [14,15]. Fc receptors were blocked by incubation with anti-CD16/CD32 antibody before incubation with eBioscience anti-CD11b and anti-CD45 antibodies (eBioscience Inc., San Diego, CA). Surface receptor expression was identified using a Biosciences LSR II Flow Cytometry Analyzer with BD FACSDiva software (BD Biosciences, San Jose, CA). Antibody gating was determined using isotype-stained controls. Cells were ~93% Cd11b^+^ and ~91% Cd45^+^, confirming microglia enrichment.

RNA extraction from microglia followed the Tripsin method using a total RNA Kit (Omega Biotek, Norcross, GA) and a DNase step to remove DNA contamination [11]. The Agilent 2100 Bioanalyzer with RNA Pico chip (Agilent Technologies, Palo Alto, CA) was used to assess RNA Integrity Numbers. RNA Integrity Numbers were > 9 in 90% of the samples and > 7 in 100% of the 48 samples.

### Identification of differential transcript isoform and gene expression

RNA libraries from individual mouse were sequenced using an Illumina HiSeq 2000 (Illumina, San Diego, CA) and 100nt long paired-end reads were obtained. Read quality control was implemented using FastQC [8,16]. Quality control analysis indicated that the Phred nucleotide quality score was > 30 across the length of the reads such that the read sequences were not trimmed.

Reads were mapped to the Genome Reference Consortium GRCm38 mouse assembly using Tophat2 (v 2.0.8) [17] with the Illumina iGenomes package (mm10; http://support.illumina.com/sequencing/sequencing_software/igenome.html). Reads were combined into transcripts and differential expression was tested using Cufflinks (v2.1.1) [18]. The specifications beyond the default settings used to obtain transcript abundance levels were: a) Upper Quartile Normalization was used to normalize the median transcript counts across libraries; b) multi-read correction for reads mapping to multiple sites; and c) fragment bias correction [19]. Four comparisons were evaluated: two pairwise contrasts between BGG-challenge groups within cell types, and two pairwise contrasts between cells within BCG-challenge groups. Results from genes with at least 10 mapped reads were considered. Multiple test adjustment used the Benjamini-Hoechberg false discovery rate (FDR) approach [20,21]. Genes exhibiting an FDR-adjusted P-value < 0.05 were considered differentially expressed.

### Identification of alternative splicing events

Alternative splicing events characterizing differences between groups (i.e., cell types or challenge level) were identified using a quantitative approach [22]. Quantitative characterization considered genes detected in both groups, represented by at least two transcript isoforms, and with at least one transcript isoform differentially expressed between groups (FDR-adjusted P-value < 0.05) between groups in one direction (over- or under-expressed) and the rest of the isoforms not differentially expressed or differentially expressed in the other direction.

### Identification of functional categories over-represented among gene profiles

Functional analyses of the transcript isoforms differentially expressed between BCG-challenge groups within cell types, between cell types within BCG-challenged groups, and of transcript isoforms expressed exclusively in one cell type were performed using hypergeometric testing and Gene Set Enrichment Analysis (GSEA). These analyses allowed the identification of Gene Ontology (http://www.geneontology.org/) biological processes, molecular functions, and Kyoto Encyclopedia of Genes and Genomes (KEGG) pathways (http://www.genome.jp/org/). The hypergeometric test was implemented in the Database for Annotation, Visualization and Integrated Discovery (DAVID, http://david.abcc.ncifcrf.gov) [23,24]. Gene Ontology (GO) results were reported using the DAVID Functional Annotation Tool (FAT) classes to facilitate interpretation. Category enrichment was measured using Expression Analysis Systematic Explorer (EASE) scores computed based on a one-tailed jackknifed Fisher exact test [25,26]. The GO categories were clustered and the statistical significance of each cluster was assessed using an Enrichment Score (-log_10_ geometric mean of the cluster members EASE scores [27,28]. Functional annotation clusters were considered significant at Enrichment Score > 2 (comparable to P-value < 0.001) using the *Mus musculus* genome as background. Gene Set Enrichment Analysis was implemented using the GSEA-P software package [29,30]. This approach offered functional insights complementary to DAVID based on the consideration of the expression profile of all the genes analyzed and annotations to the Molecular Signature Database (MSigDB) [31].

### Gene network visualization

Interpretation of findings and discovery of differences in gene co-expression associated with BCG challenge or cell type were enhanced using network visualization. Networks were visualized using the BisoGenet plug-in [27,28,32] within the Cytoscape environment [24,33]. BisoGenet enabled the visualization of associations between genes detected in the present study using information from the SysBiomics repository. Networks depicting genes as nodes and gene associations as edges and including at most one non-measured gene connecting observed genes were considered. The resulting gene networks were augmented with information on differential gene expression results from the comparison between BCG-challenged and cell type groups obtained in this study. The node size represented the differential expression P-value and the node color denoted over- or under-expression between challenge levels or cell types.

## Results and Discussion

### Global transcriptome profiles across immune challenge groups and cell types

Among more than 15,000 genes tested, 562 and 3,851 were differentially expressed (FDR-adjusted P-value < 0.05) between the BCG-challenged and Control groups in microglia and macrophages, respectively (Table 1). Differential abundance of transcript isoforms between BCG-challenged groups followed similar patterns *albeit* at lower absolute count number. Significant gene expression differences between cell types were more prevalent and extreme than between BCG-challenged groups. Approximately 9,117 genes (9,780 transcript isoforms) were differentially expressed between microglia and macrophages in the BCG-challenged group. Among these, 6,590 genes (8,144 transcripts) overlapped between cell types across BCG-challenged groups. The prevalent differential expression between cell types is consistent with prior transcriptomic comparisons and has been linked to differences in cell type origin and local environment [7,34,35].

**Table 1:** Number of genes and transcript isoforms analyzed and differentially expressed between BCG-challenged (BCG) and Control mice within cell type and between microglia and macrophages within challenge group and overlapping counts.

Comparison	Genes			Transcript isoforms		
	Analyzed	DE^1^ or Unique Annotated	DE or Unique Not annotated	Analyzed	DE or Unique Annotated	DE or Unique Not annotated
Microglia Control vs. BCG	15,293	562	18	39,005	317	6
Macrophages Control vs. BCG	13,245	3,851	286	32,743	2,381	160
Overlap		353			245	
Control Microglia vs Macrophages	15,287	9,860	0	29,110	11,113	0
BCG Microglia vs. Macrophages	15,828	9,117	890	42,035	9,780	735
Overlap		6,590			8,144	
Unique to Macrophages Control		23			42	
Unique to Microglia Control		236			421	
Unique to Macrophages BCG		3			20	
Unique to Microglia BCG		214			300	

DE^1^: differentially expressed genes or transcript isoforms (FDR-adjusted P-value < 0.05) or uniquely detected in either cell type.

### Differences in the microglia transcriptome between BCG-challenged and Control mice

Differential gene and transcript isoform expression in microglia between BCG-challenged and Control mice are presented in Table 1. Among the differentially expressed genes in the microglia, 518 genes were over-expressed whereas only 44 genes were under-expressed in the BCG-challenged relative to Control mice. The predominance of over-expressed genes (92%) in response to BCG-challenge is consistent with previous reports of transcriptome changes in the microglia in response to lipopolysaccharide (LPS) challenge [36]. Differentially expressed genes between BCG-challenged and Control in microglia ((|log_2_(fold change)| > 3, FDR-adjusted P-value < 2.0 x 10^−3^) are listed in Table 2 together with supporting literature. An extended list of differentially expressed genes in microglia is provided in S1 Table.

**Table 2:** Most extreme differentially expressed genes between BCG-challenged and Control mice within microglia (|log_2_(fold change)| > 3, FDR-adjusted P-value < 2.0 x 10^−3^) and macrophages (|log_2_(fold change)| > 6, FDR-adjusted P-value < 3.0 x 10^−4^) and supporting literature review.

Gene Symbol	Gene name NCBI	Log_2_(BCG/ Control)	References
Microglia; BCG-challenged vs Control mice			
Saa3	serum amyloid A 3	5.90	[37–40]
Cadm3	cell adhesion molecule 3	5.14	[37]
Steap4	STEAP family member 4	4.89	[41,42]
Sele	selectin E	4.80	[43,44]
Cxcr1	chemokine (C-X-C Motif) receptor 1	4.03	[39,45,46]
Ifitm1	interferon induced transmembrane protein 1	3.72	[38,39,44,47]
Irg1	immunoresponsive 1 homolog: involved in the inhibition of the inflammatory response.	3.66	[46,48,49]
Lrg1	leucine-rich alpha-2-glycoprotein 1	3.57	[40,50]
Prok2	prokineticin 2	3.48	[50]
Cfb	complement factor B	3.39	[38]
Slfn4	schlafen 4	3.33	[40,48]
Nxpe5	neurexophilin and PC-esterase domain family, member 5	3.29	[51]
Ly6i	lymphocyte antigen 6 complex, locus I	3.29	[38,49]
Oas3	2'-5'-oligoadenylate synthetase 3, 100kDa	3.27	[38,52]
Ifi205	interferon activated gene 205	3.15	[38,53]
Stfa1	stefin A1	3.12	[54]
Plac8	placenta-specific 8	3.09	[38,44]
Macrophages; BCG-challenged vs Control mice			
S100a9	S100 calcium binding protein A9 (calgranulin B)	10.11	[55]
Mrgpra2a	MAS-related GPR, member A2A	10.10	
Ly6i	lymphocyte antigen 6 complex, locus I	9.42	
Asprv1	aspartic peptidase, retroviral-like 1	9.15	
Ly6c2	lymphocyte antigen 6 complex, locus C2	7.92	[56]
Nos2	nitric oxide synthase 2, inducible	7.69	[57]
Il1f9	interleukin 1 family, member 9	7.66	[58]
Ccl8	chemokine (C-C motif) ligand 8	7.45	[41]
2010002M12Rik	interferon induced protein with tetratricopeptide repeats 1B like 2	7.32	
Spon1	spondin 1, (f-spondin) extracellular matrix protein	7.23	[59]
S100a8	S100 calcium binding protein A8 (calgranulin A)	7.15	[60]
Cxcr2	chemokine (C-X-C motif) receptor 2	7.02	
Ifng	interferon gamma	6.45	[61]
Cxcl9	chemokine (C-X-C motif) ligand 9	6.40	
Gpr141	G protein-coupled receptor 141	6.15	
2010005H15Rik	RIKEN cDNA 2010005H15 gene	6.14	
Iigp1	interferon inducible GTPase 1	6.08	

Studies of behavioral and molecular changes in response to BCG challenge have exposed the relationship between brain inflammation and incidence of depression-like symptoms [1,2]. These studies have reported increased levels of inflammatory cytokines expression in the microglia. Similarly, the present study detected over-expression of genes related to inflammatory response 7 days after challenge (Table 2, S1 Table) including Serum amyloid A3 (Saa3), S100 calcium binding protein A8 (S100a8), S100 calcium binding protein A9 (S100a9), Fc-gamma receptors (Fcgr4 and Fcgr2b), prostaglandin-endoperoxide synthase 2 (Ptgs2), interleukin 1beta (Il1b), and interleukins receptors (Il1r1 and Il4ra).

Consistent with our findings, an immune challenge event can elicit over-expression of Saa3 in the brain. Also, the levels of Saa3 in microglia increase in response to mouse hepatitis virus-JHM [62] and a strain of Creutzfeldt-Jakob disease [63]. The expression of Saa3 could be induced by S100A8 and S100A9 and these were over-expressed as well. Altered expression of S100A9 has been reported in neurological diseases associated with inflammation and depression such as cerebral ischemia, traumatic brain injury, and Alzheimer’s disease [64,65].

Fc receptors are expressed in immune cells and connect humoral and cell-mediated response to pathogen infection [66]. Polymorphism in these receptors were associated with susceptibility to Guillain-Barré syndrome and multiple sclerosis, disorders that exhibit depression symptoms [67]. Increased levels of Fcgr4 and Fcgr2b expression, similar to that observed in this study, induce vascular damage and exacerbate neurodegenerative conditions in humans [68]. Activation of microglia through Fc gamma receptors results in phagocytosis and polarization to an M2b phenotype [68] and similarly, Fc receptor overexpression was correlated with over-expressed pro-inflammatory cytokines in the present and previous studies [69,70]. Similarly, Ptgs2 was over-expressed in the microglia in our study and has been related to inflammatory response in the brain and to neurodegeneration processes [71]. This gene has been proposed as a therapeutic target for neurodegenerative diseases that encompass depressive behaviors such as Parkinson, Alzheimer, and Huntington’s disease [72].

Over-expression of pro-inflammatory cytokines such as interleukin Il1b has been linked to neurodegenerative disorders such as Alzheimer’s disease [65,73]. Il1b has been linked to depressive behaviors [74,75] and therapies blocking Il1b expression in a mouse model of Alzheimer’s disease decreased the synthesis of S100 proteins, decreased fibrillar deposition, and protected mice from cognitive deficits [76]. Our transcriptome findings in the microglia are consistent with reports that mice express depression-like behaviors 7 days after of inoculation with BCG [5,77].

### Differences in the macrophage transcriptome between BCG-challenged and Control mice

The comparison of gene expression profiles in macrophages between BCG-challenged and Control mice enabled us to uncover changes common to macrophage and microglia immune cells and changes unique to microglia cells. Differential gene and transcript isoform expression between BCG-challenged and Control mice in macrophages are listed in Table 1. Among the 3,851 differentially expressed genes in macrophages, 2,151 were over-expressed and 1,700 were under-expressed in the BCG-challenged relative to Control mice. The predominance of over-expressed genes (75%) in response to BCG-challenge is consistent with previous reports of transcriptome changes in the macrophages in response to BCG challenge [78] and consistent with the microglia changes previously described. Among these genes S100A9, S100A8, Il1b, interferon gamma (Ifng), nitric oxide synthase 2 inducible (Nos2), and interleukin 1 family member 9 (Il1f9) were identified. Like in microglia, many differentially expressed genes in macrophages were related with inflammatory response. Differentially expressed genes between BCG-challenged and Control in macrophages (|log_2_(fold change)| > 6, FDR-adjusted P-value < 3.0 x 10^−4^) are listed in Table 2 together with supporting literature. An extended list of differentially expressed genes in macrophages is provided in S1 Table.

The increase in expression of Ifng and Nos2 is a component in the macrophage response to infectious or inflammatory diseases represented in our study by the BCG challenge [61,79]. High levels of Ifng were found in patients diagnosed with major depression without pathogen stimuli [80–84]. Additionally, an increase in Ifng expression up-regulates Caspase 1 (Casp1), an enzyme that converts pro-Il1b into active mature Il1b [85] and that has a role in acute [86] and major depression [74,75]. Our results corroborated that the up-regulation of Ifng and Il1b (Table 2, S1 Table) is correlated with the development of depression-like behavior in BCG-challenged mice [5,77].

### Transcriptome differences between BCG-challenged and Control mice shared between cell types

Among the genes differentially expressed between BCG-challenged and Control mice, 353 genes overlapped between microglia and macrophages (Table 1). Shared profiles were identified in genes belonging to families involved in immune response including: S100A8, S100A9, Il1r1, and Il1b. Kynurenine 3-monooxygenase (Kmo) was over-expressed (FDR-adjusted P-value < 0.05) in BCG-challenged relative to Control mice, both in microglia and macrophages (S1 Table). Kmo catalyzes the conversion of kynurenine to 3-hydroxykynurenine and modulation of KMO activity has been implicated in several neurodegenerative diseases [87]. Over-expression of Il1b and Kmo was observed in the microglia of mice that also exhibited social withdrawal after LPS injection [88]. Also, over-expression of Kmo was reported in the rat whole brain after a systemic challenge with LPS [89,90] and in human brain cells after Il1b treatment [91]. On the other hand, a study of mouse whole brain after BCG-challenge reported over-expression of indoleamine 2,3-dioxygenase 1 (Ido1) but no differential expression of Kmo [92]. Both, Kmo and Ido1 reduce the level of circulating tryptophan and production of serotonin and increase the levels of tryptophan metabolites that have cytotoxic effect [1,2]. In the present study, Kmo was over-expressed but Ido1 and the ortholog Ido2 were not differentially expressed in the microglia between BCG-challenged and Control mice. Our study supports the emerging notion that Kmo could be induced by immune challenge [93,94] and, as potential target of inflammatory cytokines in the kynurenine pathway, Kmo could be associated with behavioral disorders.

### Functional analysis of microglia and macrophages transcriptomic differences between BCG-challenged and Control mice

Analysis of enriched functional categories based on the comparison of genes expression profiles between BCG-challenged and Control mice in microglia and macrophages offered insights into the biological processes and pathways associated with BCG-challenge. Table 3 summarizes the clusters of functional categories surpassing a DAVID Enrichment Score > 4. S2 Table presents the complete list of categories that exhibited an Enrichment Score > 2 and corresponding gene count.

**Table 3:** Most significant clusters (DAVID Enrichment Score ES > 4) of enriched Gene Ontology (GO) biological processes (BP) and molecular functions (MF) among the genes differentially abundant between BCG-challenged and Control mice within cell type.

Cell type and cluster^1^	Terms^2^	ES
Microglia		
BP	~defense response ~inflammatory response ~response to wounding	12.96
BP	~chemotaxis ~taxis ~locomotory behavior	5.63
MF	~carbohydrate binding ~polysaccharide binding ~pattern binding	5.12
MF	~peptidase inhibitor activity ~endopeptidase inhibitor activity ~enzyme inhibitor activity	4.88
BP	~immune effector process ~adaptive immune response ~adaptive immune response based on somatic recombination of immune receptors built from immunoglobulin superfamily domains	4.75
BP	~cell adhesion ~biological adhesion ~cell-cell adhesion	4.37
Macrophages		
BP	~defense response ~response to wounding ~inflammatory response	13.32
MF	~carbohydrate binding ~polysaccharide binding ~pattern binding	8.18
BP,MF	~chemotaxis ~taxis ~chemokine activity	7.79
BP	~cell activation ~leukocyte activation ~lymphocyte activation	6.17
BP	~vasculature development ~blood vessel development ~blood vessel morphogenesis	6.12
BP	~regulation of cytokine production ~positive regulation of cytokine production ~positive regulation of multicellular organismal process	5.97
BP	~positive regulation of immune system process ~positive regulation of response to stimulus ~positive regulation of immune response	5.86
BP	~immune effector process ~leukocyte mediated immunity ~adaptive immune response	5.02
BP	~apoptosis ~programmed cell death ~cell death	4.77
BP	~regulation of phosphorylation ~regulation of phosphorus metabolic process ~regulation of phosphate metabolic process	4.34
BP,MF	~GTPase regulator activity ~nucleoside-triphosphatase regulator activity ~GTPase activator activity	4.20
BP	~positive regulation of immune system process ~regulation of leukocyte activation ~regulation of cell activation	4.01

^1^ Each row corresponds to a cluster of Functional Annotation Tool (FAT) GO categories.

^2^ The three GO terms exhibiting most significant enrichment P-value in each cluster are listed, separated by “~”. Additional information in each cluster is provided in S2 Table.

Enriched functional categories shared by both cell types included immunological response, chemotaxis, cell migration and apoptosis among others biological processes and molecular functions (Table 3, S2 Table). These clusters with highest enrichment overlap between microglia and macrophages, corroborating the persistence of systemic response 7 days after BCG-challenge. The enrichment of apoptosis-related processes observed in this and previous studies [95] is associated with the over-expression of Nos2 in both cell types (Table 2). Likewise, the enrichment of monocyte and leukocyte migration processes (Table 3) reported here and by others [96] is in correspondence with the over-expression of S100A9 and S100A8 (Table 2) in both cell types. Higher leukocyte migration, activity of the pro-inflammatory cytokines [97], and apoptosis [98] were all observed in patients with major depression disorders. Additionally, the observed enrichment of protein metabolism including inhibition of enzymes and peptidases is compatible to changes in protein metabolism and downregulation of proteolysis reported in patients with bipolar depression [99,100] and schizophrenia [101].

Complementary functional analysis of the microglia and macrophages profiles using GSEA (Tables 4 and 5, respectively) confirmed the previous gene list enrichment and further enabled the discrimination of enrichment among over- and under-expressed genes in BCG-challenged relative to Control mice. S3 and S4 Tables include additional categories enriched at P-value < 0.05 and with a minimum of 10 genes in microglia and macrophages, respectively. A remarkable GSEA finding in microglia was the downregulation of inositol metabolism because this result is consistent with changes in this metabolite in schizophrenia, bipolar, and depression disorders [101,102].

**Table 4:** Gene Set Enrichment Analysis (GSEA) categories enriched among transcript isoforms over-expressed (FDR-adjusted P-value < 5.0 x 10^−4^ > 10 transcript isoforms) and under-expressed (P-value < 0.05 > 10 transcript isoforms) in BCG-challenged relative to Control mice in microglia.

Categories	NG^1^	P-value	FDR ^2^
Over-expressed in BCG-challenged vs Control mice			
Defense response	147	<0.1E-05	<0.1E-05
Inflammatory response	79	<0.1E-05	<0.1E-05
Response to other organism	41	<0.1E-05	<0.1E-05
Immune response	152	<0.1E-05	<0.1E-05
Response to virus	31	<0.1E-05	<0.1E-05
Response to external stimulus	183	<0.1E-05	<0.1E-05
Immune system process	219	<0.1E-05	<0.1E-05
Response to wounding	113	<0.1E-05	<0.1E-05
KEGG cytokine cytokine receptor interaction	146	<0.1E-05	<0.1E-05
Multi organism process	77	<0.1E-05	<0.1E-05
Response to biotic stimulus	69	<0.1E-05	<0.1E-05
Locomotory behavior	57	<0.1E-05	7.98E-05
Cation homeostasis	58	<0.1E-05	8.65E-05
Cellular cation homeostasis	56	<0.1E-05	1.49E-04
G protein coupled receptor binding	30	<0.1E-05	2.80E-04
Ion homeostasis	65	<0.1E-05	3.30E-04
Under-expressed in BCG-challenged vs Control mice			
Nervous system development	228	<0.1E-05	1.00E+00
Polysaccharide binding	19	2.85E-03	7.91E-01
Glycosaminoglycan binding	18	5.46E-03	5.51E-01
KEGG inositol phosphate metabolism	42	8.10E-03	7.12E-01
Central nervous system development	73	1.24E-02	9.92E-01
Protein secretion	16	1.43E-02	6.71E-01
Transmission of nerve impulse	85	2.27E-02	6.72E-01
KEGG Parkinson’s disease	94	3.03E-02	7.31E-01
Cellular protein complex assembly	30	4.28E-02	1.00E+00
Synaptic transmission	75	4.76E-02	8.86E-01

^1^ NG: number of genes.

^2^ FDR: adjusted P-value.

**Table 5:** Gene Set Enrichment Analysis (GSEA) categories enriched among transcript isoforms over-expressed (FDR-adjusted P-value < 1.5 x 10^−2^ > 10 transcript isoforms) and under- expressed (FDR-adjusted P-value < 5 x 10^−4^ > 10 transcript isoforms) in BCG-challenged relative to Control mice in macrophages.

Categories	NG^1^	P-value	FDR^2^
Over-expressed in BCG-challenged relative to Control mice			
KEGG ECM receptor interaction	45	<0.1E-05	9.96E-04
Defense response	144	<0.1E-05	5.88E-03
Inflammatory response	73	<0.1E-05	1.28E-02
Serine type peptidase activity	18	<0.1E-05	1.31E-02
Serine hydrolase activity	19	<0.1E-05	1.32E-02
KEGG proteasome	43	<0.1E-05	1.48E-02
Under-expressed in BCG-challenged relative to Control mice			
KEGG ribosome	78	<0.1E-05	<0.1E-05
Structural constituent of ribosome	75	<0.1E-05	<0.1E-05
Gated channel activity	27	<0.1E-05	<0.1E-05
Cation channel activity	29	<0.1E-05	2.39E-04

^1^ NG: number of genes.

^2^ FDR: adjusted P-value.

### Gene networks of microglia and macrophages transcriptomic differences between BCG-challenged and Control mice

**Fig 1:**
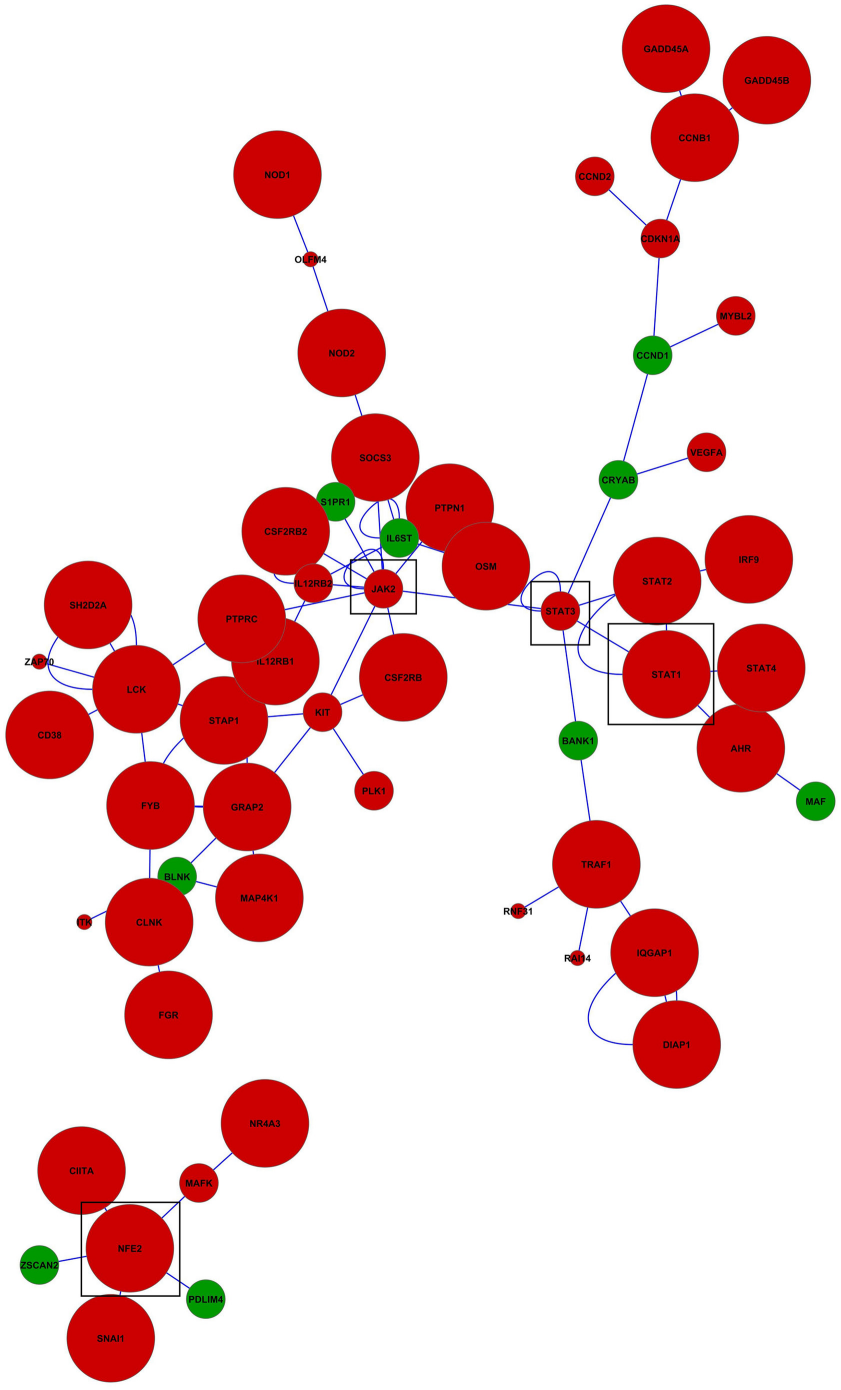
Network of genes differentially expressed between BCG-challenged and Control mice in microglia. Red (green) nodes denote genes over- (under-) expressed in BCG-challenged relative to Control mice. Node size represents the P-value were larger nodes indicates more extreme significance (FDR-adjusted P-value < 0.001 larger nodes; P-value < 0.05 intermediate nodes; P-value < 0.1 small nodes). Edges denote known relationships between genes in the SysBiomics repository. Framed genes (squares) are discussed in the manuscript.

**Fig 2:**
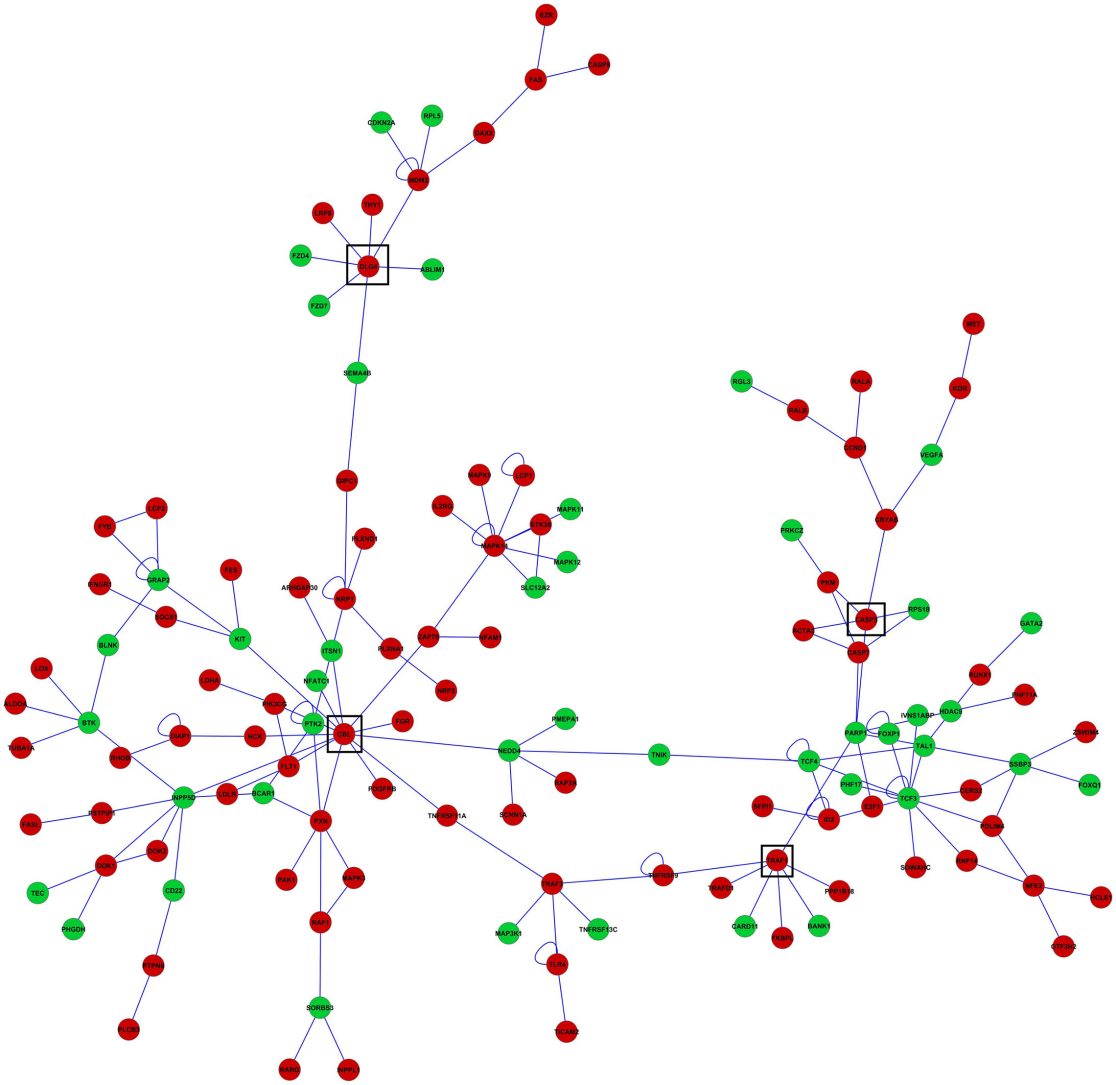
Network of genes differentially expressed between BCG-challenged and Control mice in macrophages. Red (green) nodes denote genes over- (under-) expressed in BCG-challenged relative to Control mice. All genes were differentially expressed at FDR-adjusted P-value < 0.001. Edges denote known relationships between genes in the SysBiomics repository. Framed genes (squares) are discussed in the manuscript.

Visualization of networks of genes differentially expressed between BCG-challenged and Control mice in microglia (Fig 1) and macrophages (Fig 2) augmented our understanding of the molecular relationships among genes. Networks including more than 5 connected genes are discussed. The predominance of over-expressed genes relative to under-expressed genes in both networks is in agreement with their predominance among the most differentially expressed genes in Table 2 and with the enriched categories detected using GSEA (Tables 4 and 5). These networks also exhibit a predominance of genes related to immune response.

The microglia network (Fig 1) includes 57 gene nodes and 84% of the genes were over-expressed in BCG-challenged relative to Control (most at FDR-adjusted P-value < 0.001). In this network, Janus kinase 2 (Jak2), signal transducer and activator of transcription 3 (Stat3), signal transducer and activator of transcription 1 (Stat1), and nuclear factor erythroid derived 2 (Nfe2) were well-connected gene nodes linking to 11, 7, 5, and 5 other gene nodes, respectively. These genes were over-expressed in BCG-challenge relative to Control mice. Stat1 was affiliated to the enriched category “response to bacterium” in functional cluster 1 (Table 3) whereas nuclear factor erythroid derived 2 (Nfe2) was also over-expressed in BCG-challenge relative to the Control mice, although the corresponding categories were enriched below the set threshold.

The macrophages network (Fig 2) included 129 gene nodes and 66% of the genes were over-expressed in BCG-challenged relative to Control (FDR-adjusted P-value < 0.001). Casitas B-lineage lymphoma (Cbl) was the most connected gene node, linked to 13 other nodes. This gene was over-expressed in BCG-challenged relative to Control mice yet was not affiliated to the functional categories enriched beyond the set threshold. Discs large homolog 4 (Dlg4) and tumor necrosis factor receptor-associated factor 1 (Traf1) were over-expressed genes in BCG-challenged relative to Control mice and each gene was connected to 7 gene nodes. Dlg4 was affiliated to enriched categories including behavior, endocytosis, and structural molecule activity across 3 functional clusters whereas Traf1 was affiliated to enriched categories related to apoptosis across 2 functional clusters (Table 3). Caspase 3 (Casp3) was linked to 5 other gene nodes and was also over-expressed in BCG-challenged relative to Control mice. Casp3 was affiliated to several enriched categories including apoptosis, kinase activities, and cells activation across 6 functional clusters (Table 3).

### Transcriptome differences between microglia and macrophages from BCG-challenged mice

The study of transcript isoforms and genes differentially expressed between microglia and macrophages 7 days after BCG challenge offered additional insights into the roles of both cell types after immune challenge (Table 1). Over 9,780 transcript isoforms corresponding to 9,117 genes were differentially expressed between microglia and macrophages in BCG-challenged mice. The genes most differentially expressed between microglia and macrophages from BCG-challenged mice (log_2_(fold change) > |6|, FDR-adjusted P-value < 2.0 x 10^−4^) are listed in Table 6 together with supporting literature review. An extended list of differentially expressed genes between microglia and macrophages is provided in S5 Table.

**Table 6:** Most extreme differentially expressed genes (FDR-adjusted P-value < 2.0 x 10^−4^) between microglia and macrophages in BCG-challenged mice and supporting literature review.

Gene Symbol	NCBI Gene Name	Log_2_(Macrophages/Microglia)	Reference
Col8a1	collagen, type VIII, alpha 1	-13.70	[103]
Kcne2	potassium voltage-gated channel, Isk-related subfamily, gene 2	-13.63	[104]
Slc38a5	solute carrier family 38, member 5	-11.61	
Lrrc3	predicted gene 884	-11.49	[105]
Nav3	neuron navigator 3	-11.27	[106]
Gm694	predicted gene 694	-11.07	
Capn3	calpain 3	-11.06	[107]
Gpr34	G protein-coupled receptor 34	-10.49	[39,105,106]
Lrrn1	leucine rich repeat protein 1, neuronal	-10.30	[108]
Wwox	WW domain-containing oxidoreductase	-10.24	
Vstm4	V-set and transmembrane domain containing 4	-10.07	
Ecscr	endothelial cell surface expressed chemotaxis and apoptosis regulator	-9.95	[39]
Cenpf	centromere protein F	-9.93	
Gm10790	predicted gene 10790	-9.93	
Tspan7	tetraspanin 7	-9.79	[105]
Epn3	epsin 3	-9.76	
Tmem100	transmembrane protein 100	-9.73	[105]
Leprel1	prolyl 3-hydroxylase 2	-9.72	[105]
Ebf3	early B cell factor 3	-9.71	[105]
Rnu11	U11 small nuclear RNA	-9.65	
Wdr52	cilia and flagella associated protein 44	-9.60	
Olfml3	olfactomedin-like 3	-9.60	[39,105]
Trpm3	transient receptor potential cation channel, subfamily M, member 3	-9.58	
Fat3	FAT tumor suppressor homolog 3 (Drosophila)	-9.56	
Adcy2	adenylate cyclase 2	-9.52	
Kcnj13	potassium inwardly-rectifying channel, subfamily J, member 13	-9.48	
Gpr56	adhesion G protein-coupled receptor G1	-9.46	[39,105]
Sema4g	sema domain, immunoglobulin domain (Ig), transmembrane domain (TM) and short cytoplasmic domain, (semaphorin) 4G	-9.45	
Ecm1	extracellular matrix protein 1	6.89	[39]
F10	coagulation factor X	6.90	
Spib	Spi-B transcription factor (Spi-1/PU.1 related)	6.90	
Cxcr5	chemokine (C-X-C motif) receptor 5	6.98	[39]
Cxcl3	chemokine (C-X-C motif) ligand 3	7.07	[39]
Flrt3	fibronectin leucine rich transmembrane protein 3	7.14	[39]
Selplg	P-selectin glycoprotein ligand 1	7.16	[39]
Cd19	CD19 antigen	7.17	[39]
Retnla	resistin like alpha	7.25	[39]
Tnfrsf8	tumor necrosis factor receptor superfamily, member 8	7.42	
Cd79a	CD79A antigen (immunoglobulin-associated alpha)	7.49	
Mcoln2	mucolipin 2	7.54	
Vsig4	V-set and immunoglobulin domain containing 4	7.55	
Fcrla	Fc receptor-like A	7.64	
Ms4a1	membrane-spanning 4-domains, subfamily A, member 1	7.68	
Cd163l1	CD163 molecule-like 1	7.76	
Gjb5	gap junction protein, beta 5	7.86	
Vmn2r24	vomeronasal 2, receptor 24	7.86	
Scn4a	sodium channel, voltage-gated, type IV, alpha	7.88	
Il9r	interleukin 9 receptor	7.95	
Blk	B lymphoid kinase	8.01	
F7	coagulation factor VII	8.11	[40]
Fcrl5	Fc receptor-like 5	8.14	
Serpinb2	serine (or cysteine) peptidase inhibitor, clade B, member 2	8.21	[39]
Pou2af1	POU domain, class 2, associating factor 1	8.44	
Ptges	prostaglandin E synthase	8.66	[109]
Cxcl13	chemokine (C-X-C motif) ligand 13	9.30	[39]
Nos2	nitric oxide synthase 2, inducible	9.33	[79]
Cd5l	CD5 antigen-like	9.34	[39]
Arg1	arginase, liver	10.05	[110]

The list of genes over-expressed in microglia relative to macrophages from BCG-challenged mice confirms the similar molecular mechanisms shared between neurological disorders and response to immune challenge that also share depressive behaviors. Among the differentially expressed genes, collagen type VIII alpha 1 (Col8a1) and potassium voltage-gated channel Isk-related subfamily gene 2 (Kcne2) were over-expressed meanwhile arginase liver (Arg1) and Nos2 were under-expressed in microglia relative to macrophages from BCG-challenged mice. Col8a1 is up-regulated during repair processes in the mouse brain and collagen subunits have been linked to axonal guidance, synaptogenesis and Schwann cell differentiation [111,112]. Genomic rearrangements involving this gene have been linked to Tourette syndrome, a neuropsychiatric disorder that can encompass depression symptoms [113]. In addition to Col8a1, other genes over-expressed in microglia relative to macrophages from BCG-challenged mice have been linked to neuropsychiatric disorders that encompass depression symptoms (Table 6) including: leucine rich repeat protein 1 neuronal (Lrrn1), tetraspanin 7 (Tspan7), early B cell factor 3 (Ebf3), neuron navigator 3 (Nav3), and WW domain-containing oxidoreductase (Wwox). Over-expression of Lrrn1 was linked to autism spectrum disorder and Tourette syndrome [114]. Tspan7 was associated with Huntington's chorea, fragile X syndrome, and myotonic dystrophy [115]. Changes in the expression of Ebf3 was observed in obsessive-compulsive disorder [116]. Nav3 and WW domain-containing oxidoreductase (Wwox) have been implicated in Alzheimer's disease [117,118]. Nav3 was also associated with amyotrophic lateral sclerosis, Parkinson׳s disease [118], and was up-regulated in microglia compare to macrophages in response to hypoxia after stroke [106].

Kcne2 was among the genes over-expressed in microglia compared to macrophages in BCG-challenged mice. Expression of K+ channel genes follows nitric oxide changes after LPS stimulation indicating that K+ channels are involved in microglia activation [104]. Also, sialic acid binding Ig-like lectin H (Siglech) is considered a microglia signature gene [105] and G protein-coupled receptor 34 (Gpr34) is highly expressed in microglia and regulates the function, morphology, and phagocytosis of microglia during neuroinflammation [119,120].

Among the genes under-expressed in microglia relative to macrophages from BCG-challenged mice, the enzymes encoded by Arg1 and Nos2 compete for the same substrate: L-arginine [121,122]. The over-expression of Nos2 in macrophages is controlled by cytokines in response to a pathogen or inflammatory diseases [79] and also up-regulation of Nos2 and Arg1 were found in mouse and rat macrophages treated with LPS [123]. MicroRNA 155 (Mir155) was also over-expressed in macrophages relative to microglia (S5 Table). This microRNA is a crucial regulator of apoptosis and cell fate decisions in BCG-challenged macrophages and over-expression triggers mitogen-activated protein kinases (MAPK) cascades [124]. The genes under-expressed in microglia relative to macrophages after BCG challenge offer insights into the molecular mechanisms that are differentially regulated due to their potential negative impact in the brain.

### Functional analysis of transcriptomic differences between microglia and macrophages from BCG-challenged mice

Enriched functional categories offered additional insights into the roles of microglia and macrophages after sickness recovery from a BCG challenge. Table 7 summarizes the DAVID clusters of functional categories surpassing Enrichment Score > 4 and S6 Table presents the complete list of categories surpassing Enrichment Score > 2 and corresponding gene counts. Functional categories encompassing genes differentially expressed between cell types included leukocyte regulation and activation, chemokine and cytokine activities, MAP kinase activity, and apoptosis (Table 7). These results are consistent with reports of enriched categories in LPS-challenged microglia [125] and BCG-challenged macrophages [124]. Enrichment of apoptosis (Table 7) is associated with the over-expression of Nos2 and Arg1 in macrophages because these genes regulate the production of peroxynitrites that in turn induce apoptosis [126,127].

**Table 7:** Most significant clusters (DAVID Enrichment Score ES > 4) of enriched Gene Ontology (GO) biological processes (BP) and molecular functions (MF) among the transcript isoforms differentially abundant between microglia and macrophages from BCG-challenged mice.

GO cluster^1^	Terms^2^	ES
BP	~response to wounding~inflammatory response~defense response	8.72
BP	~regulation of cell activation~regulation of leukocyte activation~positive regulation of immune system process	6.72
BP	~cell migration~localization of cell~cell motility	6.55
BP	~regulation of cytokine production~regulation of cytokine biosynthetic process~positive regulation of cytokine biosynthetic process	6.44
MF,BP	~purine nucleotide binding~ribonucleotide binding~purine ribonucleotide binding	6.14
MF,BP	~taxis~chemotaxis~chemokine activity	6.04
BP	~cell death~death~programmed cell death	5.92
BP	~regulation of apoptosis~regulation of programmed cell death~regulation of cell death	5.53
BP	~regulation of cell activation~regulation of leukocyte activation~regulation of lymphocyte activation	5.26
BP	~vasculature development~blood vessel development~blood vessel morphogenesis	4.59
BP	~hemopoietic or lymphoid organ development~immune system development~hemopoiesis	4.59
BP	~regulation of cytokine production~regulation of interferon-gamma production~positive regulation of cytokine production	4.26
MF	~GTP binding~guanyl ribonucleotide binding~guanyl nucleotide binding	4.07

^1^ Each row corresponds to a cluster of Functional Annotation Tool (FAT) GO categories.

^2^ The three GO terms exhibiting most significant enrichment P-value in each cluster are listed, separated by “~”. Additional information in each cluster is provided in S6 Table

Enrichment of categories within over- or under-expressed genes in microglia relative to macrophages detected using GSEA offered confirmatory and complementary information. Table 8 lists GSEA findings at FDR-adjusted P-value < 0.01 and including > 10 transcript isoforms and S7 Table includes a more extensive list of categories enriched at FDR-adjusted P-value < 0.05 and including > 10 transcript isoforms per category. The enrichment of categories associated with immune response, ribosome and cytokine activity among genes under-expressed in microglia relative to macrophages could be related to the potential detrimental neurological effect of microglia activation [128]. Among the enriched categories including genes over-expressed in the microglia, dysregulation of the tight junction pathway has been related to anxiety behaviors and altered signaling in the brain [129]. Similarly enriched categories included brain, central and nervous system development categories are consistent with reports of functional signatures in microglia [105].

**Table 8:** Gene Set Enrichment Analysis (GSEA) categories enriched (FDR-adjusted P-value < 0.01, > 10 transcript isoforms) among transcript isoforms under-expressed in microglia relative to peripheral macrophages in BCG-challenged mice.

Category	NG^1^	P-value	FDR^2^
Adaptive immune response	21	<0.1E-05	<0.1E-05
Adaptive immune response	20	<0.1E-05	<0.1E-05
Positive regulation of immune response	20	<0.1E-05	<0.1E-05
Regulation of immune response	22	<0.1E-05	<0.1E-05
Regulation of immune system process	46	<0.1E-05	1.05E-03
Chemokine receptor binding	28	<0.1E-05	1.18E-03
Positive regulation of immune system process	37	<0.1E-05	1.34E-03
Positive regulation of response to stimulus	27	<0.1E-05	1.57E-03
KEGG primary immunodeficiency	30	<0.1E-05	1.88E-03
KEGG ribosome	81	<0.1E-05	1.90E-03
Regulation of lymphocyte activation	28	<0.1E-05	2.07E-03
Cellular defense response	38	<0.1E-05	2.28E-03
Lymphocyte activation	47	<0.1E-05	3.78E-03
G protein coupled receptor binding	35	<0.1E-05	4.34E-03
Chemokine activity	27	<0.1E-05	4.62E-03
Positive regulation of multicellular organismal process	47	<0.1E-05	4.63E-03
Cytokine activity	61	<0.1E-05	6.65E-03
B cell activation	18	<0.1E-05	7.59E-03
Locomotory behavior	61	<0.1E-05	8.08E-03
Leukocyte activation	51	<0.1E-05	8.95E-03
KEGG hematopoietic cell lineage	52	<0.1E-05	9.40E-03

^1^ NG: number of genes.

^2^ FDR: adjusted P-value.

### Alternative splicing between microglia and macrophages from BCG-challenged mice

Significant evidence of alternative splicing was detected in the comparison between cell types relative to challenge levels. Table 9 summarizes genes with at least nine transcript isoforms and at least two differentially expressed (FDR-adjusted P-value < 0.05) between microglia and macrophages. S8 Table lists all genes that match the broader alternative splicing definition of at least one transcript isoform differentially expressed among multiple isoforms.

**Table 9:** Genes exhibiting an alternative splicing event between microglia and macrophages in BCG-challenged mice including at least nine transcript isoforms and at least two over- or under-expressed (FDR-adjusted P-value < 0.05) transcript isoforms between cell types.

Gene	NCBI Gene Name	Differential Expression		
		Under^1^	Over^2^	Non^3^
Macf1	microtubule-actin crosslinking factor 1	6	10	15
Rbm5	RNA binding motif protein 5	6	9	14
Bag6	BCL2-associated athanogene 6	7	7	13
Arhgef1	Rho guanine nucleotide exchange factor (GEF) 1	7	7	11
Rbm39	RNA binding motif protein 39	6	7	12
Gapvd1	GTPase activating protein and VPS9 domains 1	7	5	11
Josd2	Josephin domain containing 2	4	7	10
Lrrc16a	leucine rich repeat containing 16A	6	5	9
Rnf220	ring finger protein 220	2	8	9
Gripap1	GRIP1 associated protein 1	6	4	9
Anapc1	anaphase promoting complex subunit 1	5	5	9
Arhgap4	Rho GTPase activating protein 4	2	8	9
Hnrnpl	heterogeneous nuclear ribonucleoprotein L	4	6	9
Il15ra	interleukin 15 receptor, alpha chain	5	5	9
Srsf5	serine/arginine-rich splicing factor 5	3	7	8
Ythdc1	YTH domain containing 1	3	6	8
Brwd1	bromodomain and WD repeat domain containing 1	4	5	8
Rtn4	reticulon 4	4	5	8
Sfrs18	PNN interacting serine/arginine-rich	4	5	8
Wdr13	WD repeat domain 13	5	4	8
Wnk1	WNK lysine deficient protein kinase 1	5	4	7
Ctage5	CTAGE family, member 5	3	6	6
Hivep3	human immunodeficiency virus type I enhancer binding protein 3	4	5	5

^1^Over: transcript isoforms over-expressed in microglia.

^2^Under: transcript isoforms under-expressed in microglia.

^3^Non: not differentially expressed transcript isoforms (FDR-adjusted P-value < 0.05).

Alternative splicing events differentiating cell types were identified in 387 genes including 1,607 transcript isoforms. Confirming our findings, at least 72 of these genes have recorded alternative splicing events in the microglia as described in the database of alternative splicing of brain cells [130]. The alternative splicing cassette characterized by the inclusion or exclusion of an exon was the most frequent type of splicing event with 68% of occurrences. Alternative 3′ and alternative 5′ splicing events characterized by alternative usage of a splicing site on the 3′ and 5′ end of an exon, respectively were the second most common and the less common events amounting to 25% and 7% of the transcript isoforms, respectively. Intron retention (the inclusion or exclusion of a segment previously annotated to be an intron); tandem cassette (the inclusion or exclusion of two or more tandem exons); and mutually exclusive exons (the inclusion of one exon in one transcript and inclusion of a different exon in another transcript) were identified in 19%, 13%, and 11% of the 387 genes investigated, respectively. More than one alternative splicing mode was observed in some genes including WNK lysine deficient protein kinase 1 (Wnk1), tripartite motif-containing 33 (Trim33), and SWI/SNF related matrix associated actin dependent regulator of chromatin subfamily c member 2 (Smarcc2). Among the genes exhibiting alternative splicing events in the microglia, several genes have been associated with neurological disorders. Schizophrenia has been associated with microtubule-actin crosslinking factor 1 (Macf1) [131,132], Wnk1 [131], and Fused in sarcoma (Fus) [133]. Our results confirm alternative splicing events that are shared between neurological disorders and microglia after immune challenge and both linked to depressive behaviors.

### Gene networks of transcriptomic differences between microglia and macrophages from BCG-challenged mice

**Fig 3:**
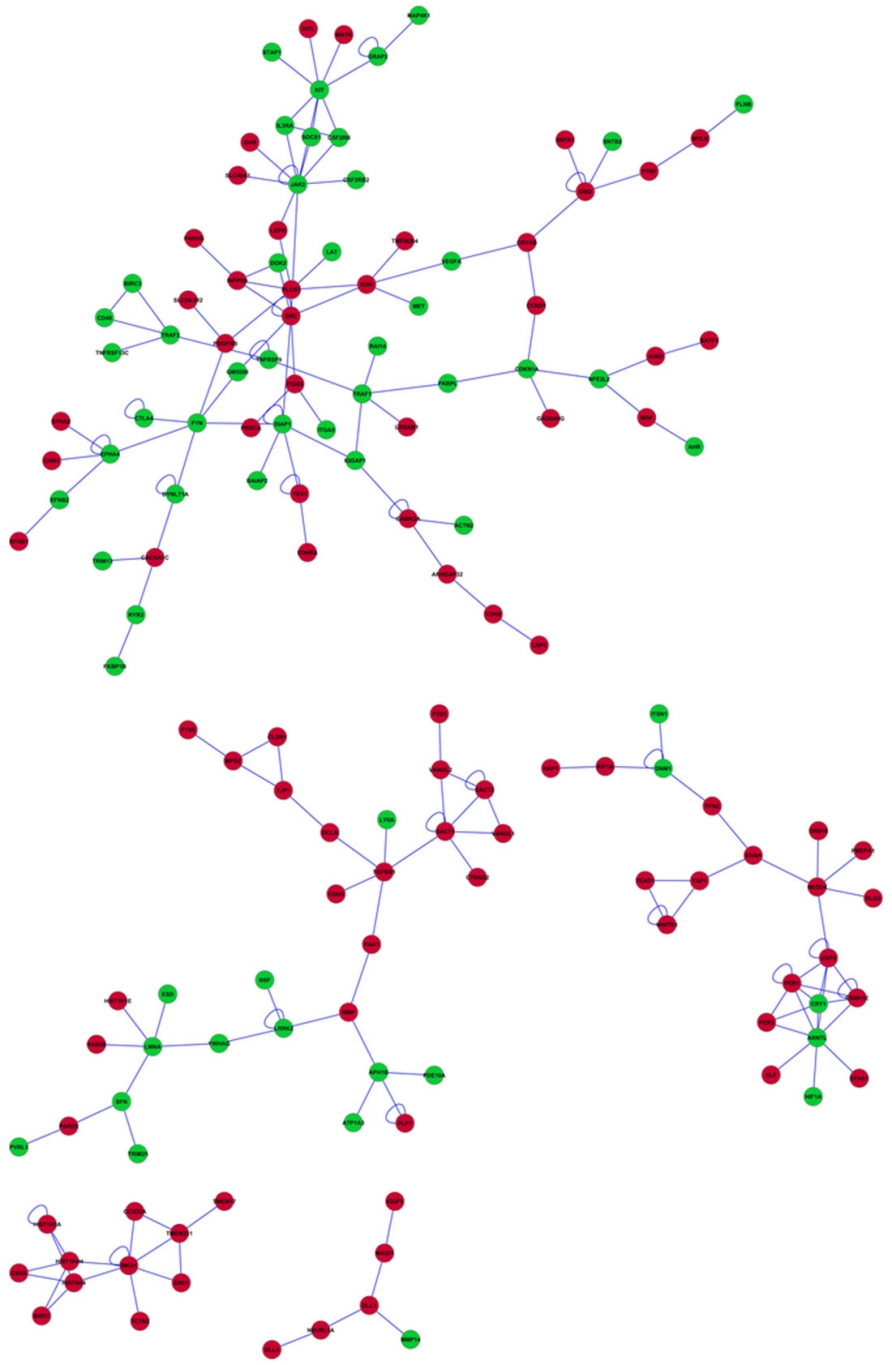
Network of genes differentially expressed between microglia and macrophages in BCG-challenged mice. Red (green) nodes denote genes over- (under-) expressed in BCG-challenged relative to Control mice. All genes were differentially expressed at FDR-adjusted P-value < 0.0001. Edges denote known relationships between genes in the SysBiomics repository. Framed genes (squares) are discussed in the manuscript.

Additional understanding of the relationship among genes in microglia and macrophages from BCG-challenged mice was gained from networks visualization. A network was depicted considering the 2,487 most extreme differentially expressed genes between microglia and macrophages (log_2_(fold change) > |2|, FDR-adjusted P-value < 2.0 x 10^−4^). From these, sub-networks connecting more than 5 genes are presented to facilitate visualization and interpretation. A gene network was constructed that included 146 genes with 39% of the genes over-expressed in microglia and the rest under-expressed (Fig 3). The predominance of genes under-expressed in microglia relative to macrophages suggests that gene dysregulation leading to negative neurological effects is more contained and quickly resolved in microglia than in macrophages after a BCG challenge.

Janus kinase (Jak2) was the most connected gene in the network, linked to 11 other genes (Fig 3). This gene was under-expressed in microglia relative to macrophages and was affiliated to the enriched categories of protein kinase activity, nucleotide binding, apoptosis, cell differentiation, and regulation of phosphorylation in four functional clusters (Table 7). Kit oncogene (Kit) was under-expressed in microglia relative to macrophages, was linked to eight other genes, and was affiliated to the enriched categories cell migration, chemotaxis, nucleotide binding, phosphorylation, protein kinase activity, apoptosis, leukocyte proliferation and differentiation distributed across 11 functional clusters (Table 7). Diaphanous homolog 1 (Diap1) was also under-expressed in microglia, was connected to seven gene nodes, and was affiliated to the functional categories actin cytoskeleton and protein organization that were enriched below the threshold.

The most connected genes over-expressed in microglia relative to macrophages (Fig 3) included Rous sarcoma oncogene (Src), ubiquitin specific peptidase 2 (Usp2), and transforming growth factor, beta receptor I (Tgfbr1) and these genes were linked to 9, 7, and 5 other genes, respectively. Src is affiliated to enriched categories including cell migration, protein kinase activity, nucleotide binding, apoptosis, and cell adhesion in six clusters (Table 7). Ups2 is affiliated to enriched categories including phosphate and phosphorus metabolic process in one cluster and Tgfbr1 is affiliated to enriched categories including cell migration, nucleotide binding, phosphorylation, protein kinase activity, angiogenesis and apoptosis in seven clusters (Table 7). The genes most connected in the network enriched apoptosis, cell migration, chemotaxis, and cell adhesion categories. This finding is in agreement with reports of the functional response of macrophages [124,134] and microglia [135] after a bacterial challenge.

This research studied the transcriptome of whole-brain microglia. However, microglia from different brain regions is likely to contribute unequally to depressive behaviors. Likewise, some brain regions are more responsive to periphery immune challenges than others. Additional studies that can parse the differential effect of periphery BCG challenge across brain regions and the corresponding changes in the transcriptome will offer more precise insights into the association between the transcriptome changes in response to immune challenge within brain regions and corresponding depressive-like behaviors.

## Conclusions

The impact of immune challenge on the microglia after recovery from sickness and the implications on depressive behaviors was studied. The transcriptome of microglia 7 days after BCG challenge was compared to the corresponding transcriptome from unchallenged Control mice and to macrophages from the same mice. The number of genes differentially expressed between BCG-challenged and Control mice suggests the capacity of microglia to restrain or quickly resolve transcriptomic dysregulation relative to macrophages. The differential expression of Kmo in microglia between BCG-challenged and Control mice suggests that Kmo is a potential target of pro-inflammatory cytokines in the kynurenine pathway and a potential factor of depressive-like symptoms that remain after sickness symptoms subside. The over-expression of a number of genes, including Ifng and Il1b, in microglia relative to macrophages offered further evidence of transcriptome conditions associated with depression-like symptoms. Functional analysis highlighted the enrichment of categories including immune response and chemotaxis by genes over-expressed in BCG-challenged compared to Control mice in both cell types. Network visualization uncovered the key role of Jak2, Stat3, Stat1, and Nfe2 as hub genes, dysregulated and connected to other dysregulated genes in the microglia 7 days after BCG challenge.

The large number of differentially expressed genes between cell types from BCG-challenged mice speaks to the response of these cells to immune challenge. Also, a number of genes exhibiting differential splicing events between microglia and macrophages in this study have been linked to neurological disorders. Network visualization depicted the capability of microglia to exhibit transcriptome dysregulation after sickness recovery from immune challenge, albeit lower than macrophages. The present study provides ample evidence that the microglia transcriptome dysregulation after BCG challenge is shared with neurological disorders that also exhibit depressive behaviors.

## Supporting Information

S1 Table Differentially expressed genes (FDR-adjusted P-value < 0.05) between BCG-challenged and Control mice within cell type and supporting literature review. (DOCX)

S2 Table Functional cluster (DAVID Enrichment score ES > 2) of categories enriched among differentially expressed genes between BCG-challenged and Control mice within cell type. (DOCX)

S3 Table Gene Set Enrichment Analysis (GSEA) categories enriched among transcript isoforms over-expressed (FDR-adjusted P-value < 0.05 and > 10 transcript isoforms) and under- expressed (Nominal P-value < 0.05 > 10 transcript isoforms) in BCG-challenged relative to Control mice in microglia. (DOCX)

S4 Table Gene Set Enrichment Analysis (GSEA) categories enriched among transcript isoforms over-expressed (FDR-adjusted P-value < 0.05 and > 10 transcript isoforms) and under- expressed (FDR-adjusted P-value < 0.05 > 10 transcript isoforms) in BCG-challenged relative to Control in macrophages. (DOCX)

S5 Table Differentially expressed genes (FDR-adjusted P-value < 2.0 x 10^−4^) between microglia and macrophages in BCG-challenged mice and supporting literature review. (DOCX)

S6 Table Functional cluster (DAVID Enrichment score ES > 2) of categories enriched among differentially expressed transcript isoforms between microglia and macrophages in BCG-challenged mice. (DOCX)

S7 Table Gene Set Enrichment Analysis (GSEA) categories enriched among transcript isoforms under-expressed (FDR-adjusted P-value < 0.05 and > 10 transcript isoforms) and over- expressed (P-value < 0.01 and > 10 transcript isoforms) in microglia relative to peripheral macrophages in BCG-challenged mice. (DOCX)

S8 Table Genes exhibiting an alternative splicing event between microglia and peripheral macrophages in BCG-challenged mice including at least two transcript isoforms and at least one over- or under-expressed (FDR-adjusted P-value < 0.05) transcript isoforms between cell types. (DOCX)

S9 Table Functional cluster (DAVID Enrichment score ES > 2) of categories enriched by transcript isoforms expressed solely in microglia cells in BCG-challenged mice. (DOCX)
