# Supplementary material for: Microglia Transcriptome Changes in a Model of Depressive Behavior after Immune Challenge
Source: PLoS One. 2016 Mar 9;11(3):e0150858. doi: 10.1371/journal.pone.0150858 (PMC4784788; doi:10.1371/journal.pone.0150858)
Supplement: S1 Table — (DOCX) [file pone.0150858.s001.docx]

S1 Table. Differentially expressed genes (FDR-adjusted P-value < 0.05) between BCG-challenged and Control mice within cell type and supporting literature review.

| Gene Symbol | NCBI Gene Name | | Log_2_(BCG/ Control) | References |
| --- | --- | --- | --- | --- |
| Microglia from BCG-challenged vs. Control Mice | | | | |
| Saa3 | | serum amyloid A 3 | 5.90 | Sohn et al. (2012); Smith et al.(2008); Szulzewsky et al. (2015); Hickman et al. (2013); Gautier et al. (2012) |
| Cadm3 | | cell adhesion molecule 3 | 5.14 | Smith et al.(2008) |
| Car6 | | carbonic anhydrase 6 | 5.01 | Suga et al. (2014); Szulzewsky et al. (2015); Gautier et al. (2012) |
| Steap4 | | STEAP family member 4 | 4.89 | Liu et al. (2013); Ebert et al. (2012) |
| Sele | | selectin E | 4.80 | Mabbott et al. (2011); Israelsson et al. (2008) |
| Cxcr1 | | chemokine (C-X-C Motif) receptor 1 | 4.03 | Krause et al. (2014); Li et al. (2006); Hickman et al. (2013) |
| Ifitm1 | | interferon induced transmembrane protein 1 | 3.72 | Hwang et al. (2013); Israelsson et al. (2008); Szulzewsky et al. (2015); Hickman et al. (2013) |
| Irg1 | | immunoresponsive 1 homolog: involved in the inhibition of the inflammatory response. | 3.66 | Li et al. (2006); Takasaki et al. (2012); Martinez et al. (2013) |
| Lrg1 | | leucine-rich alpha-2-glycoprotein 1 | 3.57 | Yamashita et al. (2013); Gautier et al. (2012) |
| Prok2 | | prokineticin 2 | 3.48 | Yamashita et al.(2013) |
| Cldn4 | | claudin 4 | 3.44 | Danaher et al. (2008) |
| Cfb | | complement factor B | 3.39 | Sohn et al. (2012);  Szulzewsky et al. (2015) |
| Slfn4 | | schlafen 4 | 3.33 | Takasaki et al. (2012); Gautier et al. (2012) |
| Nxpe5 | | neurexophilin and PC-esterase domain family, member 5 | 3.29 | Brodziak et al. (2013) |
| Ly6i | | lymphocyte antigen 6 complex, locus I | 3.29 | Martinez et al. (2013); Szulzewsky et al. (2015) |
| Oas3 | | 2'-5'-oligoadenylate synthetase 3, 100kDa | 3.27 | Chen et al. (2010);  Szulzewsky et al. (2015) |
| Ifi205 | | interferon activated gene 205 | 3.15 | Suga et al. (2014);  Szulzewsky et al. (2015) |
| Stfa1 | | stefin A1 | 3.12 | Lazić et al. (2011) |
| Plac8 | | placenta-specific 8 | 3.09 | Israelsson et al. (2008); Szulzewsky et al. (2015) |
| Tarm1 | | T cell-interacting, activating receptor on myeloid cells 1 | 2.99 | Dasgupta et al. (2013) |
| F10 | | coagulation factor X | 2.98 | Choi et al. (2009);  Gautier et al. (2012) |
| Serpina3f | | serine (or cysteine) peptidase inhibitor, clade A, member 3F | 2.97 | Sohn et al. (2012);  Gautier et al. (2012); Szulzewsky et al. (2015) |
| Asprv1 | | aspartic peptidase, retroviral-like 1 | 2.94 | Nixon et al. (2015) |
| Gbp2 | | guanylate binding protein 2 | 2.87 | Israelsson et al. (2008); Chen et al. (2010); Sohn et al. (2012) |
| Vcan | | versican | 2.87 | Smith et al. (2005) |
| BC100530 | | cDNA sequence BC100530 | 2.84 |  |
| Mefv | | mediterranean fever | 2.81 | Agyemang et al. (2015) |
| Foxd4 | | forkhead box D4 | 2.80 | Zimmer et al. (2011) |
| Rhov | | ras homolog gene family, member V | 2.79 | Xia et al. (2014) |
| 4933412O06Rik | | RIKEN cDNA 4933412O06 gene | 2.73 |  |
| Fcgr4 | | Fc receptor, IgG, low affinity IV | 1.76 | Fuller et al.(2014) |
| S100a9 | | S100 calcium binding protein A9 (calgranulin B) | 1.49 | Lisowski et al. (2012) |
| S100a8 | | S100 calcium binding protein A8 (calgranulin A) | 1.49 | Lisowski et al. (2012);  Pan et al.(2012) |
| Il1b | | interleukin 1 beta | 1.60 | O'Connor et al. (2009);  Norma et al. (2010) |
| Ptgs2 | | prostaglandin-endoperoxide synthase 2 | 1.44 | Kawano et al. (2006);  Norden et al. (2013) |
| Il1r1 | | interleukin 1 receptor, type I | 1.04 | Day (2000) |
| Kmo | | kynurenine 3-monooxygenase | 1.02 | Parrott and O'Connor (2015) |
| Il4ra | | interleukin 4 receptor, alpha | 0.55 | Chen et al. (2012) |
| Fcgr2b | | Fc receptor, IgG, low affinity IIb | 0.42 | Fuller et al. (2014);  Hickman et al. (2013) |
| Aplp1 | | amyloid beta (A4) precursor-like protein 1 | -0.52 | Bayer et al. (1999) |
| Ermap,Zfp691 | | erythroblast membrane-associated protein | -0.53 | Duke et al. (2007) |
| Sox4 | | SRY (sex determining region Y)-box 4 | -0.57 | Butovsky et al. (2014) |
| Ccr6 | | chemokine (C-C motif) receptor 6 | -0.57 | Li et al. (2006);  Hickman et al. (2013) |
| Gp9 | | glycoprotein 9 | -0.58 | Szulzewsky et al. (2015) |
| Eps8l1 | | EPS8-like 1 | -0.60 | Tocchetti et al. (2013) |
| Gpr165 | | G protein-coupled receptor 165 | -0.60 | Szulzewsky et al. (2015); Gautier et al. (2012) |
| Gm10406 | | predicted gene 10406 | -0.61 | Szulzewsky et al. (2015) |
| Hbb-b1 | | hemoglobin, beta adult major chain | -0.64 | Smith et al.(2008);  Chen et al. (2010) |
| Khdrbs3 | | KH domain containing, RNA binding, signal transduction associated 3 | -0.66 | Butovsky et al. (2014) |
| Upk1b | | uroplakin 1B | -0.69 | Hickman et al. (2013);  Gautier et al. (2012) |
| Beta-s | | hemoglobin, beta adult s chain | -0.71 |  |
| Tmem88b | | transmembrane protein 88B | -0.76 | Fleiss et al. (2015) |
| Gpr37 | | G protein-coupled receptor 37 | -0.77 | Ebert et al. (2012);  Szulzewsky et al. (2015) |
| Cldn11 | | claudin 11 | -0.77 | Smith et al.(2008) |
| Plekhb1 | | pleckstrin homology domain containing, family B (evectins) member 1 | -0.77 | Lee et al. (2012) |
| Klhdc8b | | kelch domain containing 8B | -0.81 | Samal et al. (2013) |
| S100b | | S100 protein, beta polypeptide, neura | -0.81 | Smith et al.(2008) |
| Sec16b | | SEC16 homolog B (S. cerevisiae) | -0.82 | Yamaguchi et al. (2007) |
| Sema4b | | sema domain, immunoglobulin domain (Ig), transmembrane domain (TM) and short cytoplasmic domain, (semaphorin) 4B | -0.84 | Giraudon et al. (2005) |
| Olig1 | | oligodendrocyte transcription factor 1 | -0.84 | Glezer et al. (2006) |
| D7Ertd443e | | DNA segment, Chr 7, ERATO Doi 443, expressed | -0.87 | Jacob (2011) |
| Tnfrsf17 | | tumor necrosis factor receptor superfamily, member 17 | -0.88 | Saltzman et al. (2013); Hickman et al. (2013) |
| Mobp | | myelin-associated oligodendrocytic basic protein | -0.95 | Smith et al. (2008);  Solga et al. (2015) |
| Bcas1 | | breast carcinoma amplified sequence 1 | -0.96 | Satoh and Kino (2015) |
| Retnla | | resistin like alpha | -1.00 | Hickman et al. (2013);  Gautier et al. (2012) |
| Hba-a1 | | hemoglobin alpha, adult chain 1 | -1.09 | Smith et al.(2008); Mabbott et al. (2011) |
| Hba-a2 | | hemoglobin alpha, adult chain 2 | -1.10 | Smith et al.(2008); Mabbott et al. (2011) |
| Tcap | | titin-cap | -1.15 | Casey et al. (2015) |
| Ccdc162 | | coiled-coil domain containing 162 | -1.21 | Zhang et al. (2014) |
| Macrophages from BCG-challenged vs Control Mice | | | | |
| S100a9 | S100 calcium binding protein A9 (calgranulin B) | | 10.11 | Gebhardt et al. (2006) |
| Mrgpra2a | MAS-related GPR, member A2A | | 10.10 |  |
| Stfa2l1 | stefin A2 like 1 | | 10.03 |  |
| Ly6i | lymphocyte antigen 6 complex, locus I | | 9.42 |  |
| Asprv1 | aspartic peptidase, retroviral-like 1 | | 9.15 |  |
| Gm5483 | predicted gene 5483 | | 9.12 |  |
| 1100001G20Rik | WAP four-disulfide core domain 21 | | 8.87 |  |
| Ly6c2 | lymphocyte antigen 6 complex, locus C2 | | 7.92 | Stier and Spindler (2012) |
| Nos2 | nitric oxide synthase 2, inducible | | 7.69 | Shiloh et al.(1999) |
| Il1f9 | interleukin 1 family, member 9 | | 7.66 | Foster et al. (2014) |
| Ccl8 | chemokine (C-C motif) ligand 8 | | 7.45 | Liu et al.(2013) |
| 2010002M12Rik | interferon induced protein with tetratricopeptide repeats 1B like 2 | | 7.32 |  |
| Spon1 | spondin 1, (f-spondin) extracellular matrix protein | | 7.23 | Palmer et al. (2014) |
| S100a8 | S100 calcium binding protein A8 (calgranulin A) | | 7.15 | Okada et al. (2015) |
| Entpd3 | ectonucleoside triphosphate diphosphohydrolase 3 | | 7.03 |  |
| Cxcr2 | chemokine (C-X-C motif) receptor 2 | | 7.02 |  |
| A530046M15Rik | RIKEN cDNA A530046M15 gene | | 6.96 |  |
| Ifng | interferon gamma | | 6.45 | MacMicking et al. (1997);  Wang et al. (1999) |
| Cxcl9 | chemokine (C-X-C motif) ligand 9 | | 6.40 |  |
| Col5a3 | collagen, type V, alpha 3 | | 6.27 |  |
| C1s | complement component 1, s subcomponent 1 | | 6.24 |  |
| Gpr141 | G protein-coupled receptor 141 | | 6.15 |  |
| 2010005H15Rik | RIKEN cDNA 2010005H15 gene | | 6.14 |  |
| Iigp1 | interferon inducible GTPase 1 | | 6.08 |  |
| Clrn3 | clarin 3 | | 6.07 |  |
| Plekhg4 | pleckstrin homology domain containing, family G (with RhoGef domain) member 4 | | 5.95 |  |
| Ltf | lactotransferrin | | 5.70 |  |
| Amer2 | APC membrane recruitment 2 | | 5.69 |  |
| Gm9733 | predicted gene 9733 | | 5.69 |  |
| Flt1 | FMS-like tyrosine kinase 1 | | 5.67 |  |
| Il1b | Interleukin 1 beta | | 1.97 | Jayaraman et al. (2013) |
| Kmo | kynurenine 3-monooxygenase | | 0.77 | Chiarugi et al. (2001) |
| Abca6 | ATP-binding cassette, sub-family A (ABC1), member 6 | | -2.30 |  |
| Tox2 | TOX high mobility group box family member 2 | | -2.31 |  |
| Vsig4 | V-set and immunoglobulin domain containing 4 | | -2.32 |  |
| Cyp11a1 | cytochrome P450, family 11, subfamily a, polypeptide 1 | | -2.33 |  |
| Mlph | melanophilin | | -2.33 |  |
| Apoc4 | apolipoprotein C-IV | | -2.35 |  |
| Ednra | endothelin receptor type A | | -2.36 |  |
| Neurod4 | neurogenic differentiation 4 | | -2.39 |  |
| 1810011O10Rik | RIKEN cDNA 1810011O10 gene | | -2.42 |  |
| Try5 | trypsin 5 | | -2.43 |  |
| Artn | artemin | | -2.49 |  |
| Kcne3 | potassium voltage-gated channel, Isk-related subfamily, gene 3 | | -2.50 |  |
| Gna14 | guanine nucleotide binding protein, alpha 14 | | -2.53 |  |
| Ccl24 | chemokine (C-C motif) ligand 24 | | -2.53 |  |
| Tpsab1 | tryptase alpha/beta 1 | | -2.54 |  |
| Pf4 | platelet factor 4 | | -2.56 |  |
| Frmpd1 | FERM and PDZ domain containing 1 | | -2.57 |  |
| Fcrls | Fc receptor-like S, scavenger receptor | | -2.60 | Strait et al.(2015) |
| 1810046K07Rik | RIKEN cDNA 1810046K07 gene | | -2.61 |  |
| Cd209a | CD209a antigen | | -2.66 | Lu et al. (2013) |
| Klf15 | Kruppel-like factor 15 | | -2.68 |  |
| A4galt | alpha 1,4-galactosyltransferase | | -2.70 |  |
| Cd209f | CD209f antigen | | -2.73 |  |
| Cd209b | CD209b antigen | | -2.76 |  |
| Cyp26a1 | cytochrome P450, family 26, subfamily a, polypeptide 1 | | -2.84 |  |
| Retnla | resistin like alpha | | -2.89 |  |
| Sox7 | SRY (sex determining region Y)-box 7 | | -3.01 |  |
| Rprm | reprimo, TP53 dependent G2 arrest mediator candidate | | -3.07 | Ooki et al. (2013) |
| Mucl1 | mucin-like 1 | | -3.09 |  |
| Prss37 | protease, serine 37 | | -3.49 |  |
