# Supplementary material for: Microglia Transcriptome Changes in a Model of Depressive Behavior after Immune Challenge
Source: PLoS One. 2016 Mar 9;11(3):e0150858. doi: 10.1371/journal.pone.0150858 (PMC4784788; doi:10.1371/journal.pone.0150858)
Supplement: S2 Table — (DOCX) [file pone.0150858.s002.docx]

S2 Table. Functional cluster (DAVID Enrichment score ES > 2) of categories enriched among differentially expressed genes between BCG-challenged and Control mice within cell type.

| Cell type and Category^1^ | Terms^2^ | Count | P-value | ES |
| --- | --- | --- | --- | --- |
| Microglia (562 genes) | | | | |
| Cluster 1 |  |  |  |  |
| GO_BP_FAT | GO:0006952~defense response | 57 | 7.96E-21 | 12.96 |
| GO_BP_FAT | GO:0006954~inflammatory response | 29 | 8.04E-11 |  |
| GO_BP_FAT | GO:0009611~response to wounding | 34 | 2.06E-09 |  |
|  |  |  |  |  |
| Cluster 2 |  |  |  |  |
| GO_BP_FAT | GO:0006935~chemotaxis | 18 | 1.52E-08 | 5.63 |
| GO_BP_FAT | GO:0042330~taxis | 18 | 1.52E-08 |  |
| GO_BP_FAT | GO:0007626~locomotory behavior | 20 | 7.25E-05 |  |
| GO_BP_FAT | GO:0007610~behavior | 24 | 1.73E-03 |  |
|  |  |  |  |  |
| Cluster 3 |  |  |  |  |
| GO_MF_FAT | GO:0030246~carbohydrate binding | 33 | 4.43E-10 | 5.12 |
| GO_MF_FAT | GO:0030247~polysaccharide binding | 15 | 1.59E-05 |  |
| GO_MF_FAT | GO:0001871~pattern binding | 15 | 1.59E-05 |  |
| GO_MF_FAT | GO:0005539~glycosaminoglycan binding | 13 | 9.32E-05 |  |
| GO_MF_FAT | GO:0008201~heparin binding | 9 | 2.41E-03 |  |
|  |  |  |  |  |
| Cluster 4 |  |  |  |  |
| GO_MF_FAT | GO:0030414~peptidase inhibitor activity | 20 | 6.06E-07 | 4.88 |
| GO_MF_FAT | GO:0004866~endopeptidase inhibitor activity | 19 | 7.16E-07 |  |
| GO_MF_FAT | GO:0004857~enzyme inhibitor activity | 23 | 1.64E-06 |  |
| GO_MF_FAT | GO:0004869~cysteine-type endopeptidase inhibitor activity | 8 | 9.83E-05 |  |
| GO_MF_FAT | GO:0004867~serine-type endopeptidase inhibitor activity | 10 | 5.42E-03 |  |
|  |  |  |  |  |
| Cluster 5 |  |  |  |  |
| GO_BP_FAT | GO:0002252~immune effector process | 20 | 3.82E-09 | 4.75 |
| GO_BP_FAT | GO:0002250~adaptive immune response | 12 | 3.01E-05 |  |
| GO_BP_FAT | GO:0002460~adaptive immune response based on somatic recombination of immune receptors built from immunoglobulin superfamily domains | 12 | 3.01E-05 |  |
| GO_BP_FAT | GO:0002443~leukocyte mediated immunity | 12 | 5.20E-05 |  |
| GO_BP_FAT | GO:0002449~lymphocyte mediated immunity | 11 | 6.63E-05 |  |
| GO_BP_FAT | GO:0019724~B cell mediated immunity | 10 | 1.02E-04 |  |
| GO_BP_FAT | GO:0016064~immunoglobulin mediated immune response | 9 | 4.54E-04 |  |
|  |  |  |  |  |
| Cluster 6 |  |  |  |  |
| GO_BP_FAT | GO:0007155~cell adhesion | 38 | 2.86E-06 | 4.37 |
| GO_BP_FAT | GO:0022610~biological adhesion | 38 | 2.97E-06 |  |
| GO_BP_FAT | GO:0016337~cell-cell adhesion | 15 | 9.23E-03 |  |
|  |  |  |  |  |
| Cluster 7 |  |  |  |  |
| GO_BP_FAT | GO:0009617~response to bacterium | 17 | 1.35E-05 | 3.92 |
| GO_BP_FAT | GO:0042742~defense response to bacterium | 12 | 3.00E-04 |  |
| GO_BP_FAT | GO:0050830~defense response to Gram-positive bacterium | 6 | 4.42E-04 |  |
|  |  |  |  |  |
| Cluster 8 |  |  |  |  |
| GO_MF_FAT | GO:0008009~chemokine activity | 7 | 6.41E-04 | 3.02 |
| GO_MF_FAT | GO:0042379~chemokine receptor binding | 7 | 7.40E-04 |  |
| GO_MF_FAT | GO:0005125~cytokine activity | 14 | 1.88E-03 |  |
|  |  |  |  |  |
| Cluster 9 |  |  |  |  |
| GO_BP_FAT | GO:0045321~leukocyte activation | 20 | 2.19E-05 | 3.00 |
| GO_BP_FAT | GO:0001775~cell activation | 21 | 3.40E-05 |  |
| GO_BP_FAT | GO:0046649~lymphocyte activation | 17 | 1.46E-04 |  |
| GO_BP_FAT | GO:0030097~hemopoiesis | 19 | 4.00E-04 |  |
| GO_BP_FAT | GO:0048534~hemopoietic or lymphoid organ development | 20 | 5.77E-04 |  |
| GO_BP_FAT | GO:0002520~immune system development | 20 | 1.03E-03 |  |
| GO_BP_FAT | GO:0042110~T cell activation | 11 | 2.03E-03 |  |
| GO_BP_FAT | GO:0030098~lymphocyte differentiation | 10 | 5.99E-03 |  |
| GO_BP_FAT | GO:0002521~leukocyte differentiation | 11 | 8.53E-03 |  |
| GO_BP_FAT | GO:0030183~B cell differentiation | 6 | 1.04E-02 |  |
| GO_BP_FAT | GO:0042113~B cell activation | 6 | 7.65E-02 |  |
|  |  |  |  |  |
| Cluster 10 |  |  |  |  |
| GO_BP_FAT | GO:0048002~antigen processing and presentation of peptide antigen | 9 | 5.62E-06 | 2.48 |
| GO_BP_FAT | GO:0019886~antigen processing and presentation of exogenous peptide antigen via MHC class II | 6 | 6.78E-05 |  |
| GO_BP_FAT | GO:0002495~antigen processing and presentation of peptide antigen via MHC class II | 6 | 6.78E-05 |  |
| GO_BP_FAT | GO:0002504~antigen processing and presentation of peptide or polysaccharide antigen via MHC class II | 6 | 1.68E-04 |  |
| GO_BP_FAT | GO:0002478~antigen processing and presentation of exogenous peptide antigen | 6 | 4.42E-04 |  |
| KEGG_PATHWAY | mmu05332:Graft-versus-host disease | 9 | 9.53E-04 |  |
| GO_BP_FAT | GO:0019884~antigen processing and presentation of exogenous antigen | 6 | 1.15E-03 |  |
| KEGG_PATHWAY | mmu04940:Type I diabetes mellitus | 9 | 1.65E-03 |  |
| GO_BP_FAT | GO:0019882~antigen processing and presentation | 9 | 3.75E-03 |  |
| KEGG_PATHWAY | mmu05330:Allograft rejection | 8 | 4.29E-03 |  |
| KEGG_PATHWAY | mmu04612:Antigen processing and presentation | 10 | 4.93E-03 |  |
| KEGG_PATHWAY | mmu05416:Viral myocarditis | 10 | 6.10E-03 |  |
| KEGG_PATHWAY | mmu05320:Autoimmune thyroid disease | 8 | 1.38E-02 |  |
| GO_MF_FAT | GO:0042605~peptide antigen binding | 3 | 5.83E-02 |  |
| KEGG_PATHWAY | mmu05322:Systemic lupus erythematosus | 8 | 7.45E-02 |  |
| GO_BP_FAT | GO:0002474~antigen processing and presentation of peptide antigen via MHC class I | 3 | 8.60E-02 |  |
| KEGG_PATHWAY | mmu05310:Asthma | 3 | 3.31E-01 |  |
| GO_MF_FAT | GO:0003823~antigen binding | 3 | 6.67E-01 |  |
|  |  |  |  |  |
| Cluster 11 |  |  |  |  |
| GO_MF_FAT | GO:0019956~chemokine binding | 7 | 3.36E-05 | 2.44 |
| GO_MF_FAT | GO:0004950~chemokine receptor activity | 6 | 3.16E-04 |  |
| GO_MF_FAT | GO:0042277~peptide binding | 13 | 2.03E-03 |  |
| GO_MF_FAT | GO:0019957~C-C chemokine binding | 4 | 1.15E-02 |  |
| GO_MF_FAT | GO:0016493~C-C chemokine receptor activity | 4 | 1.15E-02 |  |
| GO_MF_FAT | GO:0008528~peptide receptor activity, G-protein coupled | 9 | 1.30E-02 |  |
| GO_MF_FAT | GO:0001653~peptide receptor activity | 9 | 1.30E-02 |  |
| GO_MF_FAT | GO:0019958~C-X-C chemokine binding | 3 | 1.53E-02 |  |
| GO_MF_FAT | GO:0016494~C-X-C chemokine receptor activity | 3 | 1.53E-02 |  |
|  |  |  |  |  |
| Cluster 12 |  |  |  |  |
| GO_BP_FAT | GO:0001817~regulation of cytokine production | 16 | 1.26E-05 |  |
| GO_BP_FAT | GO:0032649~regulation of interferon-gamma production | 6 | 2.13E-03 |  |
| GO_BP_FAT | GO:0001819~positive regulation of cytokine production | 7 | 9.06E-03 |  |
| GO_BP_FAT | GO:0051240~positive regulation of multicellular organismal process | 10 | 4.86E-02 |  |
| GO_BP_FAT | GO:0032729~positive regulation of interferon-gamma production | 3 | 8.60E-02 |  |
|  |  |  |  |  |
| Cluster 13 |  |  |  |  |
| GO_BP_FAT | GO:0002684~positive regulation of immune system process | 24 | 3.28E-08 | 2.35 |
| GO_BP_FAT | GO:0002683~negative regulation of immune system process | 12 | 1.14E-05 |  |
| GO_BP_FAT | GO:0051250~negative regulation of lymphocyte activation | 10 | 1.65E-05 |  |
| GO_BP_FAT | GO:0051249~regulation of lymphocyte activation | 16 | 1.92E-05 |  |
| GO_BP_FAT | GO:0050866~negative regulation of cell activation | 10 | 1.93E-05 |  |
| GO_BP_FAT | GO:0002695~negative regulation of leukocyte activation | 10 | 1.93E-05 |  |
| GO_BP_FAT | GO:0050868~negative regulation of T cell activation | 9 | 2.36E-05 |  |
| GO_BP_FAT | GO:0002822~regulation of adaptive immune response based on somatic recombination of immune receptors built from immunoglobulin superfamily domains | 10 | 3.06E-05 |  |
| GO_BP_FAT | GO:0002819~regulation of adaptive immune response | 10 | 3.06E-05 |  |
| GO_BP_FAT | GO:0002694~regulation of leukocyte activation | 16 | 4.26E-05 |  |
| GO_BP_FAT | GO:0050865~regulation of cell activation | 16 | 4.95E-05 |  |
| GO_BP_FAT | GO:0050863~regulation of T cell activation | 13 | 6.94E-05 |  |
| GO_BP_FAT | GO:0002706~regulation of lymphocyte mediated immunity | 10 | 1.02E-04 |  |
| GO_BP_FAT | GO:0002703~regulation of leukocyte mediated immunity | 10 | 1.83E-04 |  |
| GO_BP_FAT | GO:0051251~positive regulation of lymphocyte activation | 11 | 3.95E-04 |  |
| GO_BP_FAT | GO:0050670~regulation of lymphocyte proliferation | 10 | 5.07E-04 |  |
| GO_BP_FAT | GO:0032944~regulation of mononuclear cell proliferation | 10 | 5.07E-04 |  |
| GO_BP_FAT | GO:0007159~leukocyte adhesion | 5 | 5.59E-04 |  |
| GO_BP_FAT | GO:0002696~positive regulation of leukocyte activation | 11 | 5.99E-04 |  |
| GO_BP_FAT | GO:0070663~regulation of leukocyte proliferation | 10 | 6.09E-04 |  |
| GO_BP_FAT | GO:0050867~positive regulation of cell activation | 11 | 7.02E-04 |  |
| GO_BP_FAT | GO:0008285~negative regulation of cell proliferation | 17 | 8.64E-04 |  |
| GO_BP_FAT | GO:0002697~regulation of immune effector process | 10 | 1.02E-03 |  |
| GO_BP_FAT | GO:0002712~regulation of B cell mediated immunity | 6 | 1.58E-03 |  |
| GO_BP_FAT | GO:0002889~regulation of immunoglobulin mediated immune response | 6 | 1.58E-03 |  |
| GO_BP_FAT | GO:0030888~regulation of B cell proliferation | 6 | 2.13E-03 |  |
| GO_BP_FAT | GO:0050870~positive regulation of T cell activation | 8 | 2.96E-03 |  |
| GO_BP_FAT | GO:0050672~negative regulation of lymphocyte proliferation | 6 | 3.20E-03 |  |
| GO_BP_FAT | GO:0070664~negative regulation of leukocyte proliferation | 6 | 3.20E-03 |  |
| GO_BP_FAT | GO:0032945~negative regulation of mononuclear cell proliferation | 6 | 3.20E-03 |  |
| GO_BP_FAT | GO:0050864~regulation of B cell activation | 7 | 5.06E-03 |  |
| GO_BP_FAT | GO:0002823~negative regulation of adaptive immune response based on somatic recombination of immune receptors built from immunoglobulin superfamily domains | 4 | 5.61E-03 |  |
| GO_BP_FAT | GO:0002820~negative regulation of adaptive immune response | 4 | 5.61E-03 |  |
| GO_BP_FAT | GO:0030155~regulation of cell adhesion | 9 | 6.00E-03 |  |
| GO_BP_FAT | GO:0042129~regulation of T cell proliferation | 7 | 7.14E-03 |  |
| GO_BP_FAT | GO:0042130~negative regulation of T cell proliferation | 5 | 8.29E-03 |  |
| GO_BP_FAT | GO:0032101~regulation of response to external stimulus | 9 | 1.02E-02 |  |
| GO_BP_FAT | GO:0048585~negative regulation of response to stimulus | 7 | 1.13E-02 |  |
| GO_BP_FAT | GO:0050869~negative regulation of B cell activation | 4 | 1.22E-02 |  |
| GO_BP_FAT | GO:0042127~regulation of cell proliferation | 26 | 1.43E-02 |  |
| GO_BP_FAT | GO:0050671~positive regulation of lymphocyte proliferation | 6 | 1.47E-02 |  |
| GO_BP_FAT | GO:0032946~positive regulation of mononuclear cell proliferation | 6 | 1.47E-02 |  |
| GO_BP_FAT | GO:0002824~positive regulation of adaptive immune response based on somatic recombination of immune receptors built from immunoglobulin superfamily domains | 5 | 1.64E-02 |  |
| GO_BP_FAT | GO:0002821~positive regulation of adaptive immune response | 5 | 1.64E-02 |  |
| GO_BP_FAT | GO:0046634~regulation of alpha-beta T cell activation | 5 | 1.64E-02 |  |
| GO_BP_FAT | GO:0070665~positive regulation of leukocyte proliferation | 6 | 1.72E-02 |  |
| GO_BP_FAT | GO:0002698~negative regulation of immune effector process | 4 | 1.92E-02 |  |
| GO_BP_FAT | GO:0050727~regulation of inflammatory response | 6 | 2.46E-02 |  |
| GO_BP_FAT | GO:0045619~regulation of lymphocyte differentiation | 6 | 2.63E-02 |  |
| GO_BP_FAT | GO:0030889~negative regulation of B cell proliferation | 3 | 3.25E-02 |  |
| GO_BP_FAT | GO:0007162~negative regulation of cell adhesion | 4 | 3.49E-02 |  |
| GO_BP_FAT | GO:0050777~negative regulation of immune response | 4 | 3.86E-02 |  |
| GO_BP_FAT | GO:0045581~negative regulation of T cell differentiation | 3 | 3.90E-02 |  |
| GO_BP_FAT | GO:0046636~negative regulation of alpha-beta T cell activation | 3 | 3.90E-02 |  |
| GO_BP_FAT | GO:0045580~regulation of T cell differentiation | 5 | 5.02E-02 |  |
| GO_BP_FAT | GO:0045620~negative regulation of lymphocyte differentiation | 3 | 5.32E-02 |  |
| GO_BP_FAT | GO:0002700~regulation of production of molecular mediator of immune response | 4 | 5.55E-02 |  |
| GO_BP_FAT | GO:0002707~negative regulation of lymphocyte mediated immunity | 3 | 6.09E-02 |  |
| GO_BP_FAT | GO:0002704~negative regulation of leukocyte mediated immunity | 3 | 6.09E-02 |  |
| GO_BP_FAT | GO:0045191~regulation of isotype switching | 3 | 6.09E-02 |  |
| GO_BP_FAT | GO:0046640~regulation of alpha-beta T cell proliferation | 3 | 6.09E-02 |  |
| GO_BP_FAT | GO:0045582~positive regulation of T cell differentiation | 4 | 6.50E-02 |  |
| GO_BP_FAT | GO:0045621~positive regulation of lymphocyte differentiation | 4 | 7.52E-02 |  |
| GO_BP_FAT | GO:0042102~positive regulation of T cell proliferation | 4 | 8.06E-02 |  |
| GO_BP_FAT | GO:0050871~positive regulation of B cell activation | 4 | 9.17E-02 |  |
| GO_BP_FAT | GO:0050728~negative regulation of inflammatory response | 3 | 1.33E-01 |  |
| GO_BP_FAT | GO:0030890~positive regulation of B cell proliferation | 3 | 1.33E-01 |  |
| GO_BP_FAT | GO:0000018~regulation of DNA recombination | 3 | 1.53E-01 |  |
| GO_BP_FAT | GO:0002637~regulation of immunoglobulin production | 3 | 1.64E-01 |  |
| GO_BP_FAT | GO:0046635~positive regulation of alpha-beta T cell activation | 3 | 1.74E-01 |  |
| GO_BP_FAT | GO:0031348~negative regulation of defense response | 3 | 2.17E-01 |  |
| GO_BP_FAT | GO:0051052~regulation of DNA metabolic process | 4 | 2.38E-01 |  |
| GO_BP_FAT | GO:0032102~negative regulation of response to external stimulus | 3 | 2.82E-01 |  |
| GO_BP_FAT | GO:0008284~positive regulation of cell proliferation | 11 | 3.14E-01 |  |
| GO_BP_FAT | GO:0045596~negative regulation of cell differentiation | 7 | 4.36E-01 |  |
|  |  |  |  |  |
| Cluster 14 |  |  |  |  |
| GO_MF_FAT | GO:0005525~GTP binding | 27 | 9.21E-06 | 2.26 |
| GO_MF_FAT | GO:0019001~guanyl nucleotide binding | 27 | 1.45E-05 |  |
| GO_MF_FAT | GO:0032561~guanyl ribonucleotide binding | 27 | 1.45E-05 |  |
| GO_MF_FAT | GO:0003924~GTPase activity | 14 | 6.97E-05 |  |
| GO_MF_FAT | GO:0017076~purine nucleotide binding | 73 | 3.75E-03 |  |
| GO_MF_FAT | GO:0032555~purine ribonucleotide binding | 69 | 7.23E-03 |  |
| GO_MF_FAT | GO:0032553~ribonucleotide binding | 69 | 7.23E-03 |  |
| GO_MF_FAT | GO:0000166~nucleotide binding | 75 | 5.39E-02 |  |
| GO_MF_FAT | GO:0001882~nucleoside binding | 51 | 1.89E-01 |  |
| GO_MF_FAT | GO:0001883~purine nucleoside binding | 49 | 2.79E-01 |  |
| GO_MF_FAT | GO:0030554~adenyl nucleotide binding | 48 | 3.07E-01 |  |
| GO_MF_FAT | GO:0005524~ATP binding | 44 | 3.83E-01 |  |
| GO_MF_FAT | GO:0032559~adenyl ribonucleotide binding | 44 | 4.27E-01 |  |
|  |  |  |  |  |
| Cluster 15 |  |  |  |  |
| GO_BP_FAT | GO:0050900~leukocyte migration | 8 | 2.17E-04 | 2.23 |
| GO_BP_FAT | GO:0030595~leukocyte chemotaxis | 6 | 9.63E-04 |  |
| GO_BP_FAT | GO:0060326~cell chemotaxis | 6 | 9.63E-04 |  |
| GO_BP_FAT | GO:0030593~neutrophil chemotaxis | 4 | 1.22E-02 |  |
| GO_BP_FAT | GO:0051674~localization of cell | 16 | 1.92E-02 |  |
| GO_BP_FAT | GO:0048870~cell motility | 16 | 1.92E-02 |  |
| GO_BP_FAT | GO:0016477~cell migration | 14 | 2.38E-02 |  |
| GO_BP_FAT | GO:0006928~cell motion | 17 | 7.04E-02 |  |
|  |  |  |  |  |
| Cluster 16 |  |  |  |  |
| GO_BP_FAT | GO:0045087~innate immune response | 16 | 4.52E-07 | 2.15 |
| GO_BP_FAT | GO:0002822~regulation of adaptive immune response based on somatic recombination of immune receptors built from immunoglobulin superfamily domains | 10 | 3.06E-05 |  |
| GO_BP_FAT | GO:0002819~regulation of adaptive immune response | 10 | 3.06E-05 |  |
| GO_BP_FAT | GO:0002706~regulation of lymphocyte mediated immunity | 10 | 1.02E-04 |  |
| GO_BP_FAT | GO:0050778~positive regulation of immune response | 14 | 1.65E-04 |  |
| GO_BP_FAT | GO:0002703~regulation of leukocyte mediated immunity | 10 | 1.83E-04 |  |
| GO_BP_FAT | GO:0048584~positive regulation of response to stimulus | 16 | 3.53E-04 |  |
| GO_BP_FAT | GO:0002253~activation of immune response | 10 | 8.63E-04 |  |
| GO_BP_FAT | GO:0002697~regulation of immune effector process | 10 | 1.02E-03 |  |
| GO_BP_FAT | GO:0002712~regulation of B cell mediated immunity | 6 | 1.58E-03 |  |
| GO_BP_FAT | GO:0002889~regulation of immunoglobulin mediated immune response | 6 | 1.58E-03 |  |
| GO_BP_FAT | GO:0002708~positive regulation of lymphocyte mediated immunity | 6 | 5.77E-03 |  |
| GO_BP_FAT | GO:0002705~positive regulation of leukocyte mediated immunity | 6 | 5.77E-03 |  |
| GO_BP_FAT | GO:0002699~positive regulation of immune effector process | 6 | 1.04E-02 |  |
| GO_BP_FAT | GO:0002709~regulation of T cell mediated immunity | 4 | 1.44E-02 |  |
| GO_BP_FAT | GO:0002824~positive regulation of adaptive immune response based on somatic recombination of immune receptors built from immunoglobulin superfamily domains | 5 | 1.64E-02 |  |
| GO_BP_FAT | GO:0002821~positive regulation of adaptive immune response | 5 | 1.64E-02 |  |
| GO_BP_FAT | GO:0031349~positive regulation of defense response | 6 | 2.46E-02 |  |
| GO_BP_FAT | GO:0002711~positive regulation of T cell mediated immunity | 3 | 5.32E-02 |  |
| GO_BP_FAT | GO:0002714~positive regulation of B cell mediated immunity | 3 | 6.09E-02 |  |
| GO_BP_FAT | GO:0002891~positive regulation of immunoglobulin mediated immune response | 3 | 6.09E-02 |  |
| GO_MF_FAT | GO:0019900~kinase binding | 6 | 9.38E-02 |  |
| GO_MF_FAT | GO:0019901~protein kinase binding | 5 | 1.42E-01 |  |
| GO_BP_FAT | GO:0031343~positive regulation of cell killing | 3 | 1.43E-01 |  |
| GO_BP_FAT | GO:0001912~positive regulation of leukocyte mediated cytotoxicity | 3 | 1.43E-01 |  |
| GO_BP_FAT | GO:0010627~regulation of protein kinase cascade | 8 | 1.66E-01 |  |
| GO_BP_FAT | GO:0050730~regulation of peptidyl-tyrosine phosphorylation | 4 | 1.99E-01 |  |
| GO_BP_FAT | GO:0031341~regulation of cell killing | 3 | 2.17E-01 |  |
| GO_BP_FAT | GO:0001910~regulation of leukocyte mediated cytotoxicity | 3 | 2.17E-01 |  |
| GO_BP_FAT | GO:0043408~regulation of MAPKKK cascade | 5 | 2.85E-01 |  |
| GO_MF_FAT | GO:0019899~enzyme binding | 9 | 3.25E-01 |  |
|  |  |  |  |  |
| Cluster 17 |  |  |  |  |
| GO_MF_FAT | GO:0004896~cytokine receptor activity | 9 | 1.48E-04 | 2.07 |
| GO_MF_FAT | GO:0004908~interleukin-1 receptor activity | 3 | 1.53E-02 |  |
| GO_MF_FAT | GO:0019966~interleukin-1 binding | 3 | 1.53E-02 |  |
| GO_MF_FAT | GO:0019838~growth factor binding | 5 | 1.48E-01 |  |
|  |  |  |  |  |
| Cluster 18 |  |  |  |  |
| GO_BP_FAT | GO:0008219~cell death | 27 | 3.68E-03 | 2.05 |
| GO_BP_FAT | GO:0016265~death | 27 | 5.01E-03 |  |
| GO_BP_FAT | GO:0006915~apoptosis | 23 | 1.73E-02 |  |
| GO_BP_FAT | GO:0012501~programmed cell death | 23 | 2.06E-02 |  |
| Macrophages (FDR adjusted P-value < 0.001 and log_2_ > 0.5 (1,996 genes out of 3,851 genes) | | | | |
| Cluster 1 |  |  |  |  |
| GO_BP_FAT | GO:0006952~defense response | 104 | 9.33E-16 | 13.32 |
| GO_BP_FAT | GO:0009611~response to wounding | 85 | 2.17E-14 |  |
| GO_BP_FAT | GO:0006954~inflammatory response | 60 | 5.18E-12 |  |
|  |  |  |  |  |
| Cluster 2 |  |  |  |  |
| GO_MF_FAT | GO:0030246~carbohydrate binding | 85 | 5.64E-17 | 8.18 |
| GO_MF_FAT | GO:0030247~polysaccharide binding | 36 | 3.67E-08 |  |
| GO_MF_FAT | GO:0001871~pattern binding | 36 | 3.67E-08 |  |
| GO_MF_FAT | GO:0005539~glycosaminoglycan binding | 31 | 8.38E-07 |  |
| GO_MF_FAT | GO:0008201~heparin binding | 21 | 2.06E-04 |  |
|  |  |  |  |  |
| Cluster 3 |  |  |  |  |
| GO_BP_FAT | GO:0006935~chemotaxis | 38 | 1.57E-11 | 7.79 |
| GO_BP_FAT | GO:0042330~taxis | 38 | 1.57E-11 |  |
| GO_MF_FAT | GO:0008009~chemokine activity | 19 | 6.18E-09 |  |
| GO_MF_FAT | GO:0042379~chemokine receptor binding | 19 | 1.04E-08 |  |
| KEGG_PATHWAY | mmu04062:Chemokine signaling pathway | 51 | 4.84E-08 |  |
| GO_BP_FAT | GO:0007626~locomotory behavior | 52 | 2.41E-07 |  |
| GO_MF_FAT | GO:0005125~cytokine activity | 42 | 6.33E-07 |  |
| GO_BP_FAT | GO:0007610~behavior | 68 | 4.51E-05 |  |
|  |  |  |  |  |
| Cluster 4 |  |  |  |  |
| GO_BP_FAT | GO:0001775~cell activation | 64 | 3.03E-12 | 6.17 |
| GO_BP_FAT | GO:0045321~leukocyte activation | 59 | 5.04E-12 |  |
| GO_BP_FAT | GO:0046649~lymphocyte activation | 48 | 7.10E-09 |  |
| GO_BP_FAT | GO:0048534~hemopoietic or lymphoid organ development | 62 | 8.18E-09 |  |
| GO_BP_FAT | GO:0002520~immune system development | 64 | 9.09E-09 |  |
| GO_BP_FAT | GO:0030097~hemopoiesis | 55 | 8.06E-08 |  |
| GO_BP_FAT | GO:0002521~leukocyte differentiation | 35 | 1.64E-06 |  |
| GO_BP_FAT | GO:0042110~T cell activation | 29 | 1.13E-05 |  |
| GO_BP_FAT | GO:0030098~lymphocyte differentiation | 26 | 1.74E-04 |  |
| GO_BP_FAT | GO:0042113~B cell activation | 20 | 2.47E-04 |  |
| GO_BP_FAT | GO:0045058~T cell selection | 9 | 6.99E-04 |  |
| GO_BP_FAT | GO:0030217~T cell differentiation | 17 | 3.76E-03 |  |
| GO_BP_FAT | GO:0033077~T cell differentiation in the thymus | 10 | 3.76E-03 |  |
|  |  |  |  |  |
| Cluster 5 |  |  |  |  |
| GO_BP_FAT | GO:0001944~vasculature development | 56 | 2.73E-08 | 6.12 |
| GO_BP_FAT | GO:0001568~blood vessel development | 55 | 2.95E-08 |  |
| GO_BP_FAT | GO:0048514~blood vessel morphogenesis | 41 | 1.85E-05 |  |
| GO_BP_FAT | GO:0001525~angiogenesis | 31 | 2.31E-05 |  |
|  |  |  |  |  |
| Cluster 6 |  |  |  |  |
| GO_BP_FAT | GO:0001817~regulation of cytokine production | 41 | 7.25E-10 | 5.97 |
| GO_BP_FAT | GO:0001819~positive regulation of cytokine production | 20 | 7.71E-06 |  |
| GO_BP_FAT | GO:0051240~positive regulation of multicellular organismal process | 33 | 2.14E-04 |  |
|  |  |  |  |  |
| Cluster 7 |  |  |  |  |
| GO_BP_FAT | GO:0002684~positive regulation of immune system process | 60 | 8.35E-14 | 5.86 |
| GO_BP_FAT | GO:0048584~positive regulation of response to stimulus | 55 | 5.21E-13 |  |
| GO_BP_FAT | GO:0050778~positive regulation of immune response | 41 | 3.52E-10 |  |
| GO_BP_FAT | GO:0002697~regulation of immune effector process | 30 | 5.33E-09 |  |
| GO_BP_FAT | GO:0002703~regulation of leukocyte mediated immunity | 24 | 2.14E-07 |  |
| GO_BP_FAT | GO:0002764~immune response-regulating signal transduction | 18 | 8.79E-06 |  |
| GO_BP_FAT | GO:0002253~activation of immune response | 24 | 1.13E-05 |  |
| GO_BP_FAT | GO:0002757~immune response-activating signal transduction | 17 | 1.22E-05 |  |
| GO_BP_FAT | GO:0002768~immune response-regulating cell surface receptor signaling pathway | 16 | 1.67E-05 |  |
| GO_BP_FAT | GO:0002429~immune response-activating cell surface receptor signaling pathway | 15 | 3.12E-05 |  |
| GO_BP_FAT | GO:0050851~antigen receptor-mediated signaling pathway | 12 | 6.53E-04 |  |
| GO_BP_FAT | GO:0050853~B cell receptor signaling pathway | 7 | 1.64E-03 |  |
| GO_BP_FAT | GO:0050854~regulation of antigen receptor-mediated signaling pathway | 5 | 3.65E-02 |  |
| GO_BP_FAT | GO:0050852~T cell receptor signaling pathway | 5 | 1.81E-01 |  |
|  |  |  |  |  |
| Cluster 8 |  |  |  |  |
| GO_BP_FAT | GO:0002252~immune effector process | 36 | 2.26E-08 | 5.02 |
| GO_BP_FAT | GO:0002443~leukocyte mediated immunity | 26 | 1.80E-06 |  |
| GO_BP_FAT | GO:0002250~adaptive immune response | 25 | 2.07E-06 |  |
| GO_BP_FAT | GO:0002460~adaptive immune response based on somatic recombination of immune receptors built from immunoglobulin superfamily domains | 25 | 2.07E-06 |  |
| GO_BP_FAT | GO:0002449~lymphocyte mediated immunity | 22 | 1.57E-05 |  |
| GO_BP_FAT | GO:0019724~B cell mediated immunity | 17 | 6.57E-04 |  |
| GO_BP_FAT | GO:0016064~immunoglobulin mediated immune response | 15 | 3.90E-03 |  |
|  |  |  |  |  |
| Cluster 9 |  |  |  |  |
| GO_BP_FAT | GO:0006915~apoptosis | 78 | 1.20E-05 | 4.77 |
| GO_BP_FAT | GO:0012501~programmed cell death | 79 | 1.23E-05 |  |
| GO_BP_FAT | GO:0008219~cell death | 83 | 1.55E-05 |  |
| GO_BP_FAT | GO:0016265~death | 83 | 3.71E-05 |  |
|  |  |  |  |  |
| Cluster 10 |  |  |  |  |
| GO_BP_FAT | GO:0042325~regulation of phosphorylation | 61 | 6.86E-08 | 4.34 |
| GO_BP_FAT | GO:0051174~regulation of phosphorus metabolic process | 62 | 1.16E-07 |  |
| GO_BP_FAT | GO:0019220~regulation of phosphate metabolic process | 62 | 1.16E-07 |  |
| GO_BP_FAT | GO:0045859~regulation of protein kinase activity | 42 | 1.50E-06 |  |
| GO_BP_FAT | GO:0044093~positive regulation of molecular function | 59 | 2.35E-06 |  |
| GO_BP_FAT | GO:0043549~regulation of kinase activity | 42 | 3.51E-06 |  |
| GO_BP_FAT | GO:0051338~regulation of transferase activity | 42 | 8.90E-06 |  |
| GO_BP_FAT | GO:0043085~positive regulation of catalytic activity | 48 | 7.88E-05 |  |
| GO_BP_FAT | GO:0043405~regulation of MAP kinase activity | 22 | 7.95E-05 |  |
| GO_BP_FAT | GO:0045860~positive regulation of protein kinase activity | 28 | 1.10E-04 |  |
| GO_BP_FAT | GO:0033674~positive regulation of kinase activity | 28 | 2.52E-04 |  |
| GO_BP_FAT | GO:0051347~positive regulation of transferase activity | 28 | 4.75E-04 |  |
| GO_BP_FAT | GO:0043406~positive regulation of MAP kinase activity | 14 | 5.74E-03 |  |
| GO_BP_FAT | GO:0032147~activation of protein kinase activity | 12 | 1.25E-02 |  |
| GO_BP_FAT | GO:0000165~MAPKKK cascade | 20 | 2.18E-02 |  |
| GO_BP_FAT | GO:0000187~activation of MAPK activity | 11 | 2.77E-02 |  |
|  |  |  |  |  |
| Cluster 11 |  |  |  |  |
| GO_MF_FAT | GO:0030695~GTPase regulator activity | 74 | 7.79E-09 | 4.20 |
| GO_MF_FAT | GO:0060589~nucleoside-triphosphatase regulator activity | 74 | 1.62E-08 |  |
| GO_MF_FAT | GO:0005096~GTPase activator activity | 43 | 1.45E-06 |  |
| GO_MF_FAT | GO:0008047~enzyme activator activity | 49 | 1.12E-05 |  |
| GO_BP_FAT | GO:0051056~regulation of small GTPase mediated signal transduction | 45 | 2.40E-05 |  |
| GO_MF_FAT | GO:0005083~small GTPase regulator activity | 44 | 3.15E-05 |  |
| GO_MF_FAT | GO:0005088~Ras guanyl-nucleotide exchange factor activity | 19 | 1.57E-03 |  |
| GO_MF_FAT | GO:0005085~guanyl-nucleotide exchange factor activity | 28 | 1.87E-03 |  |
| GO_BP_FAT | GO:0046578~regulation of Ras protein signal transduction | 32 | 2.77E-03 |  |
| GO_MF_FAT | GO:0005089~Rho guanyl-nucleotide exchange factor activity | 13 | 5.55E-02 |  |
| GO_BP_FAT | GO:0035023~regulation of Rho protein signal transduction | 14 | 9.67E-02 |  |
|  |  |  |  |  |
| Cluster 12 |  |  |  |  |
| GO_BP_FAT | GO:0002684~positive regulation of immune system process | 60 | 8.35E-14 | 4.01 |
| GO_BP_FAT | GO:0002694~regulation of leukocyte activation | 48 | 2.35E-12 |  |
| GO_BP_FAT | GO:0050865~regulation of cell activation | 48 | 3.93E-12 |  |
| GO_BP_FAT | GO:0051249~regulation of lymphocyte activation | 44 | 4.47E-11 |  |
| GO_BP_FAT | GO:0050863~regulation of T cell activation | 32 | 6.25E-08 |  |
| GO_BP_FAT | GO:0002696~positive regulation of leukocyte activation | 30 | 1.02E-07 |  |
| GO_BP_FAT | GO:0050867~positive regulation of cell activation | 30 | 1.64E-07 |  |
| GO_BP_FAT | GO:0050670~regulation of lymphocyte proliferation | 26 | 1.94E-07 |  |
| GO_BP_FAT | GO:0032944~regulation of mononuclear cell proliferation | 26 | 1.94E-07 |  |
| GO_BP_FAT | GO:0070663~regulation of leukocyte proliferation | 26 | 3.29E-07 |  |
| GO_BP_FAT | GO:0050864~regulation of B cell activation | 20 | 1.02E-06 |  |
| GO_BP_FAT | GO:0051251~positive regulation of lymphocyte activation | 27 | 1.56E-06 |  |
| GO_BP_FAT | GO:0002683~negative regulation of immune system process | 23 | 4.32E-06 |  |
| GO_BP_FAT | GO:0050870~positive regulation of T cell activation | 21 | 5.41E-06 |  |
| GO_BP_FAT | GO:0045619~regulation of lymphocyte differentiation | 19 | 1.09E-05 |  |
| GO_BP_FAT | GO:0042129~regulation of T cell proliferation | 19 | 1.43E-05 |  |
| GO_BP_FAT | GO:0030888~regulation of B cell proliferation | 12 | 2.06E-04 |  |
| GO_BP_FAT | GO:0045580~regulation of T cell differentiation | 15 | 2.15E-04 |  |
| GO_BP_FAT | GO:0045061~thymic T cell selection | 8 | 3.51E-04 |  |
| GO_BP_FAT | GO:0045621~positive regulation of lymphocyte differentiation | 12 | 3.77E-04 |  |
| GO_BP_FAT | GO:0050672~negative regulation of lymphocyte proliferation | 12 | 4.99E-04 |  |
| GO_BP_FAT | GO:0032945~negative regulation of mononuclear cell proliferation | 12 | 4.99E-04 |  |
| GO_BP_FAT | GO:0070664~negative regulation of leukocyte proliferation | 12 | 4.99E-04 |  |
| GO_BP_FAT | GO:0051250~negative regulation of lymphocyte activation | 15 | 5.35E-04 |  |
| GO_BP_FAT | GO:0045577~regulation of B cell differentiation | 8 | 5.69E-04 |  |
| GO_BP_FAT | GO:0002695~negative regulation of leukocyte activation | 15 | 6.60E-04 |  |
| GO_BP_FAT | GO:0050866~negative regulation of cell activation | 15 | 6.60E-04 |  |
| GO_BP_FAT | GO:0045058~T cell selection | 9 | 6.99E-04 |  |
| GO_BP_FAT | GO:0045582~positive regulation of T cell differentiation | 11 | 9.40E-04 |  |
| GO_BP_FAT | GO:0046634~regulation of alpha-beta T cell activation | 11 | 1.58E-03 |  |
| GO_BP_FAT | GO:0030889~negative regulation of B cell proliferation | 6 | 1.79E-03 |  |
| GO_BP_FAT | GO:0046638~positive regulation of alpha-beta T cell differentiation | 8 | 1.90E-03 |  |
| GO_BP_FAT | GO:0050871~positive regulation of B cell activation | 11 | 3.17E-03 |  |
| GO_BP_FAT | GO:0046635~positive regulation of alpha-beta T cell activation | 9 | 3.37E-03 |  |
| GO_BP_FAT | GO:0030217~T cell differentiation | 17 | 3.76E-03 |  |
| GO_BP_FAT | GO:0033077~T cell differentiation in the thymus | 10 | 3.76E-03 |  |
| GO_BP_FAT | GO:0050671~positive regulation of lymphocyte proliferation | 13 | 3.77E-03 |  |
| GO_BP_FAT | GO:0032946~positive regulation of mononuclear cell proliferation | 13 | 3.77E-03 |  |
| GO_BP_FAT | GO:0046637~regulation of alpha-beta T cell differentiation | 8 | 4.91E-03 |  |
| GO_BP_FAT | GO:0050869~negative regulation of B cell activation | 7 | 5.16E-03 |  |
| GO_BP_FAT | GO:0070665~positive regulation of leukocyte proliferation | 13 | 5.29E-03 |  |
| GO_BP_FAT | GO:0045059~positive thymic T cell selection | 5 | 5.41E-03 |  |
| GO_BP_FAT | GO:0042102~positive regulation of T cell proliferation | 10 | 7.16E-03 |  |
| GO_BP_FAT | GO:0043368~positive T cell selection | 5 | 1.37E-02 |  |
| GO_BP_FAT | GO:0042130~negative regulation of T cell proliferation | 8 | 1.98E-02 |  |
| GO_BP_FAT | GO:0050868~negative regulation of T cell activation | 10 | 2.37E-02 |  |
| GO_BP_FAT | GO:0045579~positive regulation of B cell differentiation | 4 | 4.02E-02 |  |
| GO_BP_FAT | GO:0045060~negative thymic T cell selection | 4 | 5.58E-02 |  |
| GO_BP_FAT | GO:0030890~positive regulation of B cell proliferation | 6 | 6.68E-02 |  |
| GO_BP_FAT | GO:0043383~negative T cell selection | 4 | 7.39E-02 |  |
| GO_BP_FAT | GO:0043372~positive regulation of CD4-positive, alpha beta T cell differentiation | 3 | 2.33E-01 |  |
| GO_BP_FAT | GO:0043370~regulation of CD4-positive, alpha beta T cell differentiation | 3 | 3.12E-01 |  |
|  |  |  |  |  |
| Cluster 13 |  |  |  |  |
| GO_MF_FAT | GO:0032561~guanyl ribonucleotide binding | 62 | 6.52E-05 | 3.58 |
| GO_MF_FAT | GO:0019001~guanyl nucleotide binding | 62 | 6.52E-05 |  |
| GO_MF_FAT | GO:0005525~GTP binding | 60 | 1.08E-04 |  |
| GO_MF_FAT | GO:0003924~GTPase activity | 23 | 1.04E-02 |  |
|  |  |  |  |  |
| Cluster 14 |  |  |  |  |
| GO_BP_FAT | GO:0060326~cell chemotaxis | 12 | 3.36E-05 | 3.55 |
| GO_BP_FAT | GO:0030595~leukocyte chemotaxis | 12 | 3.36E-05 |  |
| GO_BP_FAT | GO:0050900~leukocyte migration | 14 | 2.43E-04 |  |
| GO_BP_FAT | GO:0030593~neutrophil chemotaxis | 6 | 2.40E-02 |  |
|  |  |  |  |  |
| Cluster 15 |  |  |  |  |
| GO_BP_FAT | GO:0030334~regulation of cell migration | 23 | 1.11E-04 | 3.53 |
| GO_BP_FAT | GO:0051270~regulation of cell motion | 25 | 1.61E-04 |  |
| GO_BP_FAT | GO:0040012~regulation of locomotion | 25 | 2.52E-04 |  |
| GO_BP_FAT | GO:0040017~positive regulation of locomotion | 14 | 3.12E-04 |  |
| GO_BP_FAT | GO:0051272~positive regulation of cell motion | 13 | 5.81E-04 |  |
| GO_BP_FAT | GO:0030335~positive regulation of cell migration | 12 | 8.45E-04 |  |
|  |  |  |  |  |
| Cluster 16 |  |  |  |  |
| GO_BP_FAT | GO:0042326~negative regulation of phosphorylation | 12 | 3.36E-05 | 3.25 |
| GO_BP_FAT | GO:0010563~negative regulation of phosphorus metabolic process | 12 | 7.36E-05 |  |
| GO_BP_FAT | GO:0045936~negative regulation of phosphate metabolic process | 12 | 7.36E-05 |  |
| GO_BP_FAT | GO:0001933~negative regulation of protein amino acid phosphorylation | 10 | 2.55E-04 |  |
| GO_BP_FAT | GO:0001932~regulation of protein amino acid phosphorylation | 26 | 4.57E-04 |  |
| GO_BP_FAT | GO:0031400~negative regulation of protein modification process | 12 | 1.08E-03 |  |
| GO_BP_FAT | GO:0032269~negative regulation of cellular protein metabolic process | 17 | 2.11E-03 |  |
| GO_BP_FAT | GO:0031399~regulation of protein modification process | 30 | 2.52E-03 |  |
| GO_BP_FAT | GO:0051248~negative regulation of protein metabolic process | 17 | 4.91E-03 |  |
| GO_BP_FAT | GO:0050732~negative regulation of peptidyl-tyrosine phosphorylation | 5 | 5.41E-03 |  |
|  |  |  |  |  |
| Cluster 17 | Enrichment Score: 3.2392318974107406 |  |  |  |
| GO_BP_FAT | GO:0002697~regulation of immune effector process | 30 | 5.33E-09 | 3.24 |
| GO_BP_FAT | GO:0002699~positive regulation of immune effector process | 20 | 3.70E-08 |  |
| GO_BP_FAT | GO:0002703~regulation of leukocyte mediated immunity | 24 | 2.14E-07 |  |
| GO_BP_FAT | GO:0002705~positive regulation of leukocyte mediated immunity | 17 | 7.49E-07 |  |
| GO_BP_FAT | GO:0002708~positive regulation of lymphocyte mediated immunity | 17 | 7.49E-07 |  |
| GO_BP_FAT | GO:0002706~regulation of lymphocyte mediated immunity | 22 | 9.85E-07 |  |
| GO_BP_FAT | GO:0001910~regulation of leukocyte mediated cytotoxicity | 14 | 2.82E-06 |  |
| GO_BP_FAT | GO:0031341~regulation of cell killing | 14 | 2.82E-06 |  |
| GO_BP_FAT | GO:0001912~positive regulation of leukocyte mediated cytotoxicity | 12 | 5.14E-06 |  |
| GO_BP_FAT | GO:0031343~positive regulation of cell killing | 12 | 5.14E-06 |  |
| GO_BP_FAT | GO:0002824~positive regulation of adaptive immune response based on somatic recombination of immune receptors built from immunoglobulin superfamily domains | 13 | 7.92E-05 |  |
| GO_BP_FAT | GO:0002821~positive regulation of adaptive immune response | 13 | 7.92E-05 |  |
| GO_BP_FAT | GO:0045088~regulation of innate immune response | 15 | 1.30E-04 |  |
| GO_BP_FAT | GO:0002822~regulation of adaptive immune response based on somatic recombination of immune receptors built from immunoglobulin superfamily domains | 16 | 3.64E-04 |  |
| GO_BP_FAT | GO:0002819~regulation of adaptive immune response | 16 | 3.64E-04 |  |
| GO_BP_FAT | GO:0031349~positive regulation of defense response | 16 | 4.48E-04 |  |
| GO_BP_FAT | GO:0042269~regulation of natural killer cell mediated cytotoxicity | 9 | 4.75E-04 |  |
| GO_BP_FAT | GO:0002715~regulation of natural killer cell mediated immunity | 9 | 4.75E-04 |  |
| GO_BP_FAT | GO:0002920~regulation of humoral immune response | 7 | 6.03E-04 |  |
| GO_BP_FAT | GO:0001916~positive regulation of T cell mediated cytotoxicity | 6 | 9.78E-04 |  |
| GO_BP_FAT | GO:0002717~positive regulation of natural killer cell mediated immunity | 7 | 2.49E-03 |  |
| GO_BP_FAT | GO:0045954~positive regulation of natural killer cell mediated cytotoxicity | 7 | 2.49E-03 |  |
| GO_BP_FAT | GO:0001914~regulation of T cell mediated cytotoxicity | 6 | 3.01E-03 |  |
| GO_BP_FAT | GO:0032729~positive regulation of interferon-gamma production | 7 | 5.16E-03 |  |
| GO_BP_FAT | GO:0002711~positive regulation of T cell mediated immunity | 6 | 7.05E-03 |  |
| GO_BP_FAT | GO:0045089~positive regulation of innate immune response | 10 | 8.70E-03 |  |
| GO_BP_FAT | GO:0002712~regulation of B cell mediated immunity | 9 | 8.75E-03 |  |
| GO_BP_FAT | GO:0002889~regulation of immunoglobulin mediated immune response | 9 | 8.75E-03 |  |
| GO_BP_FAT | GO:0002714~positive regulation of B cell mediated immunity | 6 | 1.01E-02 |  |
| GO_BP_FAT | GO:0002891~positive regulation of immunoglobulin mediated immune response | 6 | 1.01E-02 |  |
| GO_BP_FAT | GO:0002923~regulation of humoral immune response mediated by circulating immunoglobulin | 4 | 2.72E-02 |  |
| GO_BP_FAT | GO:0002475~antigen processing and presentation via MHC class Ib | 4 | 2.72E-02 |  |
| GO_BP_FAT | GO:0002709~regulation of T cell mediated immunity | 6 | 3.05E-02 |  |
| GO_BP_FAT | GO:0045428~regulation of nitric oxide biosynthetic process | 6 | 3.80E-02 |  |
| GO_BP_FAT | GO:0002218~activation of innate immune response | 5 | 4.71E-02 |  |
| GO_BP_FAT | GO:0002428~antigen processing and presentation of peptide antigen via MHC class Ib | 3 | 5.44E-02 |  |
| GO_BP_FAT | GO:0045429~positive regulation of nitric oxide biosynthetic process | 5 | 5.92E-02 |  |
| GO_BP_FAT | GO:0002925~positive regulation of humoral immune response mediated by circulating immunoglobulin | 3 | 8.46E-02 |  |
| GO_BP_FAT | GO:0002922~positive regulation of humoral immune response | 3 | 8.46E-02 |  |
| GO_BP_FAT | GO:0002758~innate immune response-activating signal transduction | 4 | 1.16E-01 |  |
| GO_MF_FAT | GO:0003823~antigen binding | 3 | 9.98E-01 |  |
|  |  |  |  |  |
| Cluster 18 |  |  |  |  |
| GO_MF_FAT | GO:0032555~purine ribonucleotide binding | 239 | 6.77E-06 | 3.23 |
| GO_MF_FAT | GO:0032553~ribonucleotide binding | 239 | 6.77E-06 |  |
| GO_MF_FAT | GO:0017076~purine nucleotide binding | 246 | 1.10E-05 |  |
| GO_MF_FAT | GO:0000166~nucleotide binding | 266 | 8.97E-04 |  |
| GO_MF_FAT | GO:0001882~nucleoside binding | 192 | 3.21E-03 |  |
| GO_MF_FAT | GO:0001883~purine nucleoside binding | 191 | 3.28E-03 |  |
| GO_MF_FAT | GO:0032559~adenyl ribonucleotide binding | 180 | 4.57E-03 |  |
| GO_MF_FAT | GO:0005524~ATP binding | 178 | 4.69E-03 |  |
| GO_MF_FAT | GO:0030554~adenyl nucleotide binding | 187 | 6.18E-03 |  |
| GO_MF_FAT | GO:0004672~protein kinase activity | 79 | 7.84E-03 |  |
|  |  |  |  |  |
| Cluster 19 |  |  |  |  |
| GO_BP_FAT | GO:0035295~tube development | 48 | 1.05E-04 | 3.19 |
| GO_BP_FAT | GO:0030324~lung development | 24 | 7.27E-04 |  |
| GO_BP_FAT | GO:0030323~respiratory tube development | 24 | 9.41E-04 |  |
| GO_BP_FAT | GO:0048286~lung alveolus development | 9 | 1.00E-03 |  |
| GO_BP_FAT | GO:0060541~respiratory system development | 25 | 1.54E-03 |  |
|  |  |  |  |  |
| Cluster 20 |  |  |  |  |
| GO_BP_FAT | GO:0019882~antigen processing and presentation | 26 | 1.14E-06 | 3.14 |
| GO_BP_FAT | GO:0048002~antigen processing and presentation of peptide antigen | 12 | 4.99E-04 |  |
| GO_BP_FAT | GO:0019884~antigen processing and presentation of exogenous antigen | 10 | 1.35E-03 |  |
| KEGG_PATHWAY | mmu04612:Antigen processing and presentation | 14 | 3.74E-01 |  |
|  |  |  |  |  |
| Cluster 21 |  |  |  |  |
| GO_BP_FAT | GO:0009967~positive regulation of signal transduction | 38 | 8.67E-06 | 2.96 |
| GO_BP_FAT | GO:0010647~positive regulation of cell communication | 38 | 7.58E-05 |  |
| GO_BP_FAT | GO:0010627~regulation of protein kinase cascade | 33 | 7.97E-05 |  |
| GO_BP_FAT | GO:0032755~positive regulation of interleukin-6 production | 9 | 1.99E-04 |  |
| GO_BP_FAT | GO:0032675~regulation of interleukin-6 production | 12 | 2.06E-04 |  |
| GO_BP_FAT | GO:0043408~regulation of MAPKKK cascade | 22 | 3.67E-04 |  |
| GO_BP_FAT | GO:0010740~positive regulation of protein kinase cascade | 21 | 4.59E-04 |  |
| GO_BP_FAT | GO:0070302~regulation of stress-activated protein kinase signaling pathway | 14 | 5.05E-04 |  |
| GO_BP_FAT | GO:0080135~regulation of cellular response to stress | 18 | 1.23E-03 |  |
| GO_BP_FAT | GO:0046328~regulation of JNK cascade | 13 | 1.44E-03 |  |
| GO_BP_FAT | GO:0043122~regulation of I-kappaB kinase/NF-kappaB cascade | 12 | 1.72E-03 |  |
| GO_BP_FAT | GO:0070304~positive regulation of stress-activated protein kinase signaling pathway | 7 | 3.65E-03 |  |
| GO_BP_FAT | GO:0046330~positive regulation of JNK cascade | 7 | 3.65E-03 |  |
| GO_BP_FAT | GO:0043123~positive regulation of I-kappaB kinase/NF-kappaB cascade | 10 | 4.71E-03 |  |
| GO_BP_FAT | GO:0043410~positive regulation of MAPKKK cascade | 12 | 6.66E-03 |  |
| GO_BP_FAT | GO:0032760~positive regulation of tumor necrosis factor production | 5 | 5.92E-02 |  |
| GO_BP_FAT | GO:0032680~regulation of tumor necrosis factor production | 5 | 2.95E-01 |  |
|  |  |  |  |  |
| Cluster 22 |  |  |  |  |
| GO_BP_FAT | GO:0043067~regulation of programmed cell death | 92 | 4.36E-06 | 2.95 |
| GO_BP_FAT | GO:0042981~regulation of apoptosis | 91 | 4.54E-06 |  |
| GO_BP_FAT | GO:0010941~regulation of cell death | 92 | 5.44E-06 |  |
| GO_BP_FAT | GO:0043065~positive regulation of apoptosis | 44 | 3.66E-04 |  |
| GO_BP_FAT | GO:0043068~positive regulation of programmed cell death | 44 | 4.36E-04 |  |
| GO_BP_FAT | GO:0010942~positive regulation of cell death | 44 | 5.18E-04 |  |
| GO_BP_FAT | GO:0012502~induction of programmed cell death | 30 | 3.02E-03 |  |
| GO_BP_FAT | GO:0006917~induction of apoptosis | 30 | 3.02E-03 |  |
| GO_BP_FAT | GO:0006916~anti-apoptosis | 15 | 6.20E-02 |  |
| GO_BP_FAT | GO:0043069~negative regulation of programmed cell death | 33 | 8.37E-02 |  |
| GO_BP_FAT | GO:0060548~negative regulation of cell death | 33 | 8.73E-02 |  |
| GO_BP_FAT | GO:0043066~negative regulation of apoptosis | 32 | 9.77E-02 |  |
|  |  |  |  |  |
| Cluster 23 |  |  |  |  |
| GO_BP_FAT | GO:0048002~antigen processing and presentation of peptide antigen | 12 | 4.99E-04 | 2.79 |
| GO_BP_FAT | GO:0002474~antigen processing and presentation of peptide antigen via MHC class I | 8 | 8.83E-04 |  |
| GO_BP_FAT | GO:0019883~antigen processing and presentation of endogenous antigen | 4 | 3.93E-03 |  |
| GO_BP_FAT | GO:0002483~antigen processing and presentation of endogenous peptide antigen | 4 | 3.93E-03 |  |
|  |  |  |  |  |
| Cluster 24 |  |  |  |  |
| GO_BP_FAT | GO:0030029~actin filament-based process | 33 | 8.70E-04 | 2.71 |
| GO_BP_FAT | GO:0030036~actin cytoskeleton organization | 31 | 1.24E-03 |  |
| GO_BP_FAT | GO:0007015~actin filament organization | 14 | 3.57E-03 |  |
| GO_BP_FAT | GO:0007010~cytoskeleton organization | 50 | 3.66E-03 |  |
|  |  |  |  |  |
| Cluster 25 |  |  |  |  |
| GO_BP_FAT | GO:0010324~membrane invagination | 36 | 3.23E-04 | 2.67 |
| GO_BP_FAT | GO:0006897~endocytosis | 36 | 3.23E-04 |  |
| GO_BP_FAT | GO:0016044~membrane organization | 44 | 2.46E-03 |  |
| GO_BP_FAT | GO:0016192~vesicle-mediated transport | 58 | 8.46E-02 |  |
|  |  |  |  |  |
| Cluster 26 |  |  |  |  |
| GO_BP_FAT | GO:0042098~T cell proliferation | 10 | 1.35E-03 | 2.61 |
| GO_BP_FAT | GO:0046651~lymphocyte proliferation | 12 | 2.63E-03 |  |
| GO_BP_FAT | GO:0070661~leukocyte proliferation | 12 | 3.21E-03 |  |
| GO_BP_FAT | GO:0032943~mononuclear cell proliferation | 12 | 3.21E-03 |  |
|  |  |  |  |  |
| Cluster 27 |  |  |  |  |
| GO_BP_FAT | GO:0016477~cell migration | 41 | 1.27E-03 | 2.57 |
| GO_BP_FAT | GO:0051674~localization of cell | 45 | 3.26E-03 |  |
| GO_BP_FAT | GO:0048870~cell motility | 45 | 3.26E-03 |  |
| GO_BP_FAT | GO:0006928~cell motion | 55 | 3.73E-03 |  |
|  |  |  |  |  |
| Cluster 28 |  |  |  |  |
| GO_MF_FAT | GO:0046983~protein dimerization activity | 55 | 6.94E-04 | 2.57 |
| GO_MF_FAT | GO:0042803~protein homodimerization activity | 34 | 2.18E-03 |  |
| GO_MF_FAT | GO:0042802~identical protein binding | 43 | 1.31E-02 |  |
|  |  |  |  |  |
| Cluster 29 |  |  |  |  |
| GO_BP_FAT | GO:0006468~protein amino acid phosphorylation | 94 | 2.71E-04 | 2.46 |
| GO_BP_FAT | GO:0016310~phosphorylation | 101 | 7.46E-04 |  |
| GO_BP_FAT | GO:0006793~phosphorus metabolic process | 117 | 1.17E-03 |  |
| GO_BP_FAT | GO:0006796~phosphate metabolic process | 117 | 1.17E-03 |  |
| GO_MF_FAT | GO:0032559~adenyl ribonucleotide binding | 180 | 4.57E-03 |  |
| GO_MF_FAT | GO:0005524~ATP binding | 178 | 4.69E-03 |  |
| GO_MF_FAT | GO:0030554~adenyl nucleotide binding | 187 | 6.18E-03 |  |
| GO_MF_FAT | GO:0004672~protein kinase activity | 79 | 7.84E-03 |  |
| GO_MF_FAT | GO:0004674~protein serine/threonine kinase activity | 49 | 2.30E-01 |  |
|  |  |  |  |  |
| Cluster 30 |  |  |  |  |
| GO_MF_FAT | GO:0008081~phosphoric diester hydrolase activity | 21 | 2.35E-05 | 2.45 |
| GO_MF_FAT | GO:0004112~cyclic-nucleotide phosphodiesterase activity | 9 | 2.59E-03 |  |
| GO_MF_FAT | GO:0004114~3',5'-cyclic-nucleotide phosphodiesterase activity | 8 | 8.39E-03 |  |
| GO_MF_FAT | GO:0047555~3',5'-cyclic-GMP phosphodiesterase activity | 3 | 3.13E-01 |  |
|  |  |  |  |  |
| Cluster 31 |  |  |  |  |
| KEGG_PATHWAY | mmu03010:Ribosome | 28 | 8.61E-06 | 2.37 |
| GO_MF_FAT | GO:0003735~structural constituent of ribosome | 28 | 2.78E-03 |  |
| GO_MF_FAT | GO:0005198~structural molecule activity | 60 | 2.72E-02 |  |
| GO_BP_FAT | GO:0006412~translation | 34 | 4.90E-01 |  |
|  |  |  |  |  |
| Cluster 32 |  |  |  |  |
| GO_BP_FAT | GO:0035295~tube development | 48 | 1.05E-04 | 2.37 |
| GO_BP_FAT | GO:0001763~morphogenesis of a branching structure | 23 | 7.71E-03 |  |
| GO_BP_FAT | GO:0035239~tube morphogenesis | 29 | 8.06E-03 |  |
| GO_BP_FAT | GO:0001569~patterning of blood vessels | 8 | 8.30E-03 |  |
| GO_BP_FAT | GO:0048754~branching morphogenesis of a tube | 17 | 2.56E-02 |  |
|  |  |  |  |  |
| Cluster 33 |  |  |  |  |
| GO_BP_FAT | GO:0044092~negative regulation of molecular function | 26 | 1.70E-03 | 2.26 |
| GO_BP_FAT | GO:0043086~negative regulation of catalytic activity | 21 | 2.76E-03 |  |
| GO_BP_FAT | GO:0033673~negative regulation of kinase activity | 13 | 4.48E-03 |  |
| GO_BP_FAT | GO:0006469~negative regulation of protein kinase activity | 13 | 4.48E-03 |  |
| GO_BP_FAT | GO:0051348~negative regulation of transferase activity | 13 | 6.22E-03 |  |
| GO_BP_FAT | GO:0043407~negative regulation of MAP kinase activity | 6 | 4.66E-02 |  |
|  |  |  |  |  |
| Cluster 34 |  |  |  |  |
| GO_MF_FAT | GO:0070003~threonine-type peptidase activity | 8 | 3.70E-03 | 2.25 |
| GO_MF_FAT | GO:0004298~threonine-type endopeptidase activity | 8 | 3.70E-03 |  |
| KEGG_PATHWAY | mmu03050:Proteasome | 13 | 1.26E-02 |  |
|  |  |  |  |  |
| Cluster 35 |  |  |  |  |
| GO_MF_FAT | GO:0030169~low-density lipoprotein binding | 8 | 1.14E-04 | 2.23 |
| GO_MF_FAT | GO:0008034~lipoprotein binding | 9 | 7.08E-04 |  |
| GO_MF_FAT | GO:0005041~low-density lipoprotein receptor activity | 6 | 9.87E-04 |  |
| GO_MF_FAT | GO:0030228~lipoprotein receptor activity | 7 | 1.03E-03 |  |
| GO_BP_FAT | GO:0015918~sterol transport | 3 | 6.97E-01 |  |
| GO_BP_FAT | GO:0030301~cholesterol transport | 3 | 6.97E-01 |  |
|  |  |  |  |  |
| Cluster 36 |  |  |  |  |
| GO_BP_FAT | GO:0032675~regulation of interleukin-6 production | 12 | 2.06E-04 | 2.23 |
| GO_BP_FAT | GO:0045410~positive regulation of interleukin-6 biosynthetic process | 4 | 2.72E-02 |  |
| GO_BP_FAT | GO:0045408~regulation of interleukin-6 biosynthetic process | 5 | 3.65E-02 |  |
|  |  |  |  |  |
| Cluster 37 |  |  |  |  |
| GO_BP_FAT | GO:0030155~regulation of cell adhesion | 20 | 2.74E-03 | 2.14 |
| GO_BP_FAT | GO:0010810~regulation of cell-substrate adhesion | 11 | 5.81E-03 |  |
| GO_BP_FAT | GO:0045785~positive regulation of cell adhesion | 11 | 9.92E-03 |  |
| GO_BP_FAT | GO:0010811~positive regulation of cell-substrate adhesion | 8 | 1.63E-02 |  |
|  |  |  |  |  |
| Cluster 38 |  |  |  |  |
| KEGG_PATHWAY | mmu04070:Phosphatidylinositol signaling system | 21 | 8.26E-04 | 2.13 |
| KEGG_PATHWAY | mmu00562:Inositol phosphate metabolism | 14 | 1.58E-02 |  |
| GO_MF_FAT | GO:0004437~inositol or phosphatidylinositol phosphatase activity | 7 | 3.05E-02 |  |
|  |  |  |  |  |
| Cluster 39 |  |  |  |  |
| GO_BP_FAT | GO:0060693~regulation of branching involved in salivary gland morphogenesis | 7 | 7.19E-05 | 2.10 |
| GO_BP_FAT | GO:0060638~mesenchymal-epithelial cell signaling | 4 | 1.68E-02 |  |
| GO_BP_FAT | GO:0060688~regulation of morphogenesis of a branching structure | 7 | 3.64E-02 |  |
| GO_BP_FAT | GO:0060665~regulation of branching involved in salivary gland morphogenesis by mesenchymal-epithelial signaling | 3 | 8.46E-02 |  |
|  |  |  |  |  |
| Cluster 40 |  |  |  |  |
| GO_BP_FAT | GO:0051099~positive regulation of binding | 13 | 1.44E-03 | 2.10 |
| GO_BP_FAT | GO:0043388~positive regulation of DNA binding | 12 | 2.63E-03 |  |
| GO_BP_FAT | GO:0070304~positive regulation of stress-activated protein kinase signaling pathway | 7 | 3.65E-03 |  |
| GO_BP_FAT | GO:0046330~positive regulation of JNK cascade | 7 | 3.65E-03 |  |
| GO_BP_FAT | GO:0051092~positive regulation of NF-kappaB transcription factor activity | 8 | 1.05E-02 |  |
| GO_BP_FAT | GO:0051101~regulation of DNA binding | 15 | 1.66E-02 |  |
| GO_BP_FAT | GO:0051091~positive regulation of transcription factor activity | 9 | 1.88E-02 |  |
| GO_BP_FAT | GO:0051098~regulation of binding | 17 | 1.92E-02 |  |
| GO_BP_FAT | GO:0051090~regulation of transcription factor activity | 12 | 3.47E-02 |  |
|  |  |  |  |  |
| Cluster 41 |  |  |  |  |
| GO_BP_FAT | GO:0002274~myeloid leukocyte activation | 12 | 4.99E-04 | 2.08 |
| GO_BP_FAT | GO:0002573~myeloid leukocyte differentiation | 10 | 5.84E-03 |  |
| GO_BP_FAT | GO:0001773~myeloid dendritic cell activation | 4 | 4.02E-02 |  |
| GO_BP_FAT | GO:0043011~myeloid dendritic cell differentiation | 4 | 4.02E-02 |  |
|  |  |  |  |  |
| Cluster 42 |  |  |  |  |
| GO_BP_FAT | GO:0032101~regulation of response to external stimulus | 22 | 1.50E-03 | 2.07 |
| GO_BP_FAT | GO:0032103~positive regulation of response to external stimulus | 10 | 1.48E-02 |  |
| GO_BP_FAT | GO:0050727~regulation of inflammatory response | 12 | 2.76E-02 |  |

^1^ Rows correspond to a category of Functional Annotation Tool (FAT) GO categories in each cluster

^2^ GO terms in each cluster
