# Supplementary material for: Microglia Transcriptome Changes in a Model of Depressive Behavior after Immune Challenge
Source: PLoS One. 2016 Mar 9;11(3):e0150858. doi: 10.1371/journal.pone.0150858 (PMC4784788; doi:10.1371/journal.pone.0150858)
Supplement: S3 Table — (DOCX) [file pone.0150858.s003.docx]

S3 Table. Gene Set Enrichment Analysis (GSEA) categories enriched among transcript isoforms over-expressed (FDR-adjusted P-value < 0.05 and > 10 transcript isoforms) and under- expressed (P-value < 0.05 and > 10 transcript isoforms) in BCG-challenged relative to Control mice in microglia.

| Categories | NG^1^ | P-value | FDR^2^ |
| --- | --- | --- | --- |
| Over-expressed in BCG-challenged vs Control Mice | | | |
| DEFENSE_RESPONSE | 147 | 0.00E+00 | 0.00E+00 |
| INFLAMMATORY_RESPONSE | 79 | 0.00E+00 | 0.00E+00 |
| RESPONSE_TO_OTHER_ORGANISM | 41 | 0.00E+00 | 0.00E+00 |
| IMMUNE_RESPONSE | 152 | 0.00E+00 | 0.00E+00 |
| RESPONSE_TO_VIRUS | 31 | 0.00E+00 | 0.00E+00 |
| RESPONSE_TO_EXTERNAL_STIMULUS | 183 | 0.00E+00 | 0.00E+00 |
| IMMUNE_SYSTEM_PROCESS | 219 | 0.00E+00 | 0.00E+00 |
| RESPONSE_TO_WOUNDING | 113 | 0.00E+00 | 0.00E+00 |
| KEGG_CYTOKINE_CYTOKINE_RECEPTOR_INTERACTION | 146 | 0.00E+00 | 0.00E+00 |
| MULTI_ORGANISM_PROCESS | 77 | 0.00E+00 | 0.00E+00 |
| RESPONSE_TO_BIOTIC_STIMULUS | 69 | 0.00E+00 | 0.00E+00 |
| LOCOMOTORY_BEHAVIOR | 57 | 0.00E+00 | 7.98E-05 |
| CATION_HOMEOSTASIS | 58 | 0.00E+00 | 8.65E-05 |
| CELLULAR_CATION_HOMEOSTASIS | 56 | 0.00E+00 | 1.49E-04 |
| G_PROTEIN_COUPLED_RECEPTOR_BINDING | 30 | 0.00E+00 | 2.80E-04 |
| ION_HOMEOSTASIS | 65 | 0.00E+00 | 3.30E-04 |
| KEGG_LEISHMANIA_INFECTION | 44 | 0.00E+00 | 1.06E-03 |
| CYTOKINE_ACTIVITY | 52 | 0.00E+00 | 1.11E-03 |
| CHEMOKINE_RECEPTOR_BINDING | 25 | 0.00E+00 | 1.11E-03 |
| KEGG_RIBOSOME | 80 | 0.00E+00 | 2.21E-03 |
| CELLULAR_HOMEOSTASIS | 74 | 0.00E+00 | 2.26E-03 |
| CHEMOKINE_ACTIVITY | 24 | 0.00E+00 | 4.01E-03 |
| KEGG_NOD_LIKE_RECEPTOR_SIGNALING_PATHWAY | 42 | 0.00E+00 | 4.06E-03 |
| KEGG_NATURAL_KILLER_CELL_MEDIATED_CYTOTOXICITY | 79 | 0.00E+00 | 6.35E-03 |
| CELLULAR_DEFENSE_RESPONSE | 33 | 0.00E+00 | 1.16E-02 |
| BEHAVIOR | 78 | 0.00E+00 | 1.23E-02 |
| RECEPTOR_SIGNALING_PROTEIN_ACTIVITY | 68 | 1.22E-03 | 1.36E-02 |
| KEGG_COMPLEMENT_AND_COAGULATION_CASCADES | 23 | 0.00E+00 | 1.46E-02 |
| KEGG_CHEMOKINE_SIGNALING_PATHWAY | 142 | 0.00E+00 | 1.60E-02 |
| CYTOKINE_BINDING | 34 | 0.00E+00 | 1.94E-02 |
| KEGG_CELL_ADHESION_MOLECULES_CAMS | 81 | 0.00E+00 | 2.22E-02 |
| CHEMICAL_HOMEOSTASIS | 83 | 0.00E+00 | 2.40E-02 |
| KEGG_T_CELL_RECEPTOR_SIGNALING_PATHWAY | 88 | 1.18E-03 | 2.94E-02 |
| APOPTOSIS_GO | 320 | 0.00E+00 | 2.98E-02 |
| KEGG_HEMATOPOIETIC_CELL_LINEAGE | 46 | 0.00E+00 | 3.10E-02 |
| JAK_STAT_CASCADE | 24 | 2.95E-03 | 3.23E-02 |
| LEUKOCYTE_DIFFERENTIATION | 26 | 1.46E-03 | 3.29E-02 |
| PROGRAMMED_CELL_DEATH | 321 | 0.00E+00 | 3.29E-02 |
| INTERLEUKIN_BINDING | 17 | 6.23E-03 | 3.96E-02 |
| IMMUNE_EFFECTOR_PROCESS | 20 | 4.60E-03 | 4.18E-02 |
| REGULATION_OF_APOPTOSIS | 254 | 0.00E+00 | 4.18E-02 |
| KEGG_PRIMARY_IMMUNODEFICIENCY | 24 | 4.45E-03 | 4.23E-02 |
| Under-expressed in BCG-challenged vs Control | | | |
| NERVOUS_SYSTEM_DEVELOPMENT | 228 | 0.00E+00 | 1.00E+00 |
| POLYSACCHARIDE_BINDING | 19 | 2.85E-03 | 7.91E-01 |
| GLYCOSAMINOGLYCAN_BINDING | 18 | 5.46E-03 | 5.51E-01 |
| KEGG_INOSITOL_PHOSPHATE_METABOLISM | 42 | 8.10E-03 | 7.12E-01 |
| CENTRAL_NERVOUS_SYSTEM_DEVELOPMENT | 73 | 1.24E-02 | 9.92E-01 |
| PROTEIN_SECRETION | 16 | 1.43E-02 | 6.71E-01 |
| TRANSMISSION_OF_NERVE_IMPULSE | 85 | 2.27E-02 | 6.72E-01 |
| KEGG_PARKINSONS_DISEASE | 94 | 3.03E-02 | 7.31E-01 |
| CELLULAR_PROTEIN_COMPLEX_ASSEMBLY | 30 | 4.28E-02 | 1.00E+00 |
| SYNAPTIC_TRANSMISSION | 75 | 4.76E-02 | 8.86E-01 |

^1^NG: number of genes

^2^FDR: adjusted P-value
