# Supplementary material for: Microglia Transcriptome Changes in a Model of Depressive Behavior after Immune Challenge
Source: PLoS One. 2016 Mar 9;11(3):e0150858. doi: 10.1371/journal.pone.0150858 (PMC4784788; doi:10.1371/journal.pone.0150858)
Supplement: S4 Table — (DOCX) [file pone.0150858.s004.docx]

S4 Table. Gene Set Enrichment Analysis (GSEA) categories enriched among transcript isoforms over-expressed (FDR-adjusted P-value < 0.05 and > 10 transcript isoforms) and under- expressed (FDR-adjusted P-value < 0.05 and > 10 transcript isoforms) in BCG-challenged relative to Control in macrophages.

| Categories | NG^1^ | P-value | FDR ^2^ |
| --- | --- | --- | --- |
| Over -expressed in BCG-challenged vs Control Mice | | | |
| KEGG_ECM_RECEPTOR_INTERACTION | 45 | 0.00E+00 | 9.96E-04 |
| DEFENSE_RESPONSE | 144 | 0.00E+00 | 5.88E-03 |
| INFLAMMATORY_RESPONSE | 73 | 0.00E+00 | 1.28E-02 |
| SERINE_TYPE_PEPTIDASE_ACTIVITY | 18 | 0.00E+00 | 1.31E-02 |
| SERINE_HYDROLASE_ACTIVITY | 19 | 0.00E+00 | 1.32E-02 |
| KEGG_PROTEASOME | 43 | 0.00E+00 | 1.48E-02 |
| KEGG_FOCAL_ADHESION | 132 | 0.00E+00 | 2.11E-02 |
| TRANSMEMBRANE_RECEPTOR_ACTIVITY | 155 | 0.00E+00 | 2.32E-02 |
| KEGG_TOLL_LIKE_RECEPTOR_SIGNALING_PATHWAY | 79 | 0.00E+00 | 2.45E-02 |
| RESPONSE_TO_VIRUS | 29 | 0.00E+00 | 2.46E-02 |
| ENDOPEPTIDASE_ACTIVITY | 61 | 0.00E+00 | 2.61E-02 |
| RESPONSE_TO_OTHER_ORGANISM | 41 | 0.00E+00 | 2.65E-02 |
| APOPTOTIC_PROGRAM | 44 | 1.45E-03 | 2.65E-02 |
| KEGG_CYTOKINE_CYTOKINE_RECEPTOR_INTERACTION | 152 | 0.00E+00 | 2.73E-02 |
| KEGG_ANTIGEN_PROCESSING_AND_PRESENTATION | 32 | 1.53E-03 | 2.78E-02 |
| CELLULAR_DEFENSE_RESPONSE | 35 | 1.52E-03 | 2.80E-02 |
| CALCIUM_ION_BINDING | 53 | 1.42E-03 | 2.82E-02 |
| KEGG_NATURAL_KILLER_CELL_MEDIATED_CYTOTOXICITY | 78 | 2.65E-03 | 3.02E-02 |
| RECEPTOR_ACTIVITY | 246 | 0.00E+00 | 3.11E-02 |
| RESPONSE_TO_BIOTIC_STIMULUS | 70 | 0.00E+00 | 3.17E-02 |
| MULTI_ORGANISM_PROCESS | 69 | 0.00E+00 | 3.21E-02 |
| KEGG_CYTOSOLIC_DNA_SENSING_PATHWAY | 36 | 2.97E-03 | 3.22E-02 |
| RESPONSE_TO_WOUNDING | 105 | 1.29E-03 | 3.52E-02 |
| Under-expressed in BCG-challenged vs Control Mice | | | |
| KEGG_RIBOSOME | 78 | 0.00E+00 | 0.00E+00 |
| STRUCTURAL_CONSTITUENT_OF_RIBOSOME | 75 | 0.00E+00 | 0.00E+00 |
| GATED_CHANNEL_ACTIVITY | 27 | 0.00E+00 | 0.00E+00 |
| CATION_CHANNEL_ACTIVITY | 29 | 0.00E+00 | 2.39E-04 |
| VOLTAGE_GATED_CHANNEL_ACTIVITY | 19 | 0.00E+00 | 2.06E-03 |
| VOLTAGE_GATED_CATION_CHANNEL_ACTIVITY | 17 | 0.00E+00 | 2.48E-03 |

^1^NG: number of genes

^2^FDR: adjusted P-value
