# Supplementary material for: Microglia Transcriptome Changes in a Model of Depressive Behavior after Immune Challenge
Source: PLoS One. 2016 Mar 9;11(3):e0150858. doi: 10.1371/journal.pone.0150858 (PMC4784788; doi:10.1371/journal.pone.0150858)
Supplement: S5 Table — (DOCX) [file pone.0150858.s005.docx]

S5 Table. Differentially expressed genes (FDR-adjusted P-value < 2.0 x 10^-4^) between microglia and macrophages in BCG-challenged mice and supporting literature review.

| Gene Symbol | NCBI Gene Name | Log_2_(Macrophage/Microglia) | Reference |
| --- | --- | --- | --- |
| Col8a1 | collagen, type VIII, alpha 1 | -13.6983 | Buga et al. (2012) |
| Kcne2 | potassium voltage-gated channel, Isk-related subfamily, gene 2 | -13.6282 | Jou et al. (1998) |
| Slc38a5 | solute carrier family 38, member 5 | -11.6051 |  |
| Lrrc3 | predicted gene 884 | -11.4898 | Butovsky et al. (2014) |
| Nav3 | neuron navigator 3 | -11.2728 | Fumagalli et al. (2015) |
| Gm694 | predicted gene 694 | -11.0715 |  |
| Capn3 | calpain 3 | -11.056 | Kim et al. (2006) |
| Gpr34 | G protein-coupled receptor 34 | -10.4916 | Hickman et al. (2013); Butovsky et al. (2014);  Fumagalli et al. (2015) |
| Lrrn1 | leucine rich repeat protein 1, neuronal | -10.3003 | Solga et al. (2015); |
| Wwox | WW domain-containing oxidoreductase | -10.2352 | Teng et al.(2013) |
| Vstm4 | V-set and transmembrane domain containing 4 | -10.0689 |  |
| Ecscr | endothelial cell surface expressed chemotaxis and apoptosis regulator | -9.95078 | Hickman et al. (2013) |
| Cenpf | centromere protein F | -9.92911 |  |
| Gm10790 | predicted gene 10790 | -9.9266 |  |
| Tspan7 | tetraspanin 7 | -9.78541 | Butovsky et al. (2014) |
| Epn3 | epsin 3 | -9.76245 |  |
| Tmem100 | transmembrane protein 100 | -9.72567 | Butovsky et al. (2014) |
| Leprel1 | prolyl 3-hydroxylase 2 | -9.71621 | Butovsky et al. (2014) |
| Ebf3 | early B cell factor 3 | -9.70589 | Butovsky et al. (2014) |
| Rnu11 | U11 small nuclear RNA | -9.6457 |  |
| Wdr52 | cilia and flagella associated protein 44 | -9.6045 |  |
| Olfml3 | olfactomedin-like 3 | -9.60081 | Hickman et al. (2013); Butovsky et al. (2014); |
| Trpm3 | transient receptor potential cation channel, subfamily M, member 3 | -9.57651 |  |
| Fat3 | FAT tumor suppressor homolog 3 (Drosophila) | -9.55715 |  |
| Adcy2 | adenylate cyclase 2 | -9.51678 |  |
| Kcnj13 | potassium inwardly-rectifying channel, subfamily J, member 13 | -9.48316 |  |
| Gpr56 | adhesion G protein-coupled receptor G1 | -9.45891 | Hickman et al. (2013); Butovsky et al. (2014); |
| Sema4g | sema domain, immunoglobulin domain (Ig), transmembrane domain (TM) and short cytoplasmic domain, (semaphorin) 4G | -9.45237 |  |
| Synm | synemin, intermediate filament protein | -9.40223 |  |
| Six3 | sine oculis-related homeobox 3 | -9.38807 |  |
| Slc4a10 | solute carrier family 4, sodium bicarbonate cotransporter-like, member 10 | -9.3566 |  |
| Mboat1 | membrane bound O-acyltransferase domain containing 1 | -9.07669 |  |
| Naalad2 | N-acetylated alpha-linked acidic dipeptidase 2 | -8.83591 |  |
| Folr1 | folate receptor 1 (adult) | -8.73546 |  |
| Notch3 | notch 3 | -8.73217 |  |
| Serpine2 | serine (or cysteine) peptidase inhibitor, clade E, member 2 | -8.72544 | Elmore et al. (2014); Butovsky et al. (2014) |
| Atp8a2 | ATPase, aminophospholipid transporter-like, class I, type 8A, member 2 | -8.72364 | Butovsky et al. (2014) |
| Fbln7 | fibulin 7 | -8.66986 |  |
| Cldn9 | claudin 9 | -8.65 |  |
| Sgce | sarcoglycan, epsilon | -8.63631 | Butovsky et al. (2014) |
| Slc4a5 | solute carrier family 4, sodium bicarbonate cotransporter, member 5 | -8.59133 |  |
| Adamts1 | a disintegrin-like and metallopeptidase (reprolysin type) with thrombospondin type 1 motif, 1 | -8.5462 | Butovsky et al. (2014) |
| Spock2 | sparc/osteonectin, cwcv and kazal-like domains proteoglycan 2 | -8.48861 |  |
| Ajap1 | adherens junction associated protein 1 | -8.46617 |  |
| Adrb1 | adrenergic receptor, beta 1 | -8.43629 | Butovsky et al. (2014) |
| C1qtnf5 | C1q and tumor necrosis factor related protein 5 | -8.41925 |  |
| Siglech | sialic acid binding Ig-like lectin H | -8.36015 | Hickman et al. (2013); Butovsky et al. (2014) |
| Ctxn3 | cortexin 3 | -8.31984 |  |
| Myl9 | myosin, light polypeptide 9, regulatory | -8.30974 |  |
| Arhgef40 | Rho guanine nucleotide exchange factor (GEF) 40 | -8.24948 |  |
| Chac1 | ChaC, cation transport regulator 1 | 6.30367 |  |
| H-2Eb | histocompatibility 2, class II antigen E beta | 6.36771 | Fumagalli et al. (2015) |
| Fam20c | family with sequence similarity 20, member C | 6.39259 |  |
| Ptger2 | prostaglandin E receptor 2 (subtype EP2) | 6.40544 |  |
| Tnip3 | TNFAIP3 interacting protein 3 | 6.42422 | Xue et al. (2014) |
| Mir155 | microRNA 155 | 6.4394 | Ghorpade et al. (2012) |
| Trib3 | tribbles homolog 3 (Drosophila) | 6.47759 |  |
| Htr7 | 5-hydroxytryptamine (serotonin) receptor 7 | 6.50378 |  |
| Ms4a8a | membrane-spanning 4-domains, subfamily A, member 8A | 6.50644 | Gautier et al. (2012) |
| Faim3 | Fas apoptotic inhibitory molecule 3 | 6.67598 |  |
| Tpsg1 | tryptase gamma 1 | 6.69364 |  |
| Cacna1i | calcium channel, voltage-dependent, alpha 1I subunit | 6.71639 |  |
| Tnfrsf13c | tumor necrosis factor receptor superfamily, member 13c | 6.74423 |  |
| Ly6d | lymphocyte antigen 6 complex, locus D | 6.78304 |  |
| Cdh17 | cadherin 17 | 6.82899 |  |
| Slc7a2 | solute carrier family 7 (cationic amino acid transporter, y+ system), member 2 | 6.83799 | Gautier et al. (2012) |
| Pax5 | paired box 5 | 6.85151 |  |
| Stac2 | SH3 and cysteine rich domain 2 | 6.85539 |  |
| Ecm1 | extracellular matrix protein 1 | 6.89446 | Hickman et al. (2013) |
| F10 | coagulation factor X | 6.89555 |  |
| Spib | Spi-B transcription factor (Spi-1/PU.1 related) | 6.90261 |  |
| Clec2g | C-type lectin domain family 2, member g | 6.96203 |  |
| Cxcr5 | chemokine (C-X-C motif) receptor 5 | 6.98314 | Hickman et al. (2013) |
| Cxcl3 | chemokine (C-X-C motif) ligand 3 | 7.06758 | Hickman et al. (2013) |
| Flrt3 | fibronectin leucine rich transmembrane protein 3 | 7.13574 | Hickman et al. (2013) |
| Selplg | P-selectin glycoprotein ligand 1 | 7.16327 | Hickman et al. (2013) |
| Cd19 | CD19 antigen | 7.16665 | Hickman et al. (2013) |
| Retnla | resistin like alpha | 7.25 | Hickman et al. (2013) |
| Mzb1 | marginal zone B and B1 cell-specific protein 1 | 7.34545 |  |
| Tnfrsf8 | tumor necrosis factor receptor superfamily, member 8 | 7.41532 |  |
| Cd79a | CD79A antigen (immunoglobulin-associated alpha) | 7.49477 |  |
| Mcoln2 | mucolipin 2 | 7.53712 |  |
| Vsig4 | V-set and immunoglobulin domain containing 4 | 7.553 |  |
| Fcrla | Fc receptor-like A | 7.63801 |  |
| Ms4a1 | membrane-spanning 4-domains, subfamily A, member 1 | 7.67924 |  |
| Cd163l1 | CD163 molecule-like 1 | 7.76079 |  |
| Gjb5 | gap junction protein, beta 5 | 7.85571 |  |
| Vmn2r24 | vomeronasal 2, receptor 24 | 7.85854 |  |
| Scn4a | sodium channel, voltage-gated, type IV, alpha | 7.87699 |  |
| Il9r | interleukin 9 receptor | 7.94505 |  |
| Blk | B lymphoid kinase | 8.00618 |  |
| F7 | coagulation factor VII | 8.10781 | Gautier et al. (2012) |
| Fcrl5 | Fc receptor-like 5 | 8.14458 |  |
| Serpinb2 | serine (or cysteine) peptidase inhibitor, clade B, member 2 | 8.20548 | Hickman et al. (2013) |
| Pou2af1 | POU domain, class 2, associating factor 1 | 8.43718 |  |
| Ptges | prostaglandin E synthase | 8.66221 | Milano et al (1995) |
| Cxcl13 | chemokine (C-X-C motif) ligand 13 | 9.2984 | Hickman et al. (2013) |
| Nos2 | nitric oxide synthase 2, inducible | 9.33061 | MacMicking et al. (1997) |
| Cd5l | CD5 antigen-like | 9.34107 | Hickman et al. (2013) |
| Arg1 | arginase, liver | 10.049 | Weisser et al.(2011) |
