# Supplementary material for: Microglia Transcriptome Changes in a Model of Depressive Behavior after Immune Challenge
Source: PLoS One. 2016 Mar 9;11(3):e0150858. doi: 10.1371/journal.pone.0150858 (PMC4784788; doi:10.1371/journal.pone.0150858)
Supplement: S6 Table — (DOCX) [file pone.0150858.s006.docx]

S6 Table. Functional cluster (DAVID Enrichment score ES > 2) of categories enriched among differentially expressed transcript isoforms between microglia and macrophages in BCG-challenged mice.

| Cluster and Category^1^ | Terms^2^ | Count | P-value | ES |
| --- | --- | --- | --- | --- |
| Cluster 1 |  |  |  |  |
| GO_BP_FAT | GO:0009611~response to wounding | 84 | 2.10E-11 | **8.72** |
| GO_BP_FAT | GO:0006954~inflammatory response | 57 | 8.87E-09 |  |
| GO_BP_FAT | GO:0006952~defense response | 91 | 3.83E-08 |  |
|  |  |  |  |  |
| Cluster 2 |  |  |  |  |
| GO_BP_FAT | GO:0050865~regulation of cell activation | 51 | 3.48E-12 | **6.72** |
| GO_BP_FAT | GO:0002694~regulation of leukocyte activation | 50 | 7.73E-12 |  |
| GO_BP_FAT | GO:0002684~positive regulation of immune system process | 58 | 8.51E-11 |  |
| GO_BP_FAT | GO:0051249~regulation of lymphocyte activation | 45 | 3.92E-10 |  |
| GO_BP_FAT | GO:0050867~positive regulation of cell activation | 35 | 2.37E-09 |  |
| GO_BP_FAT | GO:0002696~positive regulation of leukocyte activation | 34 | 5.39E-09 |  |
| GO_BP_FAT | GO:0051251~positive regulation of lymphocyte activation | 32 | 2.08E-08 |  |
| GO_BP_FAT | GO:0070663~regulation of leukocyte proliferation | 29 | 4.26E-08 |  |
| GO_BP_FAT | GO:0032944~regulation of mononuclear cell proliferation | 28 | 9.75E-08 |  |
| GO_BP_FAT | GO:0050670~regulation of lymphocyte proliferation | 28 | 9.75E-08 |  |
| GO_BP_FAT | GO:0050863~regulation of T cell activation | 33 | 2.10E-07 |  |
| GO_BP_FAT | GO:0070665~positive regulation of leukocyte proliferation | 21 | 4.03E-07 |  |
| GO_BP_FAT | GO:0050671~positive regulation of lymphocyte proliferation | 20 | 9.76E-07 |  |
| GO_BP_FAT | GO:0032946~positive regulation of mononuclear cell proliferation | 20 | 9.76E-07 |  |
| GO_BP_FAT | GO:0050870~positive regulation of T cell activation | 23 | 1.88E-06 |  |
| GO_BP_FAT | GO:0042129~regulation of T cell proliferation | 19 | 6.12E-05 |  |
| GO_BP_FAT | GO:0042102~positive regulation of T cell proliferation | 14 | 6.71E-05 |  |
| GO_BP_FAT | GO:0050864~regulation of B cell activation | 18 | 8.12E-05 |  |
| GO_BP_FAT | GO:0030888~regulation of B cell proliferation | 13 | 1.15E-04 |  |
| GO_BP_FAT | GO:0050871~positive regulation of B cell activation | 13 | 5.48E-04 |  |
| GO_BP_FAT | GO:0030890~positive regulation of B cell proliferation | 9 | 2.03E-03 |  |
|  |  |  |  |  |
| Cluster 3 |  |  |  |  |
| GO_BP_FAT | GO:0016477~cell migration | 59 | 1.53E-08 | **6.55** |
| GO_BP_FAT | GO:0051674~localization of cell | 62 | 5.98E-07 |  |
| GO_BP_FAT | GO:0048870~cell motility | 62 | 5.98E-07 |  |
| GO_BP_FAT | GO:0006928~cell motion | 74 | 1.10E-06 |  |
|  |  |  |  |  |
| Cluster 4 |  |  |  |  |
| GO_BP_FAT | GO:0001817~regulation of cytokine production | 43 | 1.43E-09 | **6.44** |
| GO_BP_FAT | GO:0042035~regulation of cytokine biosynthetic process | 23 | 1.04E-06 |  |
| GO_BP_FAT | GO:0042108~positive regulation of cytokine biosynthetic process | 16 | 3.21E-05 |  |
|  |  |  |  |  |
| Cluster 5 |  |  |  |  |
| GO_MF_FAT | GO:0017076~purine nucleotide binding | 294 | 1.82E-10 | **6.14** |
| GO_MF_FAT | GO:0032553~ribonucleotide binding | 282 | 5.53E-10 |  |
| GO_MF_FAT | GO:0032555~purine ribonucleotide binding | 282 | 5.53E-10 |  |
| GO_MF_FAT | GO:0000166~nucleotide binding | 320 | 5.90E-08 |  |
| GO_BP_FAT | GO:0006796~phosphate metabolic process | 146 | 7.03E-07 |  |
| GO_BP_FAT | GO:0006793~phosphorus metabolic process | 146 | 7.03E-07 |  |
| GO_MF_FAT | GO:0001882~nucleoside binding | 232 | 2.10E-06 |  |
| GO_MF_FAT | GO:0030554~adenyl nucleotide binding | 229 | 2.20E-06 |  |
| GO_MF_FAT | GO:0001883~purine nucleoside binding | 230 | 2.83E-06 |  |
| GO_BP_FAT | GO:0006468~protein amino acid phosphorylation | 111 | 4.83E-06 |  |
| GO_MF_FAT | GO:0005524~ATP binding | 215 | 5.33E-06 |  |
| GO_MF_FAT | GO:0032559~adenyl ribonucleotide binding | 217 | 5.63E-06 |  |
| GO_BP_FAT | GO:0016310~phosphorylation | 120 | 1.19E-05 |  |
| GO_MF_FAT | GO:0004672~protein kinase activity | 96 | 1.02E-04 |  |
| GO_MF_FAT | GO:0004674~protein serine/threonine kinase activity | 68 | 1.90E-03 |  |
|  |  |  |  |  |
| Cluster 6 |  |  |  |  |
| GO_BP_FAT | GO:0042330~taxis | 38 | 3.59E-10 | **6.04** |
| GO_BP_FAT | GO:0006935~chemotaxis | 38 | 3.59E-10 |  |
| GO_MF_FAT | GO:0008009~chemokine activity | 17 | 1.16E-06 |  |
| GO_MF_FAT | GO:0042379~chemokine receptor binding | 17 | 1.77E-06 |  |
| GO_MF_FAT | GO:0005125~cytokine activity | 40 | 3.70E-05 |  |
| GO_BP_FAT | GO:0007626~locomotory behavior | 49 | 5.77E-05 |  |
| GO_BP_FAT | GO:0007610~behavior | 68 | 9.90E-04 |  |
|  |  |  |  |  |
| Cluster 7 |  |  |  |  |
| GO_BP_FAT | GO:0008219~cell death | 96 | 4.94E-07 | **5.92** |
| GO_BP_FAT | GO:0016265~death | 97 | 7.85E-07 |  |
| GO_BP_FAT | GO:0012501~programmed cell death | 89 | 1.78E-06 |  |
| GO_BP_FAT | GO:0006915~apoptosis | 87 | 2.96E-06 |  |
|  |  |  |  |  |
| Cluster 8 |  |  |  |  |
| GO_BP_FAT | GO:0042981~regulation of apoptosis | 111 | 2.02E-09 | **5.53** |
| GO_BP_FAT | GO:0043067~regulation of programmed cell death | 112 | 2.12E-09 |  |
| GO_BP_FAT | GO:0010941~regulation of cell death | 112 | 2.88E-09 |  |
| GO_BP_FAT | GO:0043068~positive regulation of programmed cell death | 57 | 4.15E-07 |  |
| GO_BP_FAT | GO:0010942~positive regulation of cell death | 57 | 5.46E-07 |  |
| GO_BP_FAT | GO:0043065~positive regulation of apoptosis | 55 | 1.69E-06 |  |
| GO_BP_FAT | GO:0006917~induction of apoptosis | 37 | 1.08E-04 |  |
| GO_BP_FAT | GO:0012502~induction of programmed cell death | 37 | 1.08E-04 |  |
| GO_BP_FAT | GO:0043069~negative regulation of programmed cell death | 45 | 1.28E-03 |  |
| GO_BP_FAT | GO:0060548~negative regulation of cell death | 45 | 1.40E-03 |  |
| GO_BP_FAT | GO:0043066~negative regulation of apoptosis | 44 | 1.52E-03 |  |
|  |  |  |  |  |
| Cluster 9 |  |  |  |  |
| GO_BP_FAT | GO:0050865~regulation of cell activation | 51 | 3.48E-12 | **5.26** |
| GO_BP_FAT | GO:0002694~regulation of leukocyte activation | 50 | 7.73E-12 |  |
| GO_BP_FAT | GO:0051249~regulation of lymphocyte activation | 45 | 3.92E-10 |  |
| GO_BP_FAT | GO:0070663~regulation of leukocyte proliferation | 29 | 4.26E-08 |  |
| GO_BP_FAT | GO:0032944~regulation of mononuclear cell proliferation | 28 | 9.75E-08 |  |
| GO_BP_FAT | GO:0050670~regulation of lymphocyte proliferation | 28 | 9.75E-08 |  |
| GO_BP_FAT | GO:0050863~regulation of T cell activation | 33 | 2.10E-07 |  |
| GO_BP_FAT | GO:0002683~negative regulation of immune system process | 25 | 1.95E-06 |  |
| GO_BP_FAT | GO:0050866~negative regulation of cell activation | 18 | 4.80E-05 |  |
| GO_BP_FAT | GO:0002695~negative regulation of leukocyte activation | 18 | 4.80E-05 |  |
| GO_BP_FAT | GO:0042129~regulation of T cell proliferation | 19 | 6.12E-05 |  |
| GO_BP_FAT | GO:0051250~negative regulation of lymphocyte activation | 17 | 1.39E-04 |  |
| GO_BP_FAT | GO:0050868~negative regulation of T cell activation | 13 | 1.91E-03 |  |
| GO_BP_FAT | GO:0070664~negative regulation of leukocyte proliferation | 11 | 4.47E-03 |  |
| GO_BP_FAT | GO:0032945~negative regulation of mononuclear cell proliferation | 11 | 4.47E-03 |  |
| GO_BP_FAT | GO:0050672~negative regulation of lymphocyte proliferation | 11 | 4.47E-03 |  |
| GO_BP_FAT | GO:0042130~negative regulation of T cell proliferation | 8 | 3.37E-02 |  |
| GO_BP_FAT | GO:0030889~negative regulation of B cell proliferation | 4 | 9.58E-02 |  |
|  |  |  |  |  |
| Cluster 10 |  |  |  |  |
| GO_BP_FAT | GO:0001944~vasculature development | 55 | 2.19E-06 | **4.59** |
| GO_BP_FAT | GO:0001568~blood vessel development | 52 | 1.10E-05 |  |
| GO_BP_FAT | GO:0048514~blood vessel morphogenesis | 44 | 2.00E-05 |  |
| GO_BP_FAT | GO:0001525~angiogenesis | 29 | 8.90E-04 |  |
|  |  |  |  |  |
| Cluster 11 |  |  |  |  |
| GO_BP_FAT | GO:0048534~hemopoietic or lymphoid organ development | 65 | 3.23E-08 | **4.59** |
| GO_BP_FAT | GO:0002520~immune system development | 65 | 2.17E-07 |  |
| GO_BP_FAT | GO:0030097~hemopoiesis | 57 | 4.76E-07 |  |
| GO_BP_FAT | GO:0001775~cell activation | 56 | 5.60E-07 |  |
| GO_BP_FAT | GO:0045321~leukocyte activation | 51 | 9.43E-07 |  |
| GO_BP_FAT | GO:0002521~leukocyte differentiation | 36 | 6.67E-06 |  |
| GO_BP_FAT | GO:0042110~T cell activation | 30 | 3.01E-05 |  |
| GO_BP_FAT | GO:0046649~lymphocyte activation | 42 | 4.11E-05 |  |
| GO_BP_FAT | GO:0030098~lymphocyte differentiation | 28 | 1.50E-04 |  |
| GO_BP_FAT | GO:0030183~B cell differentiation | 14 | 1.40E-03 |  |
| GO_BP_FAT | GO:0030099~myeloid cell differentiation | 22 | 2.28E-03 |  |
| GO_BP_FAT | GO:0030217~T cell differentiation | 17 | 1.05E-02 |  |
| GO_BP_FAT | GO:0042113~B cell activation | 16 | 2.83E-02 |  |
|  |  |  |  |  |
| Cluster 12 |  |  |  |  |
| GO_BP_FAT | GO:0001817~regulation of cytokine production | 43 | 1.43E-09 | **4.26** |
| GO_BP_FAT | GO:0032649~regulation of interferon-gamma production | 13 | 1.15E-04 |  |
| GO_BP_FAT | GO:0001819~positive regulation of cytokine production | 17 | 1.22E-03 |  |
| GO_BP_FAT | GO:0051240~positive regulation of multicellular organismal process | 27 | 4.70E-02 |  |
|  |  |  |  |  |
| Cluster 13 |  |  |  |  |
| GO_MF_FAT | GO:0005525~GTP binding | 68 | 1.08E-05 | **4.07** |
| GO_MF_FAT | GO:0032561~guanyl ribonucleotide binding | 68 | 2.50E-05 |  |
| GO_MF_FAT | GO:0019001~guanyl nucleotide binding | 68 | 2.50E-05 |  |
| GO_MF_FAT | GO:0003924~GTPase activity | 25 | 7.84E-03 |  |
|  |  |  |  |  |
| Cluster 14 |  |  |  |  |
| GO_BP_FAT | GO:0007155~cell adhesion | 100 | 4.61E-06 | **3.85** |
| GO_BP_FAT | GO:0022610~biological adhesion | 100 | 5.00E-06 |  |
| GO_BP_FAT | GO:0016337~cell-cell adhesion | 34 | 1.23E-01 |  |
|  |  |  |  |  |
| Cluster 15 |  |  |  |  |
| GO_BP_FAT | GO:0014033~neural crest cell differentiation | 14 | 3.24E-05 | **3.82** |
| GO_BP_FAT | GO:0014032~neural crest cell development | 14 | 3.24E-05 |  |
| GO_BP_FAT | GO:0014031~mesenchymal cell development | 17 | 3.49E-05 |  |
| GO_BP_FAT | GO:0048762~mesenchymal cell differentiation | 17 | 6.24E-05 |  |
| GO_BP_FAT | GO:0060485~mesenchyme development | 17 | 8.22E-05 |  |
| GO_BP_FAT | GO:0001755~neural crest cell migration | 9 | 2.81E-03 |  |
| GO_BP_FAT | GO:0001667~ameboidal cell migration | 13 | 3.60E-03 |  |
|  |  |  |  |  |
| Cluster 16 |  |  |  |  |
| GO_BP_FAT | GO:0043549~regulation of kinase activity | 43 | 2.07E-05 | **3.54** |
| GO_BP_FAT | GO:0045859~regulation of protein kinase activity | 41 | 4.86E-05 |  |
| GO_BP_FAT | GO:0051338~regulation of transferase activity | 43 | 5.05E-05 |  |
| GO_BP_FAT | GO:0044092~negative regulation of molecular function | 30 | 3.47E-04 |  |
| GO_BP_FAT | GO:0006469~negative regulation of protein kinase activity | 16 | 3.85E-04 |  |
| GO_BP_FAT | GO:0033673~negative regulation of kinase activity | 16 | 3.85E-04 |  |
| GO_BP_FAT | GO:0051348~negative regulation of transferase activity | 16 | 6.07E-04 |  |
| GO_BP_FAT | GO:0043086~negative regulation of catalytic activity | 23 | 1.92E-03 |  |
| GO_BP_FAT | GO:0043407~negative regulation of MAP kinase activity | 8 | 4.94E-03 |  |
|  |  |  |  |  |
| Cluster 17 |  |  |  |  |
| GO_BP_FAT | GO:0042325~regulation of phosphorylation | 59 | 1.17E-05 | **3.15** |
| GO_BP_FAT | GO:0043549~regulation of kinase activity | 43 | 2.07E-05 |  |
| GO_BP_FAT | GO:0051174~regulation of phosphorus metabolic process | 59 | 3.64E-05 |  |
| GO_BP_FAT | GO:0019220~regulation of phosphate metabolic process | 59 | 3.64E-05 |  |
| GO_BP_FAT | GO:0045859~regulation of protein kinase activity | 41 | 4.86E-05 |  |
| GO_BP_FAT | GO:0051338~regulation of transferase activity | 43 | 5.05E-05 |  |
| GO_BP_FAT | GO:0044093~positive regulation of molecular function | 59 | 5.92E-05 |  |
| GO_BP_FAT | GO:0043405~regulation of MAP kinase activity | 23 | 1.28E-04 |  |
| GO_BP_FAT | GO:0043085~positive regulation of catalytic activity | 51 | 1.42E-04 |  |
| GO_BP_FAT | GO:0033674~positive regulation of kinase activity | 27 | 2.85E-03 |  |
| GO_BP_FAT | GO:0051347~positive regulation of transferase activity | 27 | 4.85E-03 |  |
| GO_BP_FAT | GO:0045860~positive regulation of protein kinase activity | 25 | 6.22E-03 |  |
| GO_BP_FAT | GO:0043406~positive regulation of MAP kinase activity | 14 | 1.38E-02 |  |
| GO_BP_FAT | GO:0000187~activation of MAPK activity | 10 | 1.09E-01 |  |
| GO_BP_FAT | GO:0032147~activation of protein kinase activity | 10 | 1.20E-01 |  |
| GO_BP_FAT | GO:0000165~MAPKKK cascade | 16 | 3.15E-01 |  |
|  |  |  |  |  |
| Cluster 18 |  |  |  |  |
| GO_BP_FAT | GO:0050900~leukocyte migration | 15 | 1.84E-04 | **3.11** |
| GO_BP_FAT | GO:0060326~cell chemotaxis | 11 | 4.80E-04 |  |
| GO_BP_FAT | GO:0030595~leukocyte chemotaxis | 11 | 4.80E-04 |  |
| GO_BP_FAT | GO:0030593~neutrophil chemotaxis | 7 | 8.76E-03 |  |
|  |  |  |  |  |
| Cluster 19 |  |  |  |  |
| GO_BP_FAT | GO:0031295~T cell costimulation | 7 | 4.91E-05 | **3.07** |
| GO_BP_FAT | GO:0031294~lymphocyte costimulation | 7 | 4.91E-05 |  |
| GO_BP_FAT | GO:0042102~positive regulation of T cell proliferation | 14 | 6.71E-05 |  |
| GO_BP_FAT | GO:0046641~positive regulation of alpha-beta T cell proliferation | 5 | 1.99E-02 |  |
| GO_BP_FAT | GO:0002861~regulation of inflammatory response to antigenic stimulus | 5 | 1.40E-01 |  |
|  |  |  |  |  |
| Cluster 20 |  |  |  |  |
| GO_BP_FAT | GO:0048585~negative regulation of response to stimulus | 21 | 2.11E-05 | **3.06** |
| GO_BP_FAT | GO:0032101~regulation of response to external stimulus | 28 | 2.26E-05 |  |
| GO_BP_FAT | GO:0050727~regulation of inflammatory response | 19 | 3.66E-05 |  |
| GO_BP_FAT | GO:0031348~negative regulation of defense response | 11 | 1.24E-03 |  |
| GO_BP_FAT | GO:0050729~positive regulation of inflammatory response | 9 | 3.79E-03 |  |
| GO_BP_FAT | GO:0050728~negative regulation of inflammatory response | 8 | 8.87E-03 |  |
| GO_BP_FAT | GO:0032102~negative regulation of response to external stimulus | 10 | 1.70E-02 |  |
| GO_BP_FAT | GO:0032103~positive regulation of response to external stimulus | 10 | 2.81E-02 |  |
|  |  |  |  |  |
| Cluster 21 |  |  |  |  |
| GO_MF_FAT | GO:0030246~carbohydrate binding | 67 | 4.10E-07 | **2.88** |
| GO_MF_FAT | GO:0005539~glycosaminoglycan binding | 23 | 7.56E-03 |  |
| GO_MF_FAT | GO:0001871~pattern binding | 25 | 7.84E-03 |  |
| GO_MF_FAT | GO:0030247~polysaccharide binding | 25 | 7.84E-03 |  |
| GO_MF_FAT | GO:0008201~heparin binding | 17 | 2.09E-02 |  |
|  |  |  |  |  |
| Cluster 22 |  |  |  |  |
| GO_MF_FAT | GO:0030695~GTPase regulator activity | 68 | 2.08E-05 | **2.74** |
| GO_MF_FAT | GO:0060589~nucleoside-triphosphatase regulator activity | 68 | 3.60E-05 |  |
| GO_BP_FAT | GO:0051056~regulation of small GTPase mediated signal transduction | 47 | 7.28E-05 |  |
| GO_MF_FAT | GO:0008047~enzyme activator activity | 47 | 4.37E-04 |  |
| GO_MF_FAT | GO:0005085~guanyl-nucleotide exchange factor activity | 31 | 8.08E-04 |  |
| GO_MF_FAT | GO:0005096~GTPase activator activity | 37 | 1.33E-03 |  |
| GO_MF_FAT | GO:0005099~Ras GTPase activator activity | 16 | 4.78E-03 |  |
| GO_MF_FAT | GO:0005083~small GTPase regulator activity | 39 | 5.60E-03 |  |
| GO_BP_FAT | GO:0046578~regulation of Ras protein signal transduction | 33 | 7.40E-03 |  |
| GO_MF_FAT | GO:0005088~Ras guanyl-nucleotide exchange factor activity | 16 | 4.17E-02 |  |
| GO_BP_FAT | GO:0035023~regulation of Rho protein signal transduction | 16 | 6.02E-02 |  |
| GO_MF_FAT | GO:0005089~Rho guanyl-nucleotide exchange factor activity | 13 | 9.60E-02 |  |
|  |  |  |  |  |
| Cluster 23 |  |  |  |  |
| GO_BP_FAT | GO:0032655~regulation of interleukin-12 production | 9 | 8.49E-05 | **2.46** |
| GO_BP_FAT | GO:0045075~regulation of interleukin-12 biosynthetic process | 5 | 1.31E-02 |  |
| GO_BP_FAT | GO:0045084~positive regulation of interleukin-12 biosynthetic process | 4 | 3.62E-02 |  |
|  |  |  |  |  |
| Cluster 24 |  |  |  |  |
| GO_BP_FAT | GO:0035295~tube development | 52 | 9.83E-05 | **2.40** |
| GO_BP_FAT | GO:0060541~respiratory system development | 24 | 1.21E-02 |  |
| GO_BP_FAT | GO:0030324~lung development | 22 | 1.30E-02 |  |
| GO_BP_FAT | GO:0030323~respiratory tube development | 22 | 1.57E-02 |  |
|  |  |  |  |  |
| Cluster 25 |  |  |  |  |
| GO_BP_FAT | GO:0042592~homeostatic process | 95 | 2.84E-04 | **2.39** |
| GO_BP_FAT | GO:0048878~chemical homeostasis | 62 | 1.27E-03 |  |
| GO_BP_FAT | GO:0055080~cation homeostasis | 36 | 1.52E-03 |  |
| GO_BP_FAT | GO:0055066~di-, tri-valent inorganic cation homeostasis | 30 | 1.89E-03 |  |
| GO_BP_FAT | GO:0050801~ion homeostasis | 50 | 3.59E-03 |  |
| GO_BP_FAT | GO:0055065~metal ion homeostasis | 23 | 3.63E-03 |  |
| GO_BP_FAT | GO:0055074~calcium ion homeostasis | 21 | 4.56E-03 |  |
| GO_BP_FAT | GO:0019725~cellular homeostasis | 56 | 5.24E-03 |  |
| GO_BP_FAT | GO:0030003~cellular cation homeostasis | 29 | 6.72E-03 |  |
| GO_BP_FAT | GO:0006873~cellular ion homeostasis | 44 | 7.94E-03 |  |
| GO_BP_FAT | GO:0006875~cellular metal ion homeostasis | 21 | 8.24E-03 |  |
| GO_BP_FAT | GO:0055082~cellular chemical homeostasis | 44 | 1.24E-02 |  |
| GO_BP_FAT | GO:0006874~cellular calcium ion homeostasis | 19 | 1.32E-02 |  |
| GO_BP_FAT | GO:0030005~cellular di-, tri-valent inorganic cation homeostasis | 25 | 1.60E-02 |  |
|  |  |  |  |  |
| Cluster 26 |  |  |  |  |
| GO_BP_FAT | GO:0008354~germ cell migration | 8 | 5.31E-05 | **2.29** |
| GO_BP_FAT | GO:0035234~germ cell programmed cell death | 4 | 1.23E-02 |  |
| GO_BP_FAT | GO:0048070~regulation of pigmentation during development | 5 | 2.86E-02 |  |
| GO_BP_FAT | GO:0010623~developmental programmed cell death | 4 | 3.62E-02 |  |
|  |  |  |  |  |
| Cluster 27 |  |  |  |  |
| GO_BP_FAT | GO:0001782~B cell homeostasis | 9 | 8.49E-05 | **2.28** |
| GO_BP_FAT | GO:0001776~leukocyte homeostasis | 13 | 9.32E-04 |  |
| GO_BP_FAT | GO:0002260~lymphocyte homeostasis | 11 | 1.24E-03 |  |
| GO_BP_FAT | GO:0048872~homeostasis of number of cells | 22 | 1.57E-02 |  |
| GO_BP_FAT | GO:0070227~lymphocyte apoptosis | 5 | 3.91E-02 |  |
| GO_BP_FAT | GO:0043029~T cell homeostasis | 4 | 3.37E-01 |  |
|  |  |  |  |  |
| Cluster 28 |  |  |  |  |
| GO_BP_FAT | GO:0006633~fatty acid biosynthetic process | 21 | 5.88E-04 | **2.22** |
| GO_BP_FAT | GO:0046394~carboxylic acid biosynthetic process | 28 | 4.50E-03 |  |
| GO_BP_FAT | GO:0016053~organic acid biosynthetic process | 28 | 4.50E-03 |  |
| GO_BP_FAT | GO:0006631~fatty acid metabolic process | 34 | 5.30E-03 |  |
| GO_BP_FAT | GO:0008610~lipid biosynthetic process | 40 | 1.27E-01 |  |
|  |  |  |  |  |
| Cluster 29 |  |  |  |  |
| GO_BP_FAT | GO:0002274~myeloid leukocyte activation | 12 | 1.24E-03 | **2.14** |
| GO_BP_FAT | GO:0043011~myeloid dendritic cell differentiation | 5 | 8.01E-03 |  |
| GO_BP_FAT | GO:0001773~myeloid dendritic cell activation | 5 | 8.01E-03 |  |
| GO_BP_FAT | GO:0002573~myeloid leukocyte differentiation | 9 | 3.36E-02 |  |
|  |  |  |  |  |
| Cluster 30 |  |  |  |  |
| GO_BP_FAT | GO:0035295~tube development | 52 | 9.83E-05 | **3.00** |
| GO_BP_FAT | GO:0035239~tube morphogenesis | 34 | 1.59E-03 |  |
| GO_BP_FAT | GO:0035148~tube lumen formation | 16 | 2.01E-03 |  |
| GO_BP_FAT | GO:0001838~embryonic epithelial tube formation | 14 | 6.44E-03 |  |
| GO_BP_FAT | GO:0048729~tissue morphogenesis | 41 | 7.32E-03 |  |
| GO_BP_FAT | GO:0060606~tube closure | 11 | 1.02E-02 |  |
| GO_BP_FAT | GO:0001843~neural tube closure | 11 | 1.02E-02 |  |
| GO_BP_FAT | GO:0001841~neural tube formation | 13 | 1.05E-02 |  |
| GO_BP_FAT | GO:0021915~neural tube development | 18 | 1.12E-02 |  |
| GO_BP_FAT | GO:0014020~primary neural tube formation | 11 | 1.72E-02 |  |
| GO_BP_FAT | GO:0002009~morphogenesis of an epithelium | 30 | 2.08E-02 |  |
| GO_BP_FAT | GO:0016331~morphogenesis of embryonic epithelium | 16 | 2.83E-02 |  |
| GO_BP_FAT | GO:0060562~epithelial tube morphogenesis | 20 | 4.47E-02 |  |
| GO_BP_FAT | GO:0060429~epithelium development | 41 | 5.08E-02 |  |
|  |  |  |  |  |
| Cluster 31 |  |  |  |  |
| GO_BP_FAT | GO:0060348~bone development | 27 | 6.55E-04 | **3.00** |
| GO_BP_FAT | GO:0001503~ossification | 24 | 1.62E-03 |  |
| GO_BP_FAT | GO:0001501~skeletal system development | 43 | 4.73E-02 |  |
| GO_BP_FAT | GO:0001649~osteoblast differentiation | 10 | 8.01E-02 |  |
|  |  |  |  |  |
| Cluster 32 |  |  |  |  |
| GO_MF_FAT | GO:0016502~nucleotide receptor activity | 10 | 2.61E-03 | **2.09** |
| GO_MF_FAT | GO:0001614~purinergic nucleotide receptor activity | 10 | 2.61E-03 |  |
| GO_MF_FAT | GO:0045028~purinergic nucleotide receptor activity, G-protein coupled | 9 | 6.03E-03 |  |
| GO_MF_FAT | GO:0001608~nucleotide receptor activity, G-protein coupled | 9 | 6.03E-03 |  |
| GO_MF_FAT | GO:0001609~adenosine receptor activity, G-protein coupled | 3 | 1.39E-01 |  |
|  |  |  |  |  |
| Cluster 33 |  |  |  |  |
| GO_BP_FAT | GO:0050778~positive regulation of immune response | 30 | 5.85E-04 | **2.05** |
| GO_BP_FAT | GO:0002768~immune response-regulating cell surface receptor signaling pathway | 12 | 8.77E-03 |  |
| GO_BP_FAT | GO:0050852~T cell receptor signaling pathway | 8 | 8.87E-03 |  |
| GO_BP_FAT | GO:0002764~immune response-regulating signal transduction | 13 | 1.22E-02 |  |
| GO_BP_FAT | GO:0002429~immune response-activating cell surface receptor signaling pathway | 11 | 1.45E-02 |  |
| GO_BP_FAT | GO:0002253~activation of immune response | 18 | 1.58E-02 |  |
| GO_BP_FAT | GO:0002757~immune response-activating signal transduction | 12 | 1.69E-02 |  |
| GO_BP_FAT | GO:0050851~antigen receptor-mediated signaling pathway | 10 | 1.70E-02 |  |

^1^ Each row corresponds to a Functional Annotation Tool (FAT) GO category in a cluster.

^2^ GO terms in each cluster.
