# Supplementary material for: Microglia Transcriptome Changes in a Model of Depressive Behavior after Immune Challenge
Source: PLoS One. 2016 Mar 9;11(3):e0150858. doi: 10.1371/journal.pone.0150858 (PMC4784788; doi:10.1371/journal.pone.0150858)
Supplement: S7 Table — (DOCX) [file pone.0150858.s007.docx]

S7 Table. Gene Set Enrichment Analysis (GSEA) categories enriched among transcript isoforms under-expressed (FDR-adjusted P-value < 0.05 and > 10 transcript isoforms) and over- expressed (P-value < 0.01 and > 10 transcript isoforms) in microglia relative to peripheral macrophages in BCG-challenged mice.

| Categories | NG^1^ | P-value | FDR^2^ |
| --- | --- | --- | --- |
| Under-expressed in microglia vs macrophages | | | |
| ADAPTIVE_IMMUNE_RESPONSE | 21 | 0.00E+00 | 0.00E+00 |
| ADAPTIVE_IMMUNE_RESPONSE_GO_0002460 | 20 | 0.00E+00 | 0.00E+00 |
| POSITIVE_REGULATION_OF_IMMUNE_RESPONSE | 20 | 0.00E+00 | 0.00E+00 |
| REGULATION_OF_IMMUNE_RESPONSE | 22 | 0.00E+00 | 0.00E+00 |
| REGULATION_OF_IMMUNE_SYSTEM_PROCESS | 46 | 0.00E+00 | 1.05E-03 |
| CHEMOKINE_RECEPTOR_BINDING | 28 | 0.00E+00 | 1.18E-03 |
| POSITIVE_REGULATION_OF_IMMUNE_SYSTEM_PROCESS | 37 | 0.00E+00 | 1.34E-03 |
| POSITIVE_REGULATION_OF_RESPONSE_TO_STIMULUS | 27 | 0.00E+00 | 1.57E-03 |
| KEGG_PRIMARY_IMMUNODEFICIENCY | 30 | 0.00E+00 | 1.88E-03 |
| KEGG_RIBOSOME | 81 | 0.00E+00 | 1.90E-03 |
| REGULATION_OF_LYMPHOCYTE_ACTIVATION | 28 | 0.00E+00 | 2.07E-03 |
| CELLULAR_DEFENSE_RESPONSE | 38 | 0.00E+00 | 2.28E-03 |
| LYMPHOCYTE_ACTIVATION | 47 | 0.00E+00 | 3.78E-03 |
| G_PROTEIN_COUPLED_RECEPTOR_BINDING | 35 | 0.00E+00 | 4.34E-03 |
| CHEMOKINE_ACTIVITY | 27 | 0.00E+00 | 4.62E-03 |
| POSITIVE_REGULATION_OF_MULTICELLULAR_ORGANISMAL_PROCESS | 47 | 0.00E+00 | 4.63E-03 |
| CYTOKINE_ACTIVITY | 61 | 0.00E+00 | 6.65E-03 |
| B_CELL_ACTIVATION | 18 | 0.00E+00 | 7.59E-03 |
| LOCOMOTORY_BEHAVIOR | 61 | 0.00E+00 | 8.08E-03 |
| LEUKOCYTE_ACTIVATION | 51 | 0.00E+00 | 8.95E-03 |
| KEGG_HEMATOPOIETIC_CELL_LINEAGE | 52 | 0.00E+00 | 9.40E-03 |
| G1_S_TRANSITION_OF_MITOTIC_CELL_CYCLE | 26 | 0.00E+00 | 1.07E-02 |
| CYTOKINE_AND_CHEMOKINE_MEDIATED_SIGNALING_PATHWAY | 15 | 1.45E-02 | 1.35E-02 |
| CELL_ACTIVATION | 54 | 0.00E+00 | 1.36E-02 |
| REGULATION_OF_T_CELL_ACTIVATION | 22 | 0.00E+00 | 1.40E-02 |
| T_CELL_ACTIVATION | 32 | 0.00E+00 | 1.46E-02 |
| REGULATION_OF_RESPONSE_TO_STIMULUS | 35 | 0.00E+00 | 1.80E-02 |
| IMMUNE_EFFECTOR_PROCESS | 23 | 2.70E-02 | 1.82E-02 |
| POSITIVE_REGULATION_OF_LYMPHOCYTE_ACTIVATION | 20 | 0.00E+00 | 2.35E-02 |
| CYTOKINE_METABOLIC_PROCESS | 31 | 0.00E+00 | 2.40E-02 |
| Over-expressed in microglia vs macrophages | | | |
| BRAIN_DEVELOPMENT | 25 | 0.00E+00 | 2.73E-02 |
| CENTRAL_NERVOUS_SYSTEM_DEVELOPMENT | 62 | 0.00E+00 | 3.35E-02 |
| POTASSIUM_ION_TRANSPORT | 21 | 0.00E+00 | 4.81E-02 |
| PHOSPHATASE_REGULATOR_ACTIVITY | 202 | 0.00E+00 | 1.93E-01 |
| POTASSIUM_CHANNEL_ACTIVITY | 246 | 0.00E+00 | 3.54E-01 |
| VOLTAGE_GATED_CATION_CHANNEL_ACTIVITY | 107 | 0.00E+00 | 3.67E-01 |
| PATTERN_SPECIFICATION_PROCESS | 146 | 0.00E+00 | 3.84E-01 |
| NERVOUS_SYSTEM_DEVELOPMENT | 179 | 0.00E+00 | 3.86E-01 |
| TRANSFORMING_GROWTH_FACTOR_BETA_RECEPTOR_SIGNALING_PATHWAY | 208 | 0.00E+00 | 3.94E-01 |
| VOLTAGE_GATED_CHANNEL_ACTIVITY | 100 | 1.00E-03 | 3.67E-01 |
| KEGG_TIGHT_JUNCTION | 88 | 2.00E-03 | 3.51E-01 |
| METAL_ION_TRANSMEMBRANE_TRANSPORTER_ACTIVITY | 125 | 3.00E-03 | 3.77E-01 |
| SYSTEM_PROCESS | 23 | 3.17E-03 | 6.77E-02 |
| TRANSMISSION_OF_NERVE_IMPULSE | 29 | 6.20E-03 | 2.55E-01 |
| CATION_TRANSMEMBRANE_TRANSPORTER_ACTIVITY | 27 | 6.24E-03 | 1.88E-01 |
| ENZYME_LINKED_RECEPTOR_PROTEIN_SIGNALING_PATHWAY | 64 | 7.04E-03 | 3.53E-01 |
| SYNAPTIC_TRANSMISSION | 59 | 7.04E-03 | 3.68E-01 |
| KEGG_AXON_GUIDANCE | 80 | 8.01E-03 | 3.74E-01 |
| KEGG_VASCULAR_SMOOTH_MUSCLE_CONTRACTION | 93 | 8.02E-03 | 3.74E-01 |
| ION_TRANSMEMBRANE_TRANSPORTER_ACTIVITY | 69 | 8.04E-03 | 3.65E-01 |
| NEUROLOGICAL_SYSTEM_PROCESS | 17 | 8.62E-03 | 7.99E-02 |
| SUBSTRATE_SPECIFIC_TRANSMEMBRANE_TRANSPORTER_ACTIVITY | 31 | 9.25E-03 | 2.51E-01 |
| SUBSTRATE_SPECIFIC_TRANSPORTER_ACTIVITY | 19 | 9.64E-03 | 1.90E-01 |

^1^ NG: number of genes.

^2^ FDR: adjusted P-value.
