# Supplementary material for: Microglia Transcriptome Changes in a Model of Depressive Behavior after Immune Challenge
Source: PLoS One. 2016 Mar 9;11(3):e0150858. doi: 10.1371/journal.pone.0150858 (PMC4784788; doi:10.1371/journal.pone.0150858)
Supplement: S8 Table — (DOCX) [file pone.0150858.s008.docx]

S8 Table. Genes exhibiting an alternative splicing event between microglia and peripheral macrophages in BCG-challenged mice including at least two transcript isoforms and at least one over- or under-expressed (FDR-adjusted P-value < 0.05) transcript isoforms between cell types.

| Gene | NCBI Gene Name | Differential Expression | | |
| --- | --- | --- | --- | --- |
|  |  | Under^1^ | Over^2^ | Non^3^ |
| Macf1 | microtubule-actin crosslinking factor 1 | 6 | 10 | 15 |
| Rbm5 | RNA binding motif protein 5 | 6 | 9 | 14 |
| Bag6 | BCL2-associated athanogene 6 | 7 | 7 | 13 |
| Arhgef1 | Rho guanine nucleotide exchange factor (GEF) 1 | 7 | 7 | 11 |
| Rbm39 | RNA binding motif protein 39 | 6 | 7 | 12 |
| Gapvd1 | GTPase activating protein and VPS9 domains 1 | 7 | 5 | 11 |
| Josd2 | Josephin domain containing 2 | 4 | 7 | 10 |
| Lrrc16a | leucine rich repeat containing 16A | 6 | 5 | 9 |
| Rnf220 | ring finger protein 220 | 2 | 8 | 9 |
| Gripap1 | GRIP1 associated protein 1 | 6 | 4 | 9 |
| Anapc1 | anaphase promoting complex subunit 1 | 5 | 5 | 9 |
| Arhgap4 | Rho GTPase activating protein 4 | 2 | 8 | 9 |
| Hnrnpl | heterogeneous nuclear ribonucleoprotein L | 4 | 6 | 9 |
| Il15ra | interleukin 15 receptor, alpha chain | 5 | 5 | 9 |
| Srsf5 | serine/arginine-rich splicing factor 5 | 3 | 7 | 8 |
| Ythdc1 | YTH domain containing 1 | 3 | 6 | 8 |
| Brwd1 | bromodomain and WD repeat domain containing 1 | 4 | 5 | 8 |
| Rtn4 | reticulon 4 | 4 | 5 | 8 |
| Sfrs18 | PNN interacting serine/arginine-rich | 4 | 5 | 8 |
| Wdr13 | WD repeat domain 13 | 5 | 4 | 8 |
| Wnk1 | WNK lysine deficient protein kinase 1 | 5 | 4 | 7 |
| Ctage5 | CTAGE family, member 5 | 3 | 6 | 6 |
| Hivep3 | human immunodeficiency virus type I enhancer binding protein 3 | 4 | 5 | 5 |
| Zmiz1 | zinc finger, MIZ-type containing 1 | 3 | 5 | 7 |
| Stag2 | stromal antigen 2 | 4 | 4 | 7 |
| Ccser2 | coiled-coil serine rich 2 | 4 | 4 | 7 |
| Plgrkt | plasminogen receptor, C-terminal lysine transmembrane protein | 2 | 6 | 7 |
| Tcirg1 | T cell, immune regulator 1, ATPase, H+ transporting, lysosomal V0 protein A3 | 2 | 6 | 7 |
| Wbp1 | WW domain binding protein 1 | 2 | 6 | 7 |
| Dgat1 | diacylglycerol O-acyltransferase 1 | 1 | 7 | 6 |
| Sh3bp1 | SH3-domain binding protein 1 | 1 | 7 | 5 |
| Bcor | BCL6 interacting corepressor | 2 | 5 | 6 |
| Ankrd13d | ankyrin repeat domain 13 family, member D | 0 | 7 | 6 |
| Azin1 | antizyme inhibitor 1 | 3 | 4 | 6 |
| Depdc5 | DEP domain containing 5 | 2 | 5 | 6 |
| Fus | fused in sarcoma | 1 | 6 | 6 |
| Kidins220 | kinase D-interacting substrate 220 | 2 | 5 | 6 |
| Mctp1 | multiple C2 domains, transmembrane 1 | 1 | 6 | 6 |
| Paip1 | polyadenylate binding protein-interacting protein 1 | 5 | 2 | 6 |
| Pcyt2 | phosphate cytidylyltransferase 2, ethanolamine | 3 | 4 | 6 |
| Ppfibp1 | PTPRF interacting protein, binding protein 1 (liprin beta 1) | 4 | 3 | 6 |
| Zfp410 | zinc finger protein 410 | 1 | 6 | 6 |
| Zmym6 | zinc finger, MYM-type 6 | 0 | 7 | 6 |
| Sez6l2 | seizure related 6 homolog like 2 | 0 | 7 | 5 |
| Sh3kbp1 | SH3-domain kinase binding protein 1 | 6 | 1 | 5 |
| Kansl1 | KAT8 regulatory NSL complex subunit 1 | 5 | 1 | 5 |
| Aim2 | absent in melanoma 2 | 3 | 3 | 5 |
| A630001G21Rik | RIKEN cDNA A630001G21 gene | 4 | 2 | 4 |
| Gprasp1 | G protein-coupled receptor associated sorting protein 1 | 2 | 4 | 5 |
| Clcn4-2 | chloride channel 4-2 | 4 | 2 | 5 |
| Flcn | folliculin | 2 | 4 | 4 |
| Scrib | scribbled homolog (Drosophila) | 2 | 4 | 5 |
| Stk25 | serine/threonine kinase 25 (yeast) | 2 | 4 | 5 |
| Tra2b | transformer 2 beta homolog (Drosophila) | 2 | 4 | 5 |
| Cdip1 | cell death inducing Trp53 target 1 | 3 | 3 | 5 |
| Diap2 | diaphanous homolog 2 (Drosophila) | 3 | 3 | 4 |
| Gm129 | circadian associated repressor of transcription | 3 | 3 | 5 |
| Smurf2 | SMAD specific E3 ubiquitin protein ligase 2 | 3 | 3 | 5 |
| Tbkbp1 | TBK1 binding protein 1 | 3 | 3 | 5 |
| Wdr91 | WD repeat domain 91 | 5 | 1 | 5 |
| Camta2 | calmodulin binding transcription activator 2 | 3 | 3 | 5 |
| Dpm1 | dolichol-phosphate (beta-D) mannosyltransferase 1 | 6 | 0 | 5 |
| Lrch3 | eucine-rich repeats and calponin homology (CH) domain containing 3 | 2 | 4 | 5 |
| Luc7l | Luc7 homolog (S. cerevisiae)-like | 3 | 3 | 5 |
| Mapkap1 | mitogen-activated protein kinase associated protein 1 | 2 | 4 | 5 |
| Mcfd2 | multiple coagulation factor deficiency 2 | 5 | 1 | 5 |
| Nfx1 | nuclear transcription factor, X-box binding 1 | 3 | 3 | 5 |
| Opa1 | optic atrophy 1 | 2 | 4 | 5 |
| Rab3gap2 | RAB3 GTPase activating protein subunit 2 | 4 | 2 | 5 |
| Ralgapa1 | Ral GTPase activating protein, alpha subunit 1 | 2 | 4 | 5 |
| Rnf216 | ring finger protein 216 | 3 | 3 | 5 |
| Rsu1 | Ras suppressor protein 1 | 3 | 3 | 5 |
| Sfpq | splicing factor proline/glutamine rich (polypyrimidine tract binding protein associated) | 2 | 4 | 5 |
| Sgk3 | serum/glucocorticoid regulated kinase 3 | 3 | 3 | 5 |
| Ttll4 | tubulin tyrosine ligase-like family, member 4 | 3 | 3 | 5 |
| Znrf1 | zinc and ring finger 1 | 5 | 1 | 5 |
| 4833439L19Rik | RIKEN cDNA 4833439L19 gene | 3 | 3 | 4 |
| Arhgap17 | Rho GTPase activating protein 17 | 3 | 3 | 4 |
| Cdk9 | cyclin-dependent kinase 9 (CDC2-related kinase) | 1 | 5 | 4 |
| Eif4a1 | eukaryotic translation initiation factor 4A1 | 3 | 3 | 4 |
| Sipa1 | signal-induced proliferation associated gene 1 | 0 | 6 | 2 |
| Scamp5 | secretory carrier membrane protein 5 | 0 | 6 | 1 |
| Srsf7 | serine/arginine-rich splicing factor 7 | 4 | 1 | 4 |
| Nlrp1b | NLR family, pyrin domain containing 1B | 0 | 5 | 3 |
| Cdc27 | cell division cycle 27 | 3 | 2 | 4 |
| Esco1 | establishment of cohesion 1 homolog 1 (S. cerevisiae) | 3 | 2 | 4 |
| Fry | furry homolog (Drosophila) | 3 | 2 | 3 |
| Ms4a7 | membrane-spanning 4-domains, subfamily A, member 7 | 4 | 1 | 4 |
| Olfm1 | olfactomedin 1 | 2 | 3 | 4 |
| Prpf39 | PRP39 pre-mRNA processing factor 39 homolog (yeast) | 2 | 3 | 4 |
| Setdb2 | SET domain, bifurcated 2 | 5 | 0 | 4 |
| Slc12a4 | solute carrier family 12, member 4 | 3 | 2 | 4 |
| Slc28a2 | solute carrier family 28 (sodium-coupled nucleoside transporter), member 2 | 5 | 0 | 4 |
| Srsf3 | serine/arginine-rich splicing factor 3 | 1 | 4 | 4 |
| Strada | STE20-related kinase adaptor alpha | 0 | 5 | 4 |
| Susd1 | sushi domain containing 1 | 1 | 4 | 4 |
| Akt1s1 | AKT1 substrate 1 (proline-rich) | 3 | 2 | 4 |
| Amz2 | archaelysin family metallopeptidase 2 | 2 | 3 | 4 |
| Aptx | aprataxin | 3 | 2 | 4 |
| Bptf | bromodomain PHD finger transcription factor | 4 | 1 | 4 |
| Ccm2 | cerebral cavernous malformation 2 | 2 | 3 | 4 |
| Dcaf11 | DDB1 and CUL4 associated factor 11 | 2 | 3 | 4 |
| Evi5l | ecotropic viral integration site 5 like | 2 | 3 | 4 |
| Fam129a | family with sequence similarity 129, member A | 3 | 2 | 4 |
| Fam60a | family with sequence similarity 60, member A | 4 | 1 | 4 |
| Gnas | GNAS (guanine nucleotide binding protein, alpha stimulating) complex locus | 1 | 4 | 4 |
| Jak3 | Janus kinase 3 | 0 | 5 | 4 |
| Lzic | leucine zipper and CTNNBIP1 domain containing | 2 | 3 | 4 |
| Map4 | microtubule-associated protein 4 | 1 | 4 | 4 |
| Mtx1 | metaxin 1 | 3 | 2 | 4 |
| Nsmf | NMDA receptor synaptonuclear signaling and neuronal migration factor | 1 | 4 | 4 |
| Pkp4 | plakophilin 4 | 2 | 3 | 4 |
| Ppp1r18 | protein phosphatase 1, regulatory subunit 18 | 1 | 4 | 4 |
| Rbm10 | RNA binding motif protein 10 | 2 | 3 | 4 |
| Rps6kb1 | ribosomal protein S6 kinase, polypeptide 1 | 3 | 2 | 4 |
| Sh3glb1 | SH3-domain GRB2-like B1 (endophilin) | 2 | 3 | 4 |
| Son | Son DNA binding protein | 1 | 4 | 4 |
| Strn4 | striatin, calmodulin binding protein 4 | 2 | 3 | 4 |
| Tars2 | threonyl-tRNA synthetase 2, mitochondrial (putative) | 0 | 5 | 4 |
| Timm10b | translocase of inner mitochondrial membrane 10B | 2 | 3 | 4 |
| Usf2 | upstream transcription factor 2 | 2 | 3 | 4 |
| Whsc1 | Wolf-Hirschhorn syndrome candidate 1 (human) | 4 | 1 | 4 |
| Wipf1 | WAS/WASL interacting protein family, member 1 | 2 | 3 | 4 |
| Cd300lf | CD300 antigen like family member F | 5 | 0 | 3 |
| Emilin1 | elastin microfibril interfacer 1 | 4 | 1 | 3 |
| Ikbkg | inhibitor of kappaB kinase gamma | 4 | 1 | 3 |
| Pld3 | phospholipase D family, member 3 | 1 | 4 | 3 |
| Rnf111 | ring finger 111 | 3 | 2 | 3 |
| Zgpat | zinc finger, CCCH-type with G patch domain | 0 | 5 | 3 |
| Prdm2 | PR domain containing 2, with ZNF domain | 2 | 2 | 3 |
| Rock2 | Rho-associated coiled-coil containing protein kinase 2 | 2 | 2 | 3 |
| Taf4a | TAF4A RNA polymerase II, TATA box binding protein (TBP)-associated factor | 2 | 2 | 3 |
| Pik3c2b | phosphoinositide-3-kinase, class 2, beta polypeptide | 2 | 2 | 3 |
| Rgs2 | regulator of G-protein signaling 2 | 0 | 4 | 3 |
| Apbb1ip | amyloid beta (A4) precursor protein-binding, family B, member 1 interacting protein | 2 | 2 | 3 |
| Armc8 | armadillo repeat containing 8 | 2 | 2 | 3 |
| Kdm3a | lysine (K)-specific demethylase 3A | 1 | 3 | 3 |
| Lsm6 | LSM6 homolog, U6 small nuclear RNA associated (S. cerevisiae) | 0 | 4 | 3 |
| Mtpap | mitochondrial poly(A) polymerase | 1 | 3 | 3 |
| Trpv2 | transient receptor potential cation channel, subfamily V, member 2 | 2 | 2 | 3 |
| Zfand6 | zinc finger, AN1-type domain 6 | 2 | 2 | 3 |
| Adamts16 | a disintegrin-like and metallopeptidase (reprolysin type) with thrombospondin type 1 motif, 16 | 0 | 4 | 3 |
| Arid2 | AT rich interactive domain 2 (ARID, RFX-like) | 2 | 2 | 3 |
| Casp8 | caspase 8 | 1 | 3 | 3 |
| Tbpl1 | TATA box binding protein-like 1 | 2 | 2 | 3 |
| Acbd5 | acyl-Coenzyme A binding domain containing 5 | 2 | 2 | 3 |
| Clcn3 | chloride channel 3 | 2 | 2 | 3 |
| Dbndd2 | dysbindin (dystrobrevin binding protein 1) domain containing 2 | 1 | 3 | 3 |
| Denr | density-regulated protein | 3 | 1 | 3 |
| Elf2 | E74-like factor 2 | 3 | 1 | 3 |
| Fam118a | family with sequence similarity 118, member A | 1 | 3 | 3 |
| Fgfr1op | Fgfr1 oncogene partner | 2 | 2 | 2 |
| Gbp4 | guanylate binding protein 4 | 2 | 2 | 3 |
| Kif13a | kinesin family member 13A | 2 | 2 | 3 |
| Lsr | lipolysis stimulated lipoprotein receptor | 2 | 2 | 3 |
| Mbd4 | methyl-CpG binding domain protein 4 | 1 | 3 | 3 |
| Men1 | multiple endocrine neoplasia 1 | 2 | 2 | 3 |
| Nme5 | NME/NM23 family member 5 | 2 | 2 | 3 |
| Nos1ap | nitric oxide synthase 1 (neuronal) adaptor protein | 0 | 4 | 3 |
| Pex16 | peroxisomal biogenesis factor 16 | 2 | 2 | 3 |
| Plekha2 | pleckstrin homology domain-containing, family A (phosphoinositide binding specific) member 2 | 3 | 1 | 3 |
| Ppp1r12c | protein phosphatase 1, regulatory (inhibitor) subunit 12C | 0 | 4 | 3 |
| Ranbp3 | RAN binding protein 3 | 3 | 1 | 3 |
| Rapgef6 | Rap guanine nucleotide exchange factor (GEF) 6 | 3 | 1 | 3 |
| Rasa4 | RAS p21 protein activator 4 | 2 | 2 | 3 |
| Rfx7 | regulatory factor X, 7 | 3 | 1 | 3 |
| Ripk1 | receptor (TNFRSF)-interacting serine-threonine kinase 1 | 3 | 1 | 3 |
| Serpina3g | serine (or cysteine) peptidase inhibitor, clade A, member 3G | 4 | 0 | 2 |
| Slc26a6 | solute carrier family 26, member 6 | 3 | 1 | 3 |
| Slc38a11 | solute carrier family 38, member 11 | 0 | 4 | 3 |
| Srsf2 | serine/arginine-rich splicing factor 2 | 1 | 3 | 3 |
| Stau2 | staufen (RNA binding protein) homolog 2 (Drosophila) | 0 | 4 | 3 |
| Syncrip | synaptotagmin binding, cytoplasmic RNA interacting protein | 4 | 0 | 3 |
| Tbc1d10a | TBC1 domain family, member 10a | 0 | 4 | 3 |
| Tbcd | tubulin-specific chaperone d | 1 | 3 | 3 |
| Top3b | topoisomerase (DNA) III beta | 0 | 4 | 3 |
| Traf5 | TNF receptor-associated factor 5 | 2 | 2 | 3 |
| Ube2m | ubiquitin-conjugating enzyme E2M | 2 | 2 | 3 |
| Ube2w | ubiquitin-conjugating enzyme E2W (putative) | 3 | 1 | 3 |
| Urb1 | URB1 ribosome biogenesis 1 homolog (S. cerevisiae) | 2 | 2 | 3 |
| Usf1 | upstream transcription factor 1 | 1 | 3 | 3 |
| Vps37a | vacuolar protein sorting 37A (yeast) | 3 | 1 | 3 |
| Ylpm1 | YLP motif containing 1 | 2 | 2 | 3 |
| Zfp592 | zinc finger protein 592 | 3 | 1 | 3 |
| Bcat2 | branched chain aminotransferase 2, mitochondrial | 2 | 2 | 2 |
| C230081A13Rik | pseudopodium-enriched atypical kinase 1 | 4 | 0 | 2 |
| Nfatc1 | nuclear factor of activated T cells, cytoplasmic, calcineurin dependent 1 | 2 | 2 | 2 |
| Slc4a7 | solute carrier family 4, sodium bicarbonate cotransporter, member 7 | 3 | 1 | 2 |
| Tmem106a | transmembrane protein 106A | 4 | 0 | 2 |
| Brat1 | BRCA1-associated ATM activator 1 | 0 | 4 | 1 |
| Leprel2 | prolyl 3-hydroxylase 3 | 0 | 4 | 1 |
| Zfp691 | zinc finger protein 691 | 0 | 4 | 1 |
| Fgd3 | FYVE, RhoGEF and PH domain containing 3 | 1 | 2 | 2 |
| Hnrnpa2b1 | heterogeneous nuclear ribonucleoprotein A2/B1 | 2 | 1 | 2 |
| Vegfb | vascular endothelial growth factor B | 0 | 3 | 1 |
| Pisd-ps2 | phosphatidylserine decarboxylase, pseudogene 2 | 0 | 3 | 2 |
| Gm4951 | predicted gene 4951 | 3 | 0 | 2 |
| Mark3 | MAP/microtubule affinity-regulating kinase 3 | 1 | 2 | 2 |
| Pnrc1 | proline-rich nuclear receptor coactivator 1 | 2 | 1 | 2 |
| Zdhhc5 | zinc finger, DHHC domain containing 5 | 3 | 0 | 2 |
| Akap8l | A kinase (PRKA) anchor protein 8-like | 0 | 3 | 2 |
| Bbc3 | BCL2 binding component 3 | 1 | 2 | 2 |
| Cass4 | Cas scaffolding protein family member 4 | 1 | 2 | 2 |
| E4f1 | E4F transcription factor 1 | 1 | 2 | 2 |
| Pcf11 | cleavage and polyadenylation factor subunit homolog (S. cerevisiae) | 1 | 2 | 2 |
| Prim2 | DNA primase, p58 subunit | 1 | 2 | 2 |
| Ptplad2 | 3-hydroxyacyl-CoA dehydratase 4 | 1 | 2 | 2 |
| Vps45 | vacuolar protein sorting 45 (yeast) | 0 | 3 | 2 |
| Atxn1 | ataxin 1 | 1 | 2 | 2 |
| C130050O18Rik | RIKEN cDNA C130050O18 gene | 0 | 3 | 2 |
| Ccdc142 | coiled-coil domain containing 142 | 1 | 2 | 2 |
| Naip5 | NLR family, apoptosis inhibitory protein 5 | 0 | 3 | 2 |
| Noc2l | nucleolar complex associated 2 homolog (S. cerevisiae) | 1 | 2 | 2 |
| Tefm | transcription elongation factor, mitochondrial | 0 | 3 | 2 |
| Acap3 | ArfGAP with coiled-coil, ankyrin repeat and PH domains 3 | 2 | 1 | 2 |
| Ahcyl2 | S-adenosylhomocysteine hydrolase-like 2 | 2 | 1 | 2 |
| Anapc15 | anaphase prompoting complex C subunit 15 | 1 | 2 | 2 |
| Ankrd44 | ankyrin repeat domain 44 | 1 | 2 | 2 |
| Atp5g1 | ATP synthase, H+ transporting, mitochondrial F0 complex, subunit C1 (subunit 9) | 1 | 2 | 2 |
| Bcas2 | breast carcinoma amplified sequence 2 | 1 | 2 | 2 |
| Capn7 | calpain 7 | 2 | 1 | 2 |
| Chkb | choline kinase beta | 2 | 1 | 2 |
| Cnnm3 | cyclin M3 | 0 | 3 | 2 |
| Cox16 | cytochrome c oxidase assembly protein 16 | 2 | 1 | 2 |
| Crebrf | CREB3 regulatory factor | 3 | 0 | 2 |
| Cspp1 | centrosome and spindle pole associated protein 1 | 2 | 1 | 2 |
| D130020L05Rik | RIKEN cDNA D130020L05 gene | 2 | 1 | 2 |
| D1Ertd622e | DNA segment, Chr 1, ERATO Doi 622, expressed | 1 | 2 | 2 |
| D4Wsu53e | arginine/serine rich protein 1 | 2 | 1 | 2 |
| Dnajc8 | DnaJ (Hsp40) homolog, subfamily C, member 8 | 1 | 2 | 2 |
| Elf1 | E74-like factor 1 | 1 | 2 | 2 |
| Extl2 | exostoses (multiple)-like 2 | 1 | 2 | 2 |
| Fam173b | family with sequence similarity 173, member B | 0 | 3 | 2 |
| Foxp1 | forkhead box P1 | 3 | 0 | 2 |
| Fzr1 | fizzy/cell division cycle 20 related 1 (Drosophila) | 2 | 1 | 2 |
| Gal3st4 | galactose-3-O-sulfotransferase 4 | 0 | 3 | 2 |
| Gngt2 | guanine nucleotide binding protein (G protein), gamma transducing activity polypeptide 2 | 2 | 1 | 2 |
| H2-DMb2 | histocompatibility 2, class II, locus Mb2 | 2 | 1 | 2 |
| Ift46 | intraflagellar transport 46 | 0 | 3 | 2 |
| Inpp1 | inositol polyphosphate-1-phosphatase | 1 | 2 | 2 |
| Itga1 | integrin alpha 1 | 2 | 1 | 2 |
| Lmbr1 | limb region 1 | 2 | 1 | 2 |
| Lss | lanosterol synthase | 1 | 2 | 2 |
| Mad2l2 | MAD2 mitotic arrest deficient-like 2 | 1 | 2 | 2 |
| Mbtps2 | membrane-bound transcription factor peptidase, site 2 | 2 | 1 | 2 |
| Minos1 | mitochondrial inner membrane organizing system 1 | 1 | 2 | 2 |
| Mrgbp | MRG/MORF4L binding protein | 2 | 1 | 2 |
| Ms4a4c | membrane-spanning 4-domains, subfamily A, member 4C | 3 | 0 | 2 |
| Mtmr2 | myotubularin related protein 2 | 1 | 2 | 2 |
| Nck1 | non-catalytic region of tyrosine kinase adaptor protein 1 | 2 | 1 | 2 |
| Nlrx1 | NLR family member X1 | 1 | 2 | 2 |
| Nxt2 | nuclear transport factor 2-like export factor 2 | 2 | 1 | 2 |
| Otub2 | OTU domain, ubiquitin aldehyde binding 2 | 1 | 2 | 2 |
| Pigk | phosphatidylinositol glycan anchor biosynthesis, class K | 1 | 2 | 2 |
| Pilrb2 | paired immunoglobin-like type 2 receptor beta 2 | 3 | 0 | 2 |
| Poc1a | POC1 centriolar protein homolog A (Chlamydomonas) | 2 | 1 | 2 |
| Prlr | prolactin receptor | 0 | 3 | 2 |
| Rabepk | Rab9 effector protein with kelch motifs | 2 | 1 | 2 |
| Rdh5 | retinol dehydrogenase 5 | 0 | 3 | 2 |
| Riok2 | chromodomain helicase DNA binding protein 1 | 1 | 2 | 2 |
| Rps6ka2 | ribosomal protein S6 kinase, polypeptide 2 | 3 | 0 | 2 |
| Sec24c | Sec24 related gene family, member C (S. cerevisiae) | 2 | 1 | 2 |
| Sirpb1b | signal-regulatory protein beta 1B | 2 | 1 | 2 |
| Sirt2 | sirtuin 2 | 2 | 1 | 2 |
| Slc4a2 | solute carrier family 4 (anion exchanger), member 2 | 1 | 2 | 2 |
| Smarcc2 | SWI/SNF related, matrix associated, actin dependent regulator of chromatin, subfamily c, member 2 | 1 | 2 | 1 |
| Smim1 | small integral membrane protein 1 | 1 | 2 | 2 |
| Snhg8 | small nucleolar RNA host gene 8 | 1 | 2 | 2 |
| Snrnp35 | small nuclear ribonucleoprotein 35 (U11/U12) | 3 | 0 | 2 |
| Sra1 | steroid receptor RNA activator 1 | 3 | 0 | 2 |
| Stau1 | staufen (RNA binding protein) homolog 1 (Drosophila) | 2 | 1 | 2 |
| Tada3 | transcriptional adaptor 3 | 2 | 1 | 2 |
| Taf1c | TATA box binding protein (Tbp)-associated factor, RNA polymerase I, C | 1 | 2 | 2 |
| Tbc1d10c | TBC1 domain family, member 10c | 3 | 0 | 2 |
| Ttll3 | tubulin tyrosine ligase-like family, member 3 | 0 | 3 | 2 |
| Vwa5a | von Willebrand factor A domain containing 5A | 0 | 3 | 2 |
| Wrap53 | WD repeat containing, antisense to Trp53 | 2 | 1 | 2 |
| Zbtb6 | zinc finger and BTB domain containing 6 | 3 | 0 | 2 |
| Zfp384 | zinc finger protein 384 | 2 | 1 | 2 |
| Zfp398 | zinc finger protein 398 | 0 | 3 | 1 |
| E330020D12Rik | Riken cDNA E330020D12 gene | 1 | 2 | 1 |
| Gm20605 | predicted gene 20605 | 0 | 3 | 1 |
| Gpd1 | glycerol-3-phosphate dehydrogenase 1 (soluble) | 0 | 3 | 1 |
| Mat2b | methionine adenosyltransferase II, beta | 2 | 1 | 1 |
| Nmt1 | N-myristoyltransferase 1 | 0 | 3 | 1 |
| Pcolce | procollagen C-endopeptidase enhancer protein | 0 | 3 | 1 |
| Pyroxd2 | pyridine nucleotide-disulphide oxidoreductase domain 2 | 0 | 3 | 1 |
| Spsb3 | splA/ryanodine receptor domain and SOCS box containing 3 | 2 | 1 | 1 |
| Urgcp | upregulator of cell proliferation | 2 | 1 | 1 |
| Xbp1 | X-box binding protein 1 | 1 | 2 | 1 |
| Kin | antigenic determinant of rec-A protein | 1 | 1 | 1 |
| Msantd2 | Myb/SANT-like DNA-binding domain containing 2 [ | 1 | 1 | 1 |
| Cdkn1b | cyclin-dependent kinase inhibitor 1B | 1 | 1 | 1 |
| Exoc3 | exocyst complex component 3 | 1 | 1 | 1 |
| Fam49b | family with sequence similarity 49, member B | 1 | 1 | 1 |
| Ak8 | adenylate kinase 8 | 1 | 1 | 1 |
| Fam102b | family with sequence similarity 102, member B | 1 | 1 | 1 |
| Gstt2 | glutathione S-transferase, theta 2 | 1 | 1 | 1 |
| Heatr1 | HEAT repeat containing 1 | 1 | 1 | 1 |
| Sf3b3 | splicing factor 3b, subunit 3 | 1 | 1 | 1 |
| Tut1 | terminal uridylyl transferase 1, U6 snRNA-specific | 1 | 1 | 1 |
| C5ar2 | complement component 5a receptor 2 | 0 | 2 | 1 |
| Foxk1 | forkhead box K1 | 2 | 0 | 1 |
| Gpr84 | G protein-coupled receptor 84 | 1 | 1 | 1 |
| Kcnd1 | potassium voltage-gated channel, Shal-related family, member 1 | 0 | 2 | 1 |
| Kctd6 | potassium channel tetramerisation domain containing 6 | 1 | 1 | 1 |
| Klf10 | Kruppel-like factor 10 | 1 | 1 | 1 |
| Klhl28 | kelch-like 28 | 1 | 1 | 1 |
| Phpt1 | phosphohistidine phosphatase 1 | 0 | 2 | 1 |
| Pptc7 | PTC7 protein phosphatase homolog (S. cerevisiae) | 1 | 1 | 1 |
| Rbm15 | RNA binding motif protein 15 | 2 | 0 | 1 |
| Tnfrsf12a | tumor necrosis factor receptor superfamily, member 12a | 1 | 1 | 1 |
| Wdr74 | WD repeat domain 74 | 1 | 1 | 1 |
| Abca13 | ATP-binding cassette, sub-family A (ABC1), member 13 | 1 | 1 | 1 |
| Acer3 | alkaline ceramidase 3 | 1 | 1 | 1 |
| Acot11 | acyl-CoA thioesterase 11 | 1 | 1 | 1 |
| Afmid | arylformamidase | 0 | 2 | 1 |
| Alox5 | arachidonate 5-lipoxygenase | 0 | 2 | 1 |
| Anapc11 | anaphase promoting complex subunit 11 | 1 | 1 | 1 |
| Arpc4 | actin related protein 2/3 complex, subunit 4 | 2 | 0 | 1 |
| Bfar | bifunctional apoptosis regulator | 2 | 0 | 1 |
| Camta1 | calmodulin binding transcription activator 1 | 2 | 0 | 1 |
| Cebpg | CCAAT/enhancer binding protein (C/EBP), gamma | 1 | 1 | 1 |
| Cnep1r1 | CTD nuclear envelope phosphatase 1 regulatory subunit 1 | 1 | 1 | 1 |
| Cnnm4 | cyclin M | 1 | 1 | 1 |
| Cnp | 2',3'-cyclic nucleotide 3' phosphodiesterase | 0 | 2 | 1 |
| Coil | coilin | 1 | 1 | 1 |
| Ctla2b | cytotoxic T lymphocyte-associated protein 2 beta | 2 | 0 | 1 |
| Diap3 | diaphanous homolog 3 (Drosophila) | 1 | 1 | 1 |
| Dner | delta/notch-like EGF-related receptor | 1 | 1 | 1 |
| Dpp3 | dipeptidylpeptidase 3 | 1 | 1 | 1 |
| Dync1i2 | dynein cytoplasmic 1 intermediate chain 2 | 1 | 1 | 1 |
| Edem1 | ER degradation enhancer, mannosidase alpha-like 1 | 0 | 2 | 1 |
| Emcn | endomucin | 0 | 2 | 1 |
| Fcgr2b | Fc receptor, IgG, low affinity IIb | 2 | 0 | 1 |
| Frmpd4 | FERM and PDZ domain containing 4 | 0 | 2 | 1 |
| Gas2 | growth arrest specific 2 | 2 | 0 | 1 |
| Gimap7 | GTPase, IMAP family member 7 | 2 | 0 | 1 |
| Gjb1 | gap junction protein, beta 1 | 0 | 2 | 1 |
| Glod4 | glyoxalase domain containing 4 | 1 | 1 | 1 |
| Gm10451 | predicted gene 10451 | 2 | 0 | 1 |
| Gm14420 | predicted gene 14420 | 1 | 1 | 1 |
| Hapln2 | hyaluronan and proteoglycan link protein 2 | 0 | 2 | 1 |
| Heyl | hairy/enhancer-of-split related with YRPW motif-like | 0 | 2 | 1 |
| Hook3 | hook homolog 3 (Drosophila) | 2 | 0 | 1 |
| Hspa5 | heat shock protein 5 | 1 | 1 | 1 |
| Ifi204 | interferon activated gene 204 | 2 | 0 | 1 |
| Jmjd6 | jumonji domain containing 6 | 0 | 2 | 1 |
| Kcne4 | potassium voltage-gated channel, Isk-related subfamily, gene 4 | 0 | 2 | 1 |
| Klf4 | Kruppel-like factor 4 (gut) | 0 | 2 | 1 |
| Lnp | limb and neural patterns | 1 | 1 | 1 |
| Map3k12 | mitogen-activated protein kinase kinase kinase 12 | 1 | 1 | 1 |
| Mdk | midkine | 0 | 2 | 1 |
| Naip1 | NLR family, apoptosis inhibitory protein 1 | 1 | 1 | 1 |
| Nemf | nuclear export mediator factor | 1 | 1 | 1 |
| Nuak2 | NUAK family, SNF1-like kinase, 2 | 0 | 2 | 1 |
| Pcyox1l | prenylcysteine oxidase 1 like | 0 | 2 | 1 |
| Phf20 | PHD finger protein 20 | 1 | 1 | 1 |
| Pkn3 | protein kinase N3 | 0 | 2 | 1 |
| Pole3 | polymerase (DNA directed), epsilon 3 (p17 subunit) | 2 | 0 | 1 |
| Ppp1r13l | protein phosphatase 1, regulatory (inhibitor) subunit 13 like | 1 | 1 | 1 |
| Prr13 | proline rich 13 | 2 | 0 | 1 |
| Psmd11 | proteasome (prosome, macropain) 26S subunit, non-ATPase, 11 | 1 | 1 | 1 |
| Rab40c | Rab40C, member RAS oncogene family | 0 | 2 | 1 |
| Rbmxl1 | RNA binding motif protein, X linked-like-1 | 1 | 1 | 1 |
| Reep3 | receptor accessory protein 3 | 1 | 1 | 1 |
| Renbp | renin binding protein | 2 | 0 | 1 |
| Rnf4 | ring finger protein 4 | 2 | 0 | 1 |
| Rrh | retinal pigment epithelium derived rhodopsin homolog | 0 | 2 | 1 |
| Scarf1 | scavenger receptor class F, member 1 | 2 | 0 | 1 |
| Smc1a | structural maintenance of chromosomes 1A | 1 | 1 | 1 |
| Spg7 | spastic paraplegia 7 homolog (human) | 1 | 1 | 1 |
| Ssh1 | slingshot homolog 1 (Drosophila) | 1 | 1 | 1 |
| Tdrd7 | tudor domain containing 7 | 1 | 1 | 1 |
| Thap4 | THAP domain containing 4 | 1 | 1 | 1 |
| Thoc3 | THO complex 3 | 1 | 1 | 1 |
| Thrsp | thyroid hormone responsive | 0 | 2 | 1 |
| Tnfrsf23 | tumor necrosis factor receptor superfamily, member 23 | 1 | 1 | 1 |
| Trex1 | three prime repair exonuclease 1 | 2 | 0 | 1 |
| Trim33 | tripartite motif-containing 33 | 1 | 1 | 1 |
| Trip6 | thyroid hormone receptor interactor 6 | 0 | 2 | 1 |
| Ube2d2a | ubiquitin-conjugating enzyme E2D 2A | 1 | 1 | 1 |
| Ubqln1 | ubiquilin 1 | 1 | 1 | 1 |
| Wfdc2 | WAP four-disulfide core domain 2 | 0 | 2 | 1 |
| Yrdc | yrdC domain containing (E.coli) | 2 | 0 | 1 |
| Zfp628 | zinc finger protein 628 | 2 | 0 | 1 |
| Zic1 | zinc finger protein of the cerebellum 1 | 0 | 2 | 1 |

^1^Over: transcript isoforms over-expressed in microglia

^2^Under: transcript isoforms under-expressed in microglia

^3^Non: not differentially expressed transcript isoforms (FDR-adjusted P-value < 0.05)
