# Supplementary material for: Microglia Transcriptome Changes in a Model of Depressive Behavior after Immune Challenge
Source: PLoS One. 2016 Mar 9;11(3):e0150858. doi: 10.1371/journal.pone.0150858 (PMC4784788; doi:10.1371/journal.pone.0150858)
Supplement: S9 Table — (DOCX) [file pone.0150858.s009.docx]

S9 Table. Functional cluster (DAVID Enrichment score ES > 2) of categories enriched by transcript isoforms expressed solely in microglia cells in BCG-challenged mice.

| Cell type and Category^1^ | Terms^2^ | Count | P-value | ES |
| --- | --- | --- | --- | --- |
| Unique to Microglia BCG | | | | |
| Cluster 1 |  |  |  |  |
| GO_MF_FAT | GO:0005216~ion channel activity | 13 | 7.38E-05 | 2.06 |
| GO_MF_FAT | GO:0022838~substrate specific channel activity | 13 | 9.91E-05 |  |
| GO_MF_FAT | GO:0022803~passive transmembrane transporter activity | 13 | 1.13E-04 |  |
| GO_MF_FAT | GO:0015267~channel activity | 13 | 1.13E-04 |  |
| GO_MF_FAT | GO:0008509~anion transmembrane transporter activity | 8 | 1.74E-04 |  |
| GO_MF_FAT | GO:0005254~chloride channel activity | 6 | 2.75E-04 |  |
| GO_MF_FAT | GO:0005253~anion channel activity | 6 | 3.91E-04 |  |
| GO_BP_FAT | GO:0006811~ion transport | 17 | 6.82E-04 |  |
| GO_BP_FAT | GO:0006820~anion transport | 7 | 1.16E-03 |  |
| GO_BP_FAT | GO:0006821~chloride transport | 5 | 2.24E-03 |  |
| GO_MF_FAT | GO:0031404~chloride ion binding | 5 | 3.30E-03 |  |
| GO_MF_FAT | GO:0043168~anion binding | 5 | 3.67E-03 |  |
| GO_MF_FAT | GO:0022836~gated channel activity | 9 | 3.95E-03 |  |
| GO_BP_FAT | GO:0015698~inorganic anion transport | 5 | 6.78E-03 |  |
| GO_MF_FAT | GO:0022832~voltage-gated channel activity | 5 | 7.89E-02 |  |
| GO_MF_FAT | GO:0005244~voltage-gated ion channel activity | 5 | 7.89E-02 |  |
| GO_MF_FAT | GO:0005267~potassium channel activity | 4 | 1.11E-01 |  |
| GO_BP_FAT | GO:0006813~potassium ion transport | 4 | 1.77E-01 |  |
| GO_MF_FAT | GO:0005261~cation channel activity | 5 | 1.86E-01 |  |
| GO_MF_FAT | GO:0046873~metal ion transmembrane transporter activity | 5 | 2.67E-01 |  |
| GO_MF_FAT | GO:0030955~potassium ion binding | 3 | 2.89E-01 |  |
| GO_BP_FAT | GO:0015672~monovalent inorganic cation transport | 4 | 5.20E-01 |  |
| GO_MF_FAT | GO:0031420~alkali metal ion binding | 3 | 5.58E-01 |  |
| GO_BP_FAT | GO:0006812~cation transport | 5 | 6.91E-01 |  |
| GO_BP_FAT | GO:0030001~metal ion transport | 4 | 7.68E-01 |  |

^1^ Each row corresponds to a Functional Annotation Tool (FAT) GO category inside a cluster.

^2^ GO terms in each cluster.
